# Development of a universal imaging “phenome” using shape, appearance and motion (SAM) features and the SAM Phenotype Observation Tool (SPOT)

**DOI:** 10.1038/s41467-026-75505-8

**Published:** 2026-08-17

**Authors:** Felix Y. Zhou, Adam Norton-Steele, Lewis Marsh, Helen M. Byrne, Heather A. Harrington, Xin Lu

**Affiliations:** 1 Ludwig Institute for Cancer Research, Nuffield Department of Medicine, University of Oxford, Oxford, UK; 2 Lyda Hill Department of Bioinformatics, University of Texas Southwestern Medical Center, Dallas, TX USA; 3 Center for Computational Systems Biology, Vanderbilt University, Nashville, TN USA; 4 Vanderbilt Ingram Cancer Center, Vanderbilt University Medical Center, Nashville, TN USA; 5 Mathematical Institute, University of Oxford, Oxford, UK; 6 Centre for Systems Biology Dresden, Dresden, 01307 Germany; 7 Technische Universität Dresden, Dresden, 01062 Germany; 8 Present Address: Department of Cell & Developmental Biology, Vanderbilt University, Nashville, TN USA; 9 Present Address: Novo Nordisk Research Centre Oxford (NNRCO), Oxford, UK; 10 Present Address: Max Planck Institute of Molecular Cell Biology and Genetics, Dresden, Germany

**Keywords:** Data processing, Software, High-throughput screening

## Abstract

Cells are plastic, highly heterogeneous and change over time. High-content timelapse imaging promises to reveal dynamic cell behaviors, enabling more accurate identification of cell state and cell fate prediction for biological hypothesis generation and perturbation screens. To empower live-cell imaging based screening, we report the development of (1) a Shape, Appearance, Motion (SAM) “phenome”; a universal set of 2185 image-derived features that act as a image-“transcriptome” to comprehensively quantify an object’s instantaneous phenotype; (2) the SAM-Phenotype-Observation-Tool (SPOT), for image-“sequencing” analysis of phenomes. We validate the effectiveness of unbiased SAM-SPOT workflow on publicly available computer vision and 2D single cell imaging datasets. Importantly, we demonstrate that SAM-phenome outperforms features generated by deep learning AI models trained on >1 million fixed single cell and >5000 single cell video frames, respectively. SAM-phenome and SPOT deliver high-throughput, object-treatment-agnostic, comprehensive screening readouts of dynamics, promising to advance novel molecular target discovery and new medicine development.

## Abstract (web summary)

Zhou, Norton-Steele and colleagues report a universal single-object imaging phenome and standardized high-throughput workflow for timelapse analysis that accounts for phenotypic heterogeneity, promising to advance target discovery and new medicine development.

## Introduction

Molecular sequencing technologies, particularly scRNA-seq, have transformed our ability to identify previously unknown and rare cell types and states^1–3^. The ability to unbiasedly and simultaneously measure hundreds of transcripts within a cell (average 200–6000 transcripts/cell) regardless of cell and tissue type, to map the transcripts onto a standard reference genome, using widely-accessible tools^4,5^ and implementing a standardized analytical workflow for quantification^6^, enables the systematic identification of known, and the discovery of unknown, genotypes. Unfortunately, scRNA-seq profiles only a single fixed timepoint, and destroys the sample in the process. Therefore, it fundamentally lacks the temporal continuity to assess individual cell trajectories and cell fates, and to filter out the many identified genetic targets that do not impact the final phenotype^7,8^. Moreover, the resolution of scRNA-seq analysis is fixed to that of a single cell. Many biological objects, such as multicellular organoids, cannot be measured as a single unit.

In contrast, timelapse imaging, particularly label-free microscopy, is non-destructive and offers an unmatched ability to simultaneously capture cell behaviors at both short timescales, with fast acquisition rate, and long timescales, imaging over days and weeks^9^. However, computational analyses of the imaged data lack comprehensive phenotypic readouts that are agnostic of biological process, treatment condition and acquisition parameters. This has restricted studies to either developing simplified analytics targeting a specific or limited set of phenotypes^10,11^, biological processes^12^, or developing experimental setups that produce binary-like yes/no phenotypic readouts, primarily based on fluorescent tagging of proteins-of-interest^10,13,14^. Unfortunately, the number of simultaneously visualized markers is fundamentally restricted by overlap of excitation and emission spectra and is limited even if spectral unmixing^15^ and temporal encoding^16^ are used. All reporters must further be designed or selected on a per marker basis, and be compatible with live-cell imaging which requires significant specialized knowledge^17–20^. Importantly, all fluorescent reporters photobleach over time, and cells suffer phototoxicity, manifesting artefactual non-physiologically-relevant behaviors^21^. All these underline the unmet need to develop a sufficiently comprehensive direct image-based readout from label-free bright-field live cell time-lapse imaging, suitable for discovering and characterizing diverse phenotypes in a high-content screen. To develop this readout, we took inspiration from scRNA-seq.

The success of scRNA-seq is due to standardization. First, the prior establishment of reference organism genomes^22–24^ provides a standardized nomenclature, enabling comparison and pooling of scRNA-seq datasets. Crucially, scRNAseq establishes standard experimental protocols to detect, for each cell, the expression of the same standardized ensemble of transcripts comprising the reference transcriptome, without any prior knowledge of the cell type undergoing sequencing^25^. The comprehensiveness of the transcriptome and unbiased detection of all potential transcripts means that scRNA-seq is equally applicable to the analysis of known biological function or to the discovery and characterization of novel biological pathways and cellular functions. Moreover, it encourages data reuse, driving the establishment of cell atlases^26–28^. Secondly, the establishment of a standardized simple, modular workflow, comprising clustering, dimensionality reduction, and pseudotime analysis with necessary software tools^4–6^, democratizes scRNA-seq data analysis for non-specialists.

Shape, appearance and motion (SAM) are three foundational object characteristics that can be measured directly in video frames to uniquely characterize instantaneous object state. Multidimensional SAM assessment of a moving multicellular object, such as a worm or an organoid, captures image features that can be used to distinguish changes in interactions between cells and those with the surrounding environment^11,29^. A cell’s genetic makeup shapes its ability to respond to environmental stimuli, while environmental cues can also sculpt a cell’s signaling networks^8,30,31^. Dynamic cross-talk between environmental perturbations and genetic alterations lead to complex, dynamic, non-linear genotype-phenotype relationships that together determine the ultimate cellular response^32,33^. The ability to measure SAM simultaneously and quantitatively constitute a readily accessible, dynamic, image-based readout that could potentially discriminate phenotypic changes caused by changes in complex genetic circuits or in environmental factors^11,29^. Especially, assessing SAM over long-time horizons would enable more accurate discovery of novel predictors of the fate of a moving multicellular object or organism.

To date, in contrast to scRNA-seq, no standardization exists for measuring and analyzing shape, appearance and motion features of cells. Existing software packages (e.g., CellProfiler^34^, TISmorph^35^) define their own set of phenotypic features, concatenating independently computed image features without systematic design rationale and establishment of effectiveness on limited datasets and phenotypes. The comprehensiveness and general applicability has not been systematically verified. Moreover, being developed primarily for analyzing fixed multiplexed images, these software packages provide limited assessment of motion features. Many studies simply compute image features ad-hoc, on a per experiment basis, targeted at hypothesis verification^36–39^. Temporal analysis of computed features presents further unique challenges. While methods are developed to improve identification and tracking of individual cells, there is limited consideration of how to integrate multidimensional SAM features extracted per cell instance with tracking information for the ultimate objective of assessing how perturbation conditions affects the control^18^. Most biological imaging analyses heuristically average the features of tracked objects temporally and/or ensemble-wise within a condition for comparison or resort to training an AI model^39–42^. These practices do not account for inherent heterogeneity of the biological system, including the impact of potential artifacts and temporal changes. Heterogeneity is a hallmark of cells in complex tissue. True biological variation exists between individual cells, and within cells of the same type, in the same microenvironment, and is important for proper function. Indeed, a characteristic signature of diseased tissue is a shift in the observed cell heterogeneity^31,43–45^. Recently, the development of deep learning AI has driven approaches to automatically ‘learn’ the most relevant imaging features directly from input data without explicit rational design^46–49^. There is, however, no large publicly available data repository for SAM method development. While there are now large multiplexed fixed imaging datasets^50,51^, these lack further comprehensive phenotypic annotation to be used like gold-standard computer vision datasets such as ImageNet^52^, or COCO^53^ to benchmark progress and drive systematic AI development. Moreover, due to its specialist nature, we cannot reliably crowdsource annotations as practiced in computer vision^54^. Training any foundational AI to generate universally applicable SAM features would therefore involve a minimum of constructing an exhaustive well-annotated library^55,56^ (e.g., taking into consideration all possible conditions, cell types, treatment conditions and imaging modalities). Meeting this single requirement involves substantial experimental and infrastructure investment^57^, let alone the fact that any trained foundational AI model requires continual retraining to account for changes in experimental set-up^56,58–60^.

To overcome the aforementioned challenges, we take inspiration from scRNA-seq. That is, to harness the power of label-free bright-field live cell time-lapse imaging, we must first develop: (1) a “standard” image-based “phenome” with the same premise as reference genomes: a set of shape, appearance, and motion features that can be universally applied to ‘image-sequence’ the instantaneous state of any object, or cell, acquired by any imaging modality; and (2) a companion standardized analytical framework, SAM phenotype observation tool (SPOT), to universally analyze all computed SAM phenomes in a dataset. The primary aim of the framework being to discover distinct dynamic phenotypic states and the identification of those conditions that cause bona fide phenotypic responses, all while taking into account the temporal evolution of phenotypic heterogeneity within individual conditions. Herein, we describe our development of a suitable 2185-D shape, appearance and motion (SAM) phenome and SPOT “phenome-analyzer”. We validate the universality and effectiveness of unbiased SPOT analysis of dynamic imaging phenotypes on small and large publicly available computer vision and 2D live-cell imaging datasets. Importantly, we demonstrate that SAM-SPOT analysis outperformed features generated by pretrained or training deep learning AI models on both fixed, and live imaging data, in both classifier accuracy and interpretability. Moreover, in a companion paper^61^ we also show that SPOT can characterize subtle and complex 3D organoid dynamics from analyzing only their 2D projection videos.

## Results

### Constructing a phenome comprising 2185 SAM features to characterize the instantaneous state of a dynamic object

**Fig. 1:**
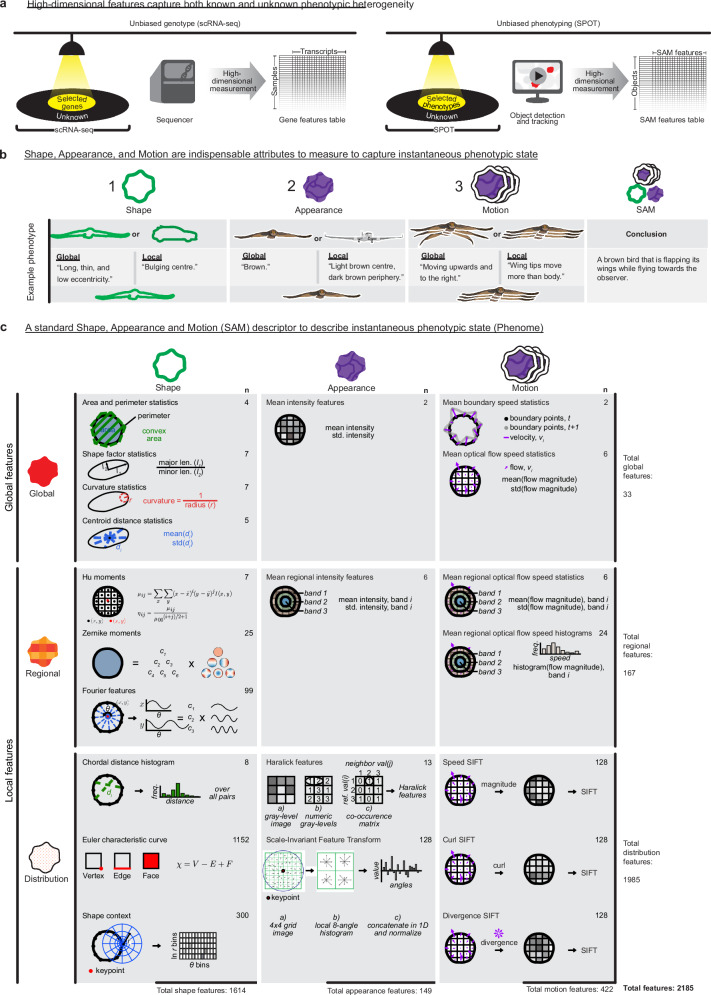
Comprehensive shape, appearance and motion (SAM) characterization enables analysis of phenotypic heterogeneity in timelapse imaging. **a** Measurement of a standardized high-dimensional library of gene transcripts in single-cell RNA sequencing (scRNA-seq) enables unbiased characterization of the full transcriptome: both that which is known or selected a priori (yellow circle) and unknown (gray ring). Similarly, measurement of a standardized high-dimensional set of SAM features could characterize imaging phenotypes that are known or selected a priori (yellow circle) and unknown (gray ring). **b** Example illustrating how shape, appearance and motion cues provide complementary information and are the minimum three essential concepts necessary to describe the instantaneous state of a dynamic object fully. **c** Illustration and categorization of the standardized SAM feature set (phenome) used in this study with respect to shape, appearance, or motion (columns) and the spatial scale of object detail they measure (rows). Global refers to the whole object, regional refers to a part of the object, and distribution measures the spatial scale of the object at the level of individual constituent pixels. See Supplementary Data 1 for the definition of individual features. The number of feature dimensions is given assuming an object contour of 200 equidistantly spaced points.

An imaging “phenome” to detect and compare known, unknown, and complex phenotypes in high-content timelapse videos should measure object-agnostic, phenotype-agnostic, image features characterizing an object at multiple spatial scales: global, regional and pixelwise in the current timepoint and its change to the next timepoint. These features should be easy to compute, with minimal assumptions, and discriminate between phenotypic variations in hundreds of thousands of individual moving objects across all timepoints, regardless of the imaged object or the frequency and length of the acquisition (Fig. 1a).

To construct such a phenome, we considered shape, appearance and motion (SAM) as constituting a minimal set of image-based object properties to explicitly characterize. Shape refers to an object’s external outline; Appearance refers to the pattern of image intensity values, the texture within the object’s internal area; and Motion refers to the object movement between time *t* and *t* + 1 including any translational shift in the object centroid and pixel-wise non-rigid movements measured by optical flow^62^. While there is inevitably feature overlap between the three properties, crucially, they individually provide complementary and indispensable information to fingerprint the object and its instantaneous action state. Figure 1b depicts how a combination of global and local SAM features provides information to facilitate discrimination of a flying bird from other possible objects. Shape features help identify the possible object type. The bird outline is distinctive from that of the car, but indistinguishable from the outline of the airplane. Appearance features are then necessary for finer-grained discrimination of the bird from the airplane. Lastly, motion is required to identify the dynamic state of the bird: whether the bird is actively flying, flapping its wings or gliding with an immobile wing posture. Specifically, to ensure a maximally applicable SAM phenome for real data, we developed our SAM phenome upon four design principles (Fig. 1c). First, SAM properties should be measured at multiple spatial scales; global (whole object), local-regional (parts of whole object) and local-(pixel-level) distribution. Second, at each scale, a broad set of SAM features should be measured to capture all potential pattern variations, and not limited to only identifying object phenotypes within a particular analyzed dataset. This includes measuring redundant features, which characterize the same property but are computed using different methodologies with different principles. Redundancy both builds robustness to inaccuracies in segmentation and tracking, and provides complementary information. For example, the area measurement is more erroneous for a partially segmented object than the convex area measurement. Yet, using only the convex area underestimates differences in actual area when two shapes are accurately segmented but they are not actually convex-shaped. Third, features are computed to be invariant to object rotation by design using permutation-invariant operations^63^ (Fig. 1c, Supplementary Data 1). The shape-appearance of an object remains the same under rotation and should therefore have the same SA phenome. However, two objects can share the same shape and appearance but move differently, resulting in different combined SAM phenomes. Only when two objects have both the same shape-appearance and the same motion pattern do they have identical SAM phenomes. Reflection invariance^48^ is not enforced as these may reflect real differences in the biological process or inform a systematic difference in acquisition (e.g., microscope camera flipped). Reflections of an object can therefore have different SAM phenomes. Last, to enable computation of the phenome for objects segmented from videos of any duration and acquisition frequency, we consider only first-order motion features; those computable using a pair of consecutive frames, between time *t* and *t* + 1. Higher-order motion properties such as acceleration are implicitly captured in the tracking information over multiple frames.

Briefly, for Shape, we compiled 1614 features, including 23 global (area and perimeter, shape factor, curvature and centroid distance statistics), 131 regional (Fourier features^64,65^, Hu^66^, and Zernike^64,67^ moments) and 1460 distribution features (chordal distance histogram^35^, Euler characteristic curve^68,69^, shape context^70^). For Appearance, we compiled 149 features, including 2 global (mean intensity), 6 regional (regional intensity) and 141 distribution features (Haralick^71^, Scale-Invariant Feature Transform (SIFT)^72,73^). For Motion, we generated 442 features, including 8 global (mean boundary speed, mean optical flow speed statistics), 30 regional (mean regional optical flow speed statistics and histograms) and 384 distribution features (Speed, Curl and Div SIFT). The concatenation of all Shape, Appearance and Motion features (1614 + 149 + 442 = 2185) form our final 2185-D SAM phenome (Fig. 1c and Supplementary Data 1). The rationale for including each feature is provided in Methods. The total number of features is high enough to ensure robustness and discrimination but not so high as to suffer from the curse of dimensionality which determines the minimum dataset size (see “Discussion”).

To ensure our SAM phenome is computed correctly in code in our analytical workflow, SPOT, the following additional processing was also implemented (“Methods”). We binary infill each segmented object to extract a single largest contour and represent it as a 200-point polygon, which accurately captures the external shape and minimizes storage memory (“Methods”, Supplementary Fig. 1a, b). We rasterize the contour representation to compute any SAM features (Fig. 1c, Supplementary Data 1) requiring a binary shape image. Lastly, we apply principal component analysis to pre-align individual object contours with respect to their major length axis, as additional insurance for computing rotation-invariant shape features^48^.

We tested the empirical invariance properties of our SAM phenome on synthetic manipulations of digits from the handwriting dataset, MNIST^74^ using principal component analysis (PCA) (“Methods”). The SA phenome was not numerically identical across rotations and polar orientation displaced versions of the same digit due to the inexactness of manipulating discrete pixel images. Also, since we did not design the SAM phenome to be reflection-invariant, flipped versions of the same image were expected to be different (Supplementary Fig. 1c–e). However, overall, the differences in SAM phenomes within a digit were smaller than the unique differences between the digits 0, 1, 2, and PCA found three unique digit clusters (Supplementary Fig. 1d, e). Across all digits, 0–9, small variations do cause the PCA to split 0 ± 90° rotations and 180° ± 90°rotations of certain digits into two distinct clusters, when these may share greater similarity with rotated versions of another such digit (Supplementary Fig. 1f). Notable example digit groupings are 2 with 5, 3 with 7, 6, 8 with 9. We consider this rational behavior, and represents a phenomic landscape, a semantic organization of phenotypically similar datapoints in a two-dimensional space. If more ideal invariance is desired, at the expense of slower computation, we can readily compute a mean SA phenome for each input—averaging the individual SA phenomes of data transformed versions of the input (augment SAM features, Supplementary Fig. 1g, h). Supplementary Fig. 1i, j show that optical flow and our current motion features encode the same rotational motion as a local displacement field unique to each digit, but invariant to a digit’s orientation. Consequently, the motion features-alone, M-phenome PCA discriminated the digits 0, 1, 2. Combined with SA features, the SAM-phenome PCA distinguished both unique digits and separated the difference between +/−15° and +/−45° rotational motion patterns, irrespective of digit orientation and motion differences can be further enhanced through SPOT’s temporal filtering feature preprocessing (see below) and concatenating with average SA phenomes from data transformed inputs for the digits 0, 1, 2 (Supplementary Fig. 1k–m), and also for all digits 0–9 (Supplementary Fig. 1n, o). Combined, these synthetic experiments verify the practical invariance properties of our SAM phenome to informatively discriminate patterns in data. These experiments also show that while our choice of binary infilling destroys the shape topology of the digits 0, 6, 8, and 9, their discrimination is nevertheless accurate—enabled by our appearance and motion features that do account for topological differences. Henceforth, in our analyses in this paper, and our organoids-specific companion paper^61^, we prioritized speed and did not use data transformations to compute mean SAM phenomes.

### Development of SPOT

**Fig. 2:**
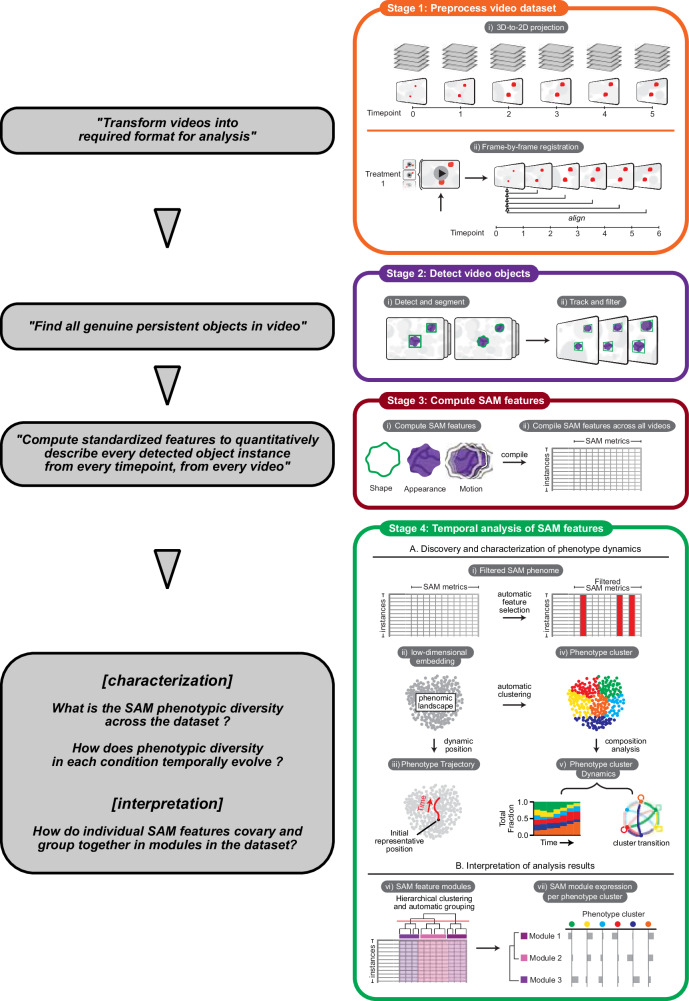
Overview and rationale of the SPOT analysis framework. The four stages in the SPOT workflow with design rationale (left) and illustration of the steps (right) within stage 1: preprocess video datasets (orange box), stage 2: detect video objects, stage 3: compute the SAM phenome for each object instance (brown box) and stage 4: temporal analyses of compiled SAM features (green box).

To jointly analyze the computed SAM phenomes of all segmented object instances across time from video datasets in an unsupervised, data-relevant and high-throughput, streamlined manner with minimal prior assumptions, we developed the SAM Phenotype Observation Tool (SPOT). The four main stages of the SPOT workflow are summarized in Fig. 2 (details in “Methods”). Stage 1 comprises two preprocessing steps: (i) 3D-to-2D projection to convert individual video frames acquired as a 3D z-stack to 2D projection images, and (ii) frame-by-frame registration, to remove any translation motion artifacts including drift due to microscope stage movements. Stage 2 comprises (i) detection and segmentation of every object-of-interest at every timepoint, followed by (ii) tracking and filtering to retain only “genuine” detected objects that change behavior over time (i.e., those that were tracked consistently for a minimum number of consecutive frames). Stage 3 is SAM features computation. For all identified and segmented objects at each timepoint (an object instance), SPOT (i) computes the SAM phenome and (ii) compiles the SAM phenomes for all objects across all videos in the dataset into a single table for combined analysis. Stage 4 is the temporal analysis of SAM phenomes, and comprises two parts: part A, the discovery and characterization of phenotype dynamics, and part B, identification of modules of covarying individual SAM features to aid interpretation. For part A: SPOT uses dimensionality reduction similar to scRNA-seq workflows to analyze the compiled SAM phenomes (treating each SAM feature as a genetic transcript equivalent) and to map and quantify the temporal evolution of phenotypic heterogeneity. Specifically, SPOT: (i) filters SAM phenomes to remove noisy and uninformative features, thereby retaining only the subset of SAM features in the phenome most relevant for the experiment. Per feature normalization of retained features by standard scaling is applied, followed by power transformation to make each feature Gaussian-like. This normalization enables each feature to be treated independently with equal weight. We then perform regularized linear regression with time (temporal feature filtering), to remove features associated with systematic experimental errors and imaging artifacts in the dataset that should not vary with time; (ii) constructs the SAM phenomic landscape, applying dimensionality reduction to the filtered and normalized SAM phenomes to generate a reduced 2D coordinate mapping of the spatiotemporal phenomic variation across all object instances (represented by individual points). This landscape colocalizes object instances with similar SAM features from any timepoint and any condition in 2D, which is critical for enabling simplified downstream analysis; namely, (iii) constructing SAM temporal phenotype trajectories, a rationally-aggregated population-level summary of temporal evolution of phenotypic diversity to compare and cluster conditions, and (iv) finding SAM phenotypic clusters to discretely model the phenomic landscape for granular characterization of phenotypic heterogeneity dynamics by the (v) monitoring of phenotype cluster dynamics: cluster composition over time and the transition probability between clusters. To interpret the SPOT outputs in part A, in part B, SPOT vi) automatically finds SAM modules: groups of individual SAM features that exhibit the same covariation in the dataset, akin to grouping transcripts by biological pathways in RNAseq (Supplementary Fig. 2a–c). Individual SAM modules can be interpreted by their top representative image exemplars in the dataset and the top contributing individual SAM features (“Methods”). Finally, (vii) the pattern of SAM module expression aids interpretation of phenotype cluster dynamics (Supplementary Fig. 2b, c).

In summary, the SPOT workflow is designed for high-throughput temporal analysis of objects in videos, enabling the clustering of conditions by phenotypic impact, explicitly accounting for the phenotypic heterogeneity arising from real biological processes and processing artifacts. Treating every temporal instance of an object as an independent observation, SPOT amplifies each object by the number of temporal instances, increasing the ability of analyses to detect under-represented and rare phenotypes. Crucially, this amplification allows SPOT to use dimensionality reduction to map and visualize the full phenotypic variation across space in a 2D coordinate space (the SAM phenomic landscape) in a manner that preserves the global and local SAM similarity between objects, even for small datasets and limited object numbers, for streamlined temporal analysis. Specifically, we use a 2D landscape to construct two simple, universally applicable analytics (population phenotype trajectories and phenotype cluster dynamics) suitable for high-throughput, high-content live-cell imaging screens.

### The SAM phenome comprehensively captures shape and appearance heterogeneity in computer vision datasets with ground truth

The effectiveness of the SAM phenome and SPOT was tested on two publicly available computer vision datasets with ground truth and benchmarked against CellProfiler^75^—perhaps the most popular software for measuring shape and appearance features for cell biological applications, and TISMorph^35^. To assess the intrinsic information provided by and verify the necessity of all features comprising the phenome, all experiments were conducted without feature selection, applying only standard scaling and a power transform to normalize the features (“Methods”). Here, SAM phenome refers to the complete ensemble of 2185 SAM features in Fig. 1c and “feature set” to a subset of SAM features in the phenome: shape (SAM-S), appearance (SAM-A) and motion (SAM-M). Dimension (n) refers to the number of SAM features used in an analysis.

**Fig. 3:**
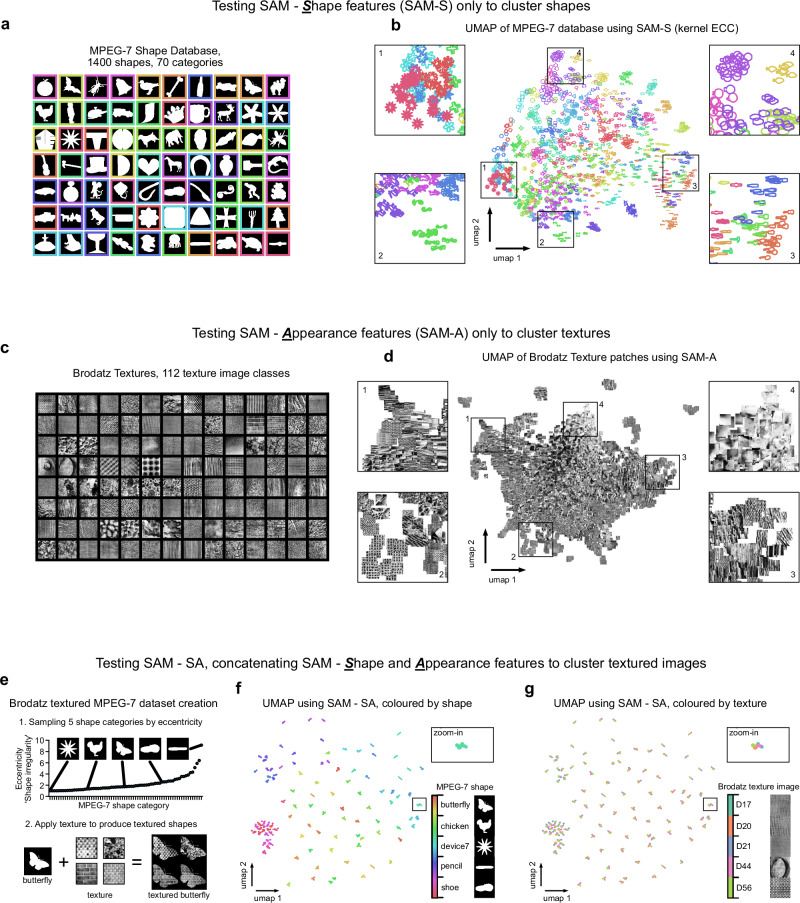
Standardized shape, appearance and motion (SAM) features can comprehensively map phenotypic diversity in computer vision datasets. **a** Exemplar binary shape images from the MPEG-7 shape computer vision database (one image per shape category depicted), used to test the informativeness of SAM-S, the shape only feature set. **b** Shape phenomic landscape constructed by applying 2D-UMAP to the full SAM-S (Shape (kernel ECC)) features. Each point is an individual shape image in the MPEG-7 dataset colored by its shape category. Similar shapes colocalize to the same local region of the landscape as shown by zoomed-in images 1–4. **c** Visual panel summary of the Normalized Brodatz computer vision texture images, used to derive a texture dataset to test the informativeness of SAM-A, the appearance only feature set. **d** Appearance phenomic landscape constructed by applying 2D-UMAP to the full SAM-A features. Each shape is a 64 × 64 image patch cropped from the original 640 × 640 112 Normalized Brodatz texture image. Images cropped from the same original Brodatz texture image and with similar textures colocalize to the same local region of the landscape as shown by zoomed-in images 1–4. **e** Five representative MPEG-7 shape classes selected according to eccentricity, defined as the ratio of the length of the longest axis to the shortest axis of an ellipse fitted to the shape (1, top), used to create textured shapes by combining MPEG-7 binary masks with Normalized Brodatz textures for testing SAM-SA (concatenation of shape and appearance features). The textured shape images were generated by multiplication of images of the same size, one MPEG-7 binary image and one grayscale image crop from a Normalized Brodatz texture image (2, bottom). **f** Shape-appearance phenomic landscape constructed by applying 2D-UMAP to the concatenated full SAM-S (Shape (kernel ECC)) and SAM-A feature sets of the textured shapes created from five selected MPEG-7 shape categories (**e**) and five randomly selected Normalized Brodatz texture images. Each point is a 128 × 128 image colored by **f** its source MPEG-7 shape category and **g** source Brodatz texture.

The MPEG-7 database^76^ is an established computer vision benchmark shape classification dataset containing 1400 shapes equally sampled from 70 different categories (Fig. 3a). Using this dataset, we tested the ability of the SAM-S feature set to correctly cluster objects into their origin shape categories using unsupervised k-means clustering^77^. Clustering performance was measured based on adjusted mutual information (AMI) and adjusted rand index (ARI) (these scores range between 0 and 1, with 1 being the best performing score) (“Methods”). We tested the full SAM-S as well as subgroupings of individual SAM-S features (Supplementary Data 2), and applied dimensionality reduction using kernel maps to preprocess Euler Characteristic Curve (ECC) shape features, which has been reported to give better performance^68^: SAM-S (kernel ECC) and SAM-S (ECC) indicate the full SAM-S with and without dimensionality reduction, respectively.

2D UMAP analysis applied to SAM-S (kernel ECC) displayed the MPEG-7 shape variation in an ordered manner, from round and symmetrical (left-side of UMAP) to elongated (right-side of UMAP), while maintaining clustering amongst individual shape categories (Fig. 3b). 2D UMAP analysis applied to CellProfiler features similarly ordered the MPEG-7 shapes but exhibited a reduced ability to cluster similar-looking shapes, resulting in two distinct point-clouds instead of one (Supplementary Fig. 3a, b). Like CellProfiler, TISMorph also showed reduced clustering capability (Supplementary Fig. 3i). Additionally, SAM-S (kernel ECC) (AMI = 0.71, ARI = 0.52) outperformed subgroupings of SAM-S features and performed better than CellProfiler (AMI = 0.68, ARI = 0.47), and TISMorph (AMI = 0.61, ARI = 0.38) computed shape features (Supplementary Data 2).

The Normalized Brodatz database is an established computer vision benchmark texture (appearance) classification dataset, containing 112 unique texture images^78,79^. Treating these 112 images as independent texture classes, we applied cropping and rotation to construct an augmented dataset of 22,400 image patches. We then tested the ability of the SAM-A set (Fig. 3c, “Methods”) to correctly cluster patches into their appearance category using unsupervised k-means clustering^77^. As for the shape analysis, the full SAM-A (AMI = 0.47, ARI = 0.15) outperformed subgroupings of SAM-A features, and TISMorph (AMI = 0.45, ARI = 0.14) in clustering and was competitive with CellProfiler computed appearance features (AMI = 0.49, ARI = 0.14) (Supplementary Data 2). Notably, despite the similar clustering performance, SAM-A organized image patches with emphasis on textural pattern similarity in 2D UMAP space, whereas CellProfiler (Supplementary Fig. 3c,d) and TISMorph (Supplementary Fig. 3j) emphasized similarities in global brightness.

To test the ability to jointly characterize shape and appearance variation, we combined 5 Brodatz textures and 5 MPEG-7 shape classes. For each Brodatz class, we extracted 20 random crops and applied them to each of the 20 unique shape images in each MPEG-7 class, to construct a synthetic dataset of 10,000 images with joint shape and appearance variations (Fig. 3e, “Methods”). Applying SPOT to compute all combined shape and appearance features (SAM-SA) and using 2D UMAP, we observed that unique shape images were separated as individual tightly grouped colored point-clouds (Fig. 3e). Each point-cloud was further subdivided into 5 smaller point-clouds, each corresponding to a Brodatz texture (Fig. 3f and zoom-ins). Thus, SAM-SA could reverse-engineer the data generation process. Globally, there is also a broad separation of the 5 primary shape categories, demonstrating SAM-SA finds object shape as the most important global difference. In contrast, UMAP applied to CellProfiler SA-features clusters point-clouds at the coarse level of MPEG-7 shape and Brodatz texture classes but cannot resolve unique shape images (Supplementary Fig. 3g, h and zoom-ins). Meanwhile, TISMorph-SA features could not resolve individual texture variations of a single shape image (Supplementary Fig. 3k, l). We validated this observed hierarchy of information by generating UMAPs with only the shape and appearance features (Supplementary Fig. 3m–x). We further tested the ability of each feature set to train a linear support vector machine with different training data sizes to classify (i) shape, (ii) appearance, and (iii) shape-appearance (Supplementary Data 2). On aggregate, SAM-SA outperformed all others in classification. Only when the training split was <5% was SAM-SA penalized for having a higher number of features than TISMorph and Cellprofiler. Altogether these benchmarking analyses of synthetic datasets confirm that SPOT’s SAM phenome is more discriminative than TISMorph and CellProfiler in capturing joint shape and appearance variations.

### Applying SAM phenome and SPOT to characterize shape, appearance and motion of objects in YouTube videos with ground truth

**Fig. 4:**
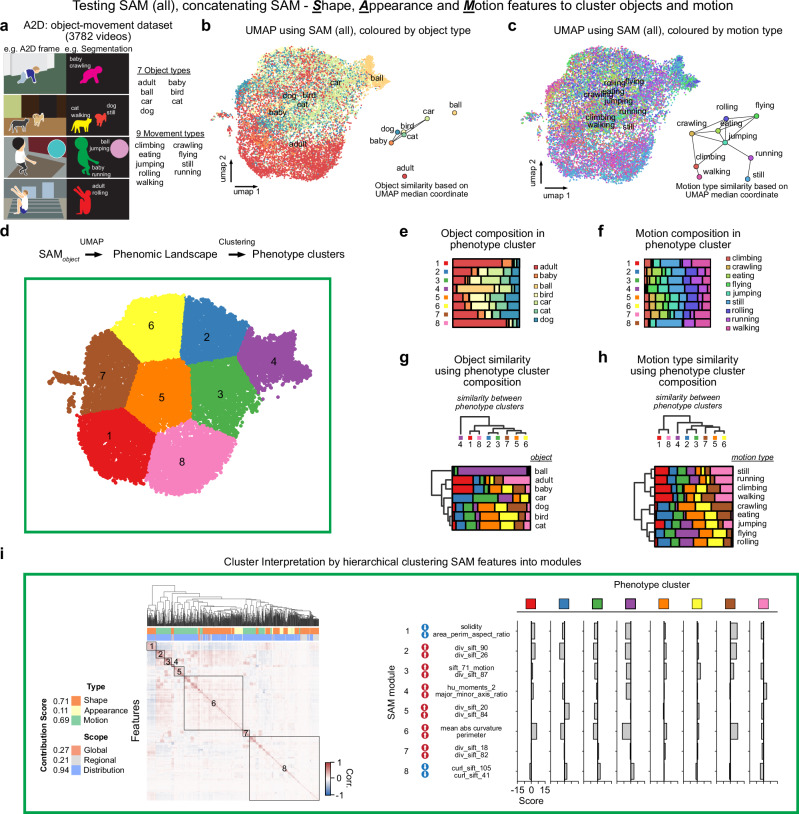
Testing shape, appearance and motion features to cluster objects and motion in the A2D YouTube dataset. **a** Schematic examples of the object-movement pair annotation in the YouTube-derived A2D dataset used to test the full SAM feature set on videos representing real-life conditions. SAM phenomic landscape of object-motion constructed by applying 2D-UMAP to the full SAM feature set concatenating SAM-S (kernel ECC), SAM-A and SAM-M feature sets colored by **b** object type or **c** movement class for each A2D annotated object in the test split. Insets: similarity graph between object and motion classes, respectively, based on the pairwise distance between median 2D-UMAP coordinates. **d** SAM phenomic landscape constructed by applying UMAP to the SAM phenome, followed by identification of phenotype clusters by k-means clustering of 2D-UMAP coordinates using the elbow method. **e** Object and **f** motion composition according to phenotype cluster. **g** Similarity between object types based on hierarchical clustering of their phenotype cluster composition (bottom). Similarity between phenotype clusters based on hierarchical clustering of their object type composition (top). **h** Similarity between movement type and phenotype clusters based on hierarchical clustering, same as for (**g**). **i** Contribution score of shape, appearance and motion, and global, regional and distributional features in explaining the dataset variance defined as the absolute value of the first principal component (“Methods”) (left). Automated hierarchical clustering of the covariation between SAM features to identify principal SAM feature modules (middle, modules outlined and numbered along the diagonal). The expression of each SAM module (labeled as ‘score’ in barplots, Methods) in each phenotype cluster (right). The top driving SAM features for each module and whether they are shape (S), appearance (A) or motion (M) based are stated, with arrows indicating whether the feature is enriched (up arrow) or depleted (down arrow).

We used our SAM phenome to characterize combined variations in shape, appearance and motion of individual objects in the A2D dataset^80^, comprising 3782 YouTube videos of unconstrained ‘real-life’ scenarios. The videos depict seven classes of moving objects (adult, baby, bird, cat, dog, ball and car) performing nine different movements: still (no movement), climbing, crawling, eating, flying, jumping, rolling, running, and walking (Fig. 4a). A single movement class can be performed by different objects, but no object can perform all eight movements, totaling 43 unique object-movement pairings. As expected, SAM phenome significantly outperformed SAM-S, SAM-A and SAM-M features alone in clustering and in training supervised classifiers of object, movement and object-movement pairings (Supplementary Data 2, scores ranging from 0 to 1, with 1 being the highest score).

We next used SPOT analysis to interrogate the phenotypic heterogeneity within the dataset. As intended by design, SPOT co-clustered object-movement (actor-action) pairings in the UMAP phenomic landscape. The ‘ball’ object maps to the top right of the UMAP, colocalized with the ‘flying’ movement whereas the ‘adult’ object maps to the bottom of the UMAP where it is associated with running, jumping, walking and still—actions most frequently paired with ‘adult’ within this dataset (Fig. 4b, c). Overall, the UMAP spatially arranged object and movement types semantically such that we construct meaningful similarity graphs for object (Fig. 4b) and movement type (Fig. 4c), and joint object-movement pairings (Supplementary Fig. 4a), using only 2D median UMAP coordinates (“Methods”). This semantic organization was maintained when we varied the choice of dimensionality reduction methods, demonstrating that our constructed SPOT phenome is indeed comprehensive and discriminative (Supplementary Fig. 4b). Herein, we use UMAP in SPOT as the default dimensionality reduction method due to its grounding in mathematical theory^81^, scalable computation, robust open-source code implementation and proven performance in literature. SPOT (Stage 4) partitioned the 2D UMAP landscape into 8 phenotype clusters, with cluster number automatically inferred by the elbow method (Fig. 4d). These clusters should be considered “prototypical SAM phenotypes”. Consequently, we do not expect each cluster to correspond exclusively to any one single object or movement type (Fig. 4d). However, one object or movement type may be stereotyped, having such distinct phenotypic characteristics that they primarily dominate a cluster or several clusters. For example, adults predominate in clusters 1, 8 (Fig. 4e); and these clusters also have near-identical motion composition patterns of being still or walking (Fig. 4f). Likewise, the predominant object in cluster 4 is ball, followed by car and bird (Fig. 4e), and this cluster expectedly has the largest proportion of flying objects (Fig. 4f). Hierarchical clustering of objects correctly identify ball as most dissimilar to other objects, human adult and baby as a clade, and distinguishes car from animals: dog, bird and cat (Fig. 4g). Movement type is less distinct, due to the diverse shape and appearance of objects conducting the same movement. Nevertheless, the hierarchical clustering is meaningful and finds a grouping of aerial-associated activities: jumping, flying and rolling; vertical activities: walking, climbing, running and still; and a miscellaneous grouping of crawling and eating (Fig. 4h). We can similarly group phenotype clusters by similarity of object and movement type composition (Fig. 4g, h).

To interpret individual phenotype clusters with respect to SAM features, we performed SPOT Stage 4B (Fig. 4i, “Methods”). Given that SAM phenome annotates each feature by concept (shape, appearance and motion) and by spatial scale (global, regional, distribution), from a grouping of features, SPOT can compute a score of the group contribution to the total phenomic variation (“Methods”, Supplementary Fig. 2b, c). In the A2D dataset, we find the primary sources of variation are shape and motion, at (pixelwise) distribution scale with contribution scores of 0.71, 0.69, and 0.94, respectively (Fig. 4i, left). SPOT automatically identified 8 distinct SAM modules, mutually exclusive groupings of correlated SAM features by hierarchical clustering, and their most relevant image exemplars and individual SAM features. We computed the module expression (the contribution score of each module in each phenotype cluster) to interpret clusters. Module 6, primarily described by mean absolute curvature can be interpreted as measuring the shape ‘complexity’ of an object’s contour. The higher its expression, the more complex the object shape. Accordingly, cluster 4, primarily associated with ‘ball’, a round object, has low module 6 expression. In contrast, cluster 7, comprising a diverse mixture of adult, dog, bird, baby (Fig. 4e) and movement types (Fig. 4f) has the highest module 6 expression. These computer vision results demonstrate that SPOT’s SAM phenome construction is comprehensive and applicable to diverse datasets, such that SPOT can automatically find the most relevant subset of shape, appearance, and motion features for analysis.

### SPOT comprehensively profiles dynamic phenotypic heterogeneity in single cell videos

**Fig. 5:**
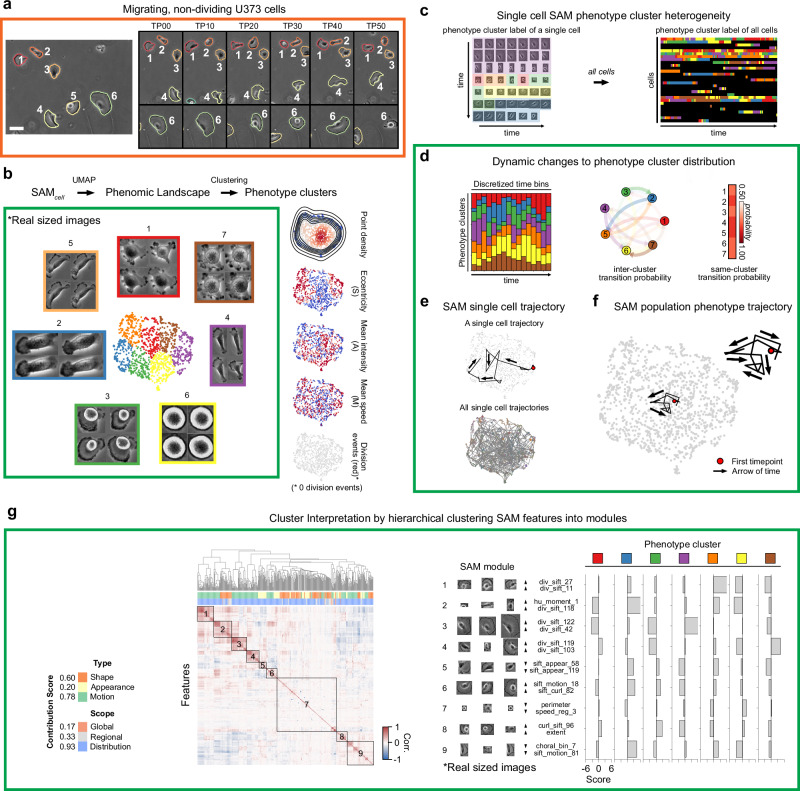
SPOT characterizes phenotypic heterogeneity of single cell migration. **a** Field-of-view at t = 0 of glioblastoma-astrocytoma U373 cells with uniquely colored boundaries for each fully visible cell (left), and snapshots of cells at select timepoints (right). Cells did not divide over the video duration. One timepoint is a video frame (15 min). Scalebars: 50 µm. **b** SAM phenomic landscape constructed as in Fig. 4d. 2×2 exemplar image panels of the principal phenotypes in each color-coded cluster (left). Local point density of mapped cell instances, represented by points colored to indicate low-to-high (blue-to-red) measured values in global SAM features of shape (eccentricity), appearance (intensity), and motion (speed), (right, top-to-bottom panels). Red instances indicate the first timepoint after cell division (right, fifth panel). As U373 cells did not divide, all points are gray. **c** Mapping a single cell tracked over time (left) and all continuously tracked cells (right) into the SAM phenomic landscape of (**b**) and coloring each temporal instance by the corresponding phenotype cluster. **d** Stacked barplot showing the relative frequency of each phenotype cluster over discrete time bins (left). Graph showing the next timepoint relative transition probability to a different phenotype cluster (inter-cluster) (center) and heatmap of the transition probability to the same phenotype cluster (same-cluster) (right). Arrows are colored by the source cluster. The more transparent the arrow, the smaller the probability of the transition. **e** SAM phenotype trajectory of a single U373 cell (top) and of all cells (bottom), and **f** the single population-level SAM phenotype trajectory with starting timepoint colored red and black arrow showing time directionality. **g** Contribution score of shape, appearance, motion, global, regional and distributional features in explaining the dataset variance (left); principal SAM feature modules (middle, modules outlined and numbered along the diagonal); and the expression of each SAM module (labeled as ‘score’ in barplots) in each phenotype cluster (right) as in Fig. 4i.

To test the applicability of SPOT workflow (Fig. 2) for biological image analysis, we applied SPOT to detect and compare cell behaviors in live-cell imaging, using two Cell Tracking Challenge^82^ datasets (with reference segmentation and single-cell tracks provided). The first glioblastoma-astrocytoma U373 cell dataset records cell migration without cell division (Fig. 5a, Suppl. Movie 1). Based on the provided segmentations and single-cell tracks of U373 cells, SPOT found seven phenotype clusters (Fig. 5b, left, “Methods”). Consistent with the expected cell morphodynamics in video, cells in cluster 1 (red) and cluster 7 (brown) have a shape composed of a central circular ‘core’, surrounded by individual finger-like protrusions; cells in clusters 2 (blue), 4 (purple) and 5 (orange) have elongated core shapes with asymmetric lamellipodium; and cells in clusters 3 (green) and 6 (yellow) have a single bright, round nucleus distinguished by the presence or absence of lamellipodia. To aid cluster interpretation, we also colored the phenomic landscape using selected SAM features (Fig. 5b, right). The local point density shows that most cell instances map to the center (Fig. 5b, right panel, first plot), covered by cells with phenotypic features identified in clusters 1, 7 (spherical) and 3, 6 (bright and round nuclei), while the left and right extremities (cluster 2, 4, and 5, respectively) comprise eccentric, elongated cell shapes (Fig. 5b, right panel, second plot). The mean intensity and mean speed exhibit no clustering and no cell division events were detected, meaning these are non-discriminative features in this dataset, as expected (Fig. 5b, right panel, third to fifth plots).

The phenotype clusters are SAM analogues of molecular clonotypes^83,84^ from scRNA-seq, decomposing the full phenotypic heterogeneity. To visualize and quantify the temporal evolution of heterogeneity, SPOT assesses the phenotype cluster composition of cell populations (Fig. 5c) within discrete temporal bins (stacked barplots), and computes the relative probability of transitioning to a different phenotype cluster or staying in the same phenotype cluster in the next timepoint using the single cell tracking information (Fig. 5d, “Methods”). All U373 phenotype clusters expand and contract, most visibly represented by cluster 6 (yellow), and suggests recurrent transitions among clusters (Fig. 5d). Cluster 6 absorbs cells predominantly from clusters 4 (purple) and 7 (brown), and vice versa, albeit with lower probability in reverse (Fig. 5d, middle).

To cluster conditions without computing phenotype clusters, suitable for high-throughput, high-content screens, SPOT must derive a single summary ‘signature’ per condition that accounts simultaneously for heterogenous cell behavior and temporal evolution. SPOT does this by adapting the concept of pseudotime trajectories^37,85^ from scRNA-seq analyses to develop the SAM population phenotype trajectory. For a single tracked cell, we define its phenotype trajectory by chronologically linking the UMAP coordinates of all its individual temporal instances (Fig. 5e, top). The entire set of single-cell phenotype trajectories captures the dynamics of all 29 tracked cells (a total of 1189 cell instances from U373 videos) but is significantly heterogeneous (Fig. 5e, bottom). Crucially, individual trajectories are of different durations, having different start and end times. Generating a consensus trajectory from individual trajectories requires not only complex and time-consuming alignment but may also destroy the heterogeneity pattern. Consequently, SPOT constructs its population trajectory by subdividing time into discrete temporal bins, deriving an ‘average’ UMAP coordinate reflective of the phenotypic diversity of cells in each bin, and linking the coordinates chronologically (“Methods”). The resultant cyclic trajectory of U373 cells: from cluster 1 (red) towards 5 (orange), towards 2 (blue), towards 3 (green), towards 6 (yellow), and back to 1 (Fig. 5f) reflects the periodic expansion and contraction of clusters in stacked barplots and captures the long-time fate of the short-time dynamics between successive timepoints.

The second dataset computer-simulates fluorescently labeled acute promyelocytic leukemia HL60 cells undergoing primarily cell division in-place with negligible cell migration (Supplementary Fig. 5a, Supplementary Movie 1). SPOT generated a SAM phenomic landscape which identified six phenotype clusters (Supplementary Fig. 5b, left), characterizing cells undergoing or having just experienced cell division (clusters 1, 2, 4, 5: high eccentricity reflecting the elongated eccentric shapes during chromosome segregation) and those that have not divided (clusters 3, 6).

As HL60 cells divide, they lose fluorescence. Brighter and darker pixel intensity values therefore discriminate between clusters containing cells that have undergone division recently (Supplementary Fig. 5b, c). Consistent with this, clusters 1 and 3 diminish over time (Supplementary Fig. 5d, left). We also find two next timepoint inter-cluster transitions representing cell division at high (cluster 3 to 1) and low (cluster 6 to 2, cluster 6 to 4) fluorescence intensity, respectively (Supplementary Fig. 5d, middle). Meanwhile, cluster 5 remains relatively stable in size, as it appears to be transient, with a lower self-transition probability (Supplementary Fig. 5d, right). Again, individual single cell phenotype trajectories are heterogeneous and it is difficult to extract a consensus behavior pattern (Supplementary Fig. 5e). SPOT’s SAM population phenotype trajectory, however, clearly reveals the long-time behavior of 4 ‘loop-like’ directional changes (Supplementary Fig. 5f), indicating a total of four cell cycles in the dataset, corroborating the observed division pattern across all individually tracked cells (Supplementary Fig. 5c, right).

SPOT’s SAM module analysis quantifies our qualitative observation that U373 cells primarily migrate and change shape, whereas HL60 cells divide, changing their shape and appearance. For U373 cells, distribution-based shape, motion features were primary contributors of SAM phenotypic variation, with scores of 0.60, 0.78 and 0.93, respectively (Fig. 5g, left). For HL60 cells, distribution-based shape and appearance features contributed the most, with scores of 0.59 and 0.78, respectively (Supplementary Fig. 5g, left). Importantly, appearance (0.20 score) and motion (0.22 score) features were low in U373 and HL60, respectively. Accordingly, SPOT finds more motion-driven modules in U373 (primarily ‘div_sift’, ‘curl_sift’ or ‘sift_motion’ features) (Fig. 5g, right), and more appearance-driven modules in HL60 (‘mean_intensity’, ‘sum_entropy’, ‘diff_variance’, ‘ang_second_moment’, ‘sift_’ features) (Supplementary Fig. 5g, right).

Together, these results confirm that SPOT successfully detects and extracts informative and interpretable SAM features automatically for cellular imaging and can cluster cell instances based on their SAM phenomes. Moreover, using datasets of stereotypical cell migration and division, we validated that SPOT analysis (Fig. 2) generates comprehensive but concise readouts of dynamic phenotypic heterogeneity: capturing the long-time behavior with stacked barplots of phenotype cluster frequency and SAM population phenotype trajectory; and short-time (i.e., next timepoint) behavior with inter- and self-cluster transition probabilities.

### SA-phenome outperforms deep learning learnt features on 1.6 million fixed cell images

To demonstrate that our SAM phenome and SPOT is also discriminative and applicable to large biological datasets, we analyzed two publicly available datasets, one each representing fixed and live-cell imaging. We applied SPOT analysis with SAM phenome and deep learning learnt features to analyze one of the largest publicly available database of fixed cell imaging, LIVECell (Label-free In Vitro image Examples of Cells)^86^. LIVECell is a well-annotated large-scale dataset of over 1.6 million cells imaged using label-free phase contrast microscopy from 8 cell-lines chosen to represent a diverse set of cell morphologies, including small and round, large and flat, neuronal-like with long protrusions, and cultured under different densities^86^ (Supplementary Fig. 6a). The LIVECell dataset is provided as two data splits; a train-validation (val) split (1,011,452 cells) and a test split (415,518 cells) with annotated cell segmentation masks.

**Fig. 6:**
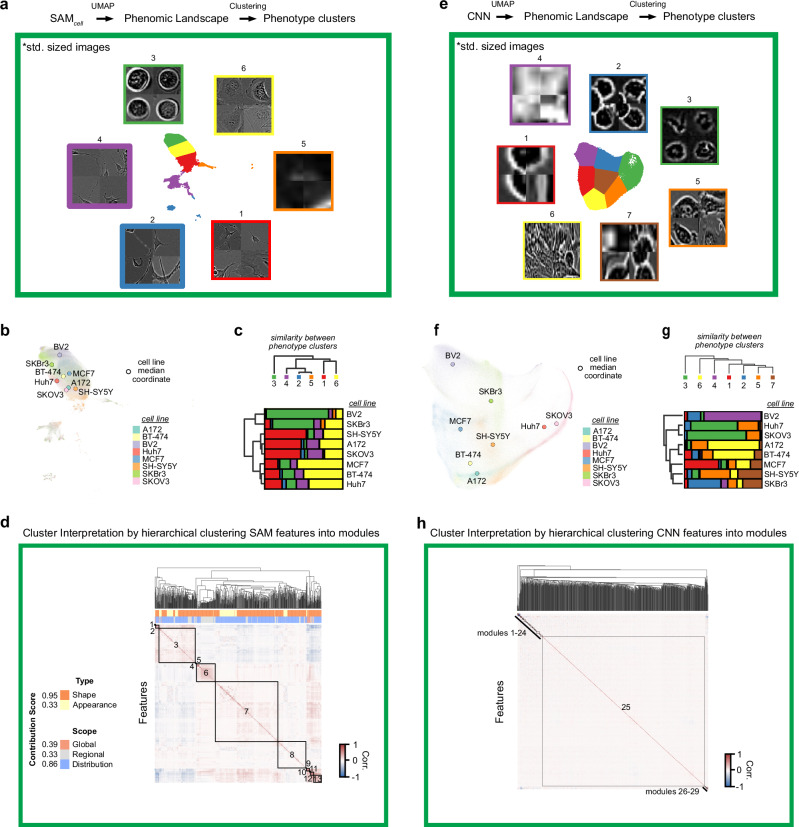
SPOT characterizes phenotypic heterogeneity of different cell types in the LIVECell dataset. **a** SAM phenomic landscape constructed by applying UMAP to the SAM phenome, followed by identification of phenotype clusters by k-means clustering of 2D-UMAP coordinates using the elbow method. 2×2 image panels show exemplars of the principal phenotypes in each color-coded cluster (“Methods”). **b** SAM phenomic landscape with each point (a cell), colored by its cell line. Colored circles show the median coordinate of each cell line. **c** Hierarchical clustering using average linkage of phenotype cluster based on occurrence across the 8 cell lines (top) and of the 8 cell lines based on their phenotype cluster composition (bottom). **d** Contribution score of shape, appearance and motion, and global, regional and distributional features in explaining the dataset variance defined as the absolute value of the first principal component. Automated hierarchical clustering of individual SAM features based on pairwise correlation between features to identify SAM modules (middle, modules outlined and numbered along the diagonal). **e** Phenomic landscape constructed by applying UMAP to CNN features. Cluster coloring and associated exemplar 2 × 2 image panels as in Fig. 5b. **f** UMAP with each point (a cell), colored by its cell line. Colored circles show the median coordinate of each cell line. **g** Hierarchical clustering of phenotype cluster based on occurrence across the 8 cell lines (top) and of cell line based on their phenotype cluster composition (bottom). **h** Clustering of individual CNN features into modules based on pairwise correlation between features using hierarchical clustering, as in (**d**).

As LIVECell images are single timepoint snapshots, not videos, we computed only the subset of the full SAM phenome comprising SAM-S shape (kernel ECC) and SAM-A appearance features (SA-features). SPOT found 709 informative SA-features from the test split dataset (415,518 cells) after feature preprocessing and identified 6 phenotype clusters with three dominant cell appearances: clusters 1,2,4 (neuronal like); cluster 3 (round and nuclear-like); cluster 6 (flat with large lamellipodia) and cluster 5 (image/segmentation artifact) (Fig. 6a). Coloring individual cells in the SPOT UMAP (Fig. 6b), and hierarchical clustering of the cell lines using phenotype cluster composition (Fig. 6c) identified 3 main morphological groups: group 1 (small and round shaped [BV2 and SKBr3]); group 2 (cells with distinctive morphology such as elongated neuronal like protrusions [SH-SY5Y & A172]) and large flat lamellipodia [A172 & SKOV3]); and group 3 (cobblestone-like cell clusters [MCF7, BT-474 & Huh7]). This grouping is in complete agreement with the organization of the 8 cell lines in the PCA plot generated using specifically computed morphological features in Fig. 1 of the original LIVECell paper^86^. This highlights the universality of SA-features and the ability of SPOT feature preprocessing to automatically select the most informative subset of SA-features. SPOT identifies 13 SA modules (Fig. 6d, Supplementary Fig. 6b) with shape (0.95) and distribution (0.86) features being the dominant contributors of phenotypic variation.

As an exemplar of AI deep learning (DL) learnt features, we trained an autoencoder-based convolutional neural network (CNN)^87,88^, which, together with its numerous variants^46–48,89^, represents the most popular method of deriving data-learnt features (“Methods”). Unlike the rationally engineered SAM phenome, autoencoder-based neural networks, comprise an encoder network which generates a fixed user-specified number of features given an input image, and a decoder networks to reconstruct the input image based on the encoder-generated features. We trained a 709 feature CNN autoencoder (matching the number of SAM-SA features) using individual image crops of the 1,011,452 cells in the train-val split dataset (Supplementary Fig. 6c). The trained encoder was then applied to the same test-split dataset to generate the 709 CNN features and then analyzed by SPOT identical to using SAM-SA-features. SPOT finds five phenotype clusters, but these identified less diverse and unique cell morphologies than SA-features: cluster 5 (round morphologies); cluster 4 (artifacts), clusters 1,2,3 (all others) (Fig. 6e). Nevertheless, CNN-SPOT’s UMAP does distinguish individual cell lines (Fig. 6f, Supplementary Fig. 6g, h). This suggests that CNN features have likely learnt primarily to distinguish cells by texture and not the finer differences in cell morphology. Consequently, CNN-SPOT’s spatial organization of cell lines in UMAP does not completely agree with the PCA in the LIVECell paper^86^ (Fig. 6f). Critically, hierarchical clustering based on CNN-features finds reduced similarity between the 8 cell lines, grouping only Huh7 and SK-OV-3 (Fig. 6g), and not reflecting what we observe in the images (Supplementary Fig. 6a). Applying SPOT module analysis, CNN-features have high information redundancy. The CNN-SPOT modules lack strong intra-module feature correlation (Fig. 6h) and many modules have similar representative images (Supplementary Fig. 6d). Critically, unlike the rationally-engineered SAM phenome, we cannot ascribe meaning to CNN features (hence they are generically named as Feature 1, 2, 3…etc.), preventing evaluation of the contribution of shape, appearance and motion or further interpretation of individual CNN-SPOT modules. Direct comparison of CNN-SPOT and SAM-SA-SPOT on the same dataset illustrates that CNN-SPOT is both less informative and less discriminative than SAM-SA-SPOT in identifying subtle morphological similarities between cell lines, notably missing both the neuronal and lamellipodia morphology clusters (Fig. 6a, e).

Finally, we also tested the informativeness of SAM-SA vs CNN features in training a machine learning classifier to predict cell line. For each, we trained a 2-layer multi-layer perceptron (MLP) on the train-val split and evaluated the MLP performance on the test split, as measured by accuracy and F1 scores (“Methods”). Both scores range from 0 (no classification) to 1 (perfect classification). SAM-SA phenome achieved an accuracy = 0.835, F1 = 0.842, significantly outperforming both the CNN-features without any feature normalization (accuracy = 0.658, F1 = 0.686) used in SPOT analysis, or after feature normalization (accuracy = 0.656, F1 = 0.688) (Supplementary Data 3). SAM-SA phenome also outperformed a state-of-the-art self-supervised learning architecture, DINOv3^90^ (best accuracy = 0.762, F1 = 0.773), and an equivariant feature learning architecture, O2-VAE^48^ (accuracy = 0.543, F1 = 0.584) with matching number of features (Supplementary Data 3). The confusion matrix, tabulating the fraction of cells in each cell line (columns) that was correctly predicted (rows), shows that while our SA phenome is near-perfect in uniquely identifying cell lines, CNN (Supplementary Fig. 6f), DINOv3 (Supplementary Fig. 6i, j) and O2-VAE (Supplementary Fig. 6k, l) features all confuse a significant fraction of BT-474 as MCF7, and SKOV3 as Huh7. O2-VAE also significantly confuses A172 and BT-474 cells (Supplementary Fig. 6l). Based on our SPOT analysis, the classification results reflect the inability of the CNN model to learn and generate features capturing finer morphological and textural details.

### SAM-phenome outperforms CNN-LTSM-learnt features on a HeLa cell mitosis video dataset

**Fig. 7:**
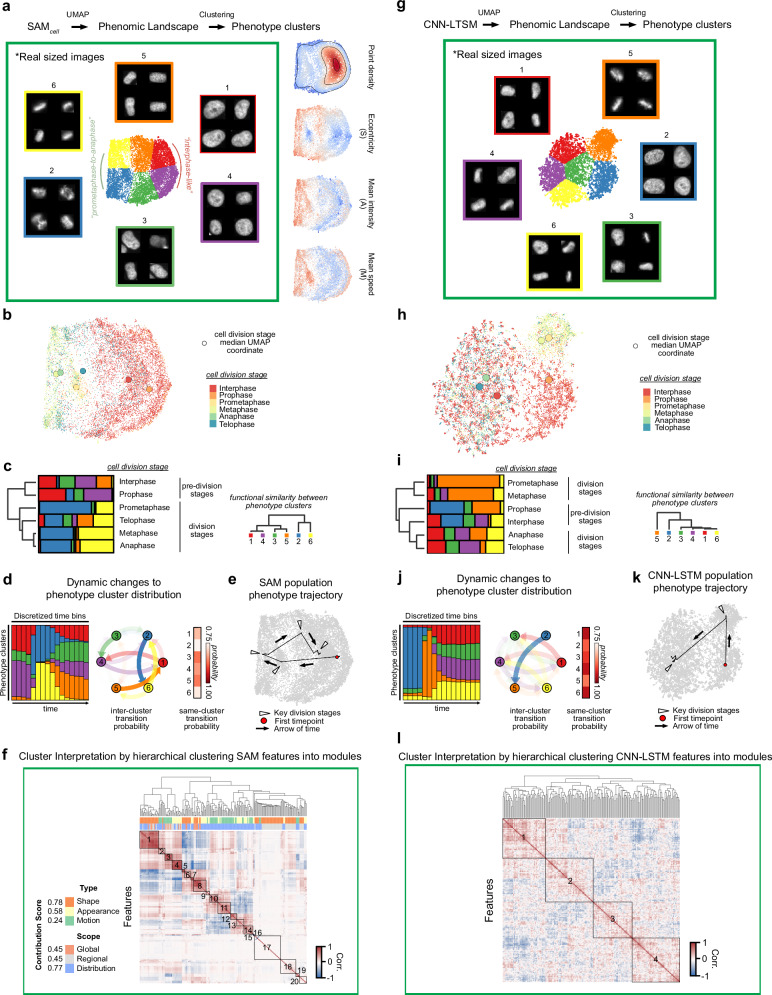
SPOT characterizes nuclei appearance during cell division. **a** SAM phenomic landscape constructed as in Fig. 5b. **b** SAM phenomic landscape with each point (a cell), colored by its cell line. Colored circles show the median coordinate of each cell line. **c** Hierarchical clustering using average linkage of the 6 stages of cell division based on their SAM-SPOT phenotype cluster composition (left) and of phenotype cluster based on their occurrence across the 6 phases of cell division (right). **d** Stacked barplot showing the relative frequency of each SAM-SPOT phenotype cluster over discrete time bins (left). Graph showing the next timepoint relative transition probability to a different phenotype cluster (center) and a heatmap showing the next timepoint probability of retaining the same phenotype cluster (right). Arrows are colored by the source cluster. The more transparent the arrow, the smaller the probability of the transition. **e** All cell population-level SAM phenotype trajectory summarizing the temporal evolution and phenotypic diversity across all cells and all videos. Starting timepoint is colored red. Black arrows show the directionality of time. **f** Contribution score of shape, appearance and motion, and global, regional and distributional features in explaining the dataset variance defined as the absolute value of the first principal component. **g** Phenomic landscape constructed by applying UMAP to the CNN-LSTM features. Cluster coloring and associated exemplar 2 × 2 image panels as in Fig. 5b. **h** Phenomic landscape with each point (a cell), colored by cell division stage. Colored circles show the median coordinate of each stage*.* **i** Hierarchical clustering of cell line using average linkage based on their CNN-LSTM-SPOT phenotype cluster composition (left), and of phenotype cluster based on occurrence across the 8 cell lines (right). **j** Stacked barplot of CNN-LSTM-SPOT phenotype cluster composition over time (left), next timepoint inter-cluster (center) and same-cluster transitions (right) as in Fig. 7d. **k** All cell population-level CNN-LSTM-SPOT phenotype trajectory as in Fig. 7e with starting timepoint colored red and black arrows showing the directionality of time. **l** Clustering of individual CNN features into modules based on pairwise correlation between features using hierarchical clustering (left).

To evaluate the full SAM phenome with respect to AI neural network learnt features, we applied SPOT to analyze a well-curated public HeLa cell mitosis video dataset (Fig. 7)^91^. This dataset tracks 326 individual HeLa cells, recording each cell as individual video sequences of 40 timepoints (total 13,040 video timepoints), as its nuclei, visualized by a histone H2B reporter, goes through all 6 distinct stages^92^ of mitosis (Supplementary Fig. 7a). Biologically, mitosis is staged based on the physical state of chromosomes and spindles. Here, our aim was to establish whether our universal SAM phenome could detect each stage. Unlike LIVECell, this dataset does not provide ground-truth segmentations. Consequently, we first performed SPOT stage 2, to segment nuclei in every frame by intensity-based thresholding (“Methods”). To ensure one video sequence corresponds to only one tracked cell, we retained the more centrally located of the two daughter nuclei after cell division. SAM-SPOT identified 186 SAM-features after preprocessing, and found 6 phenotype clusters (Fig. 7a), which appear to capture the changing chromosome appearance during mitosis: clusters 1,4 (interphase-like); clusters 2,6 (prometaphase-to-anaphase); and clusters 3,5 (intermediate-looking). SAM features and the organization of mitosis stage in the UMAP support this pattern (Fig. 7a, right, Fig. 7b). Most cell instances were in interphase, characterized by low eccentricity (i.e., circular nucleus) and low mean intensity, and were mapped to the right-side of the UMAP. In metaphase, chromosomes separate to polar ends, leading to elongated shape (high eccentricity), bright (high mean intensity) and large movements (high mean speed), mapping to the left-side of the UMAP. Notably, the median UMAP coordinate of each stage arranges clockwise in a cycle (Fig. 7b) whose separation distance predicts that the greatest SAM characteristic changes occur within the prophase-to-prometaphase and telophase-to-interphase transitions, consistent with observing larger changes in nuclear appearance in video sequences. As validation, hierarchical clustering of mitosis stage based on phenotype cluster composition identified a pre-cell division-related (interphase and prophase) and division-related (prometaphase, telophase, metaphase and anaphase) grouping (Fig. 7c). Moreover, clustering of phenotype clusters by mitosis stage composition generated three pairings of phenotype clusters that functionally partitions the 2D-UMAP into 3 vertical regions left-to-right, forming a gradient prophase-to-anaphase to interphase-like (Fig. 7b). The SAM-SPOT stacked plot clearly highlights the birth, expansion and disappearance of division-associated clusters 2,6 over time (Fig. 7d, left). The next timepoint inter-cluster transitions infer that these two clusters originate from clusters 1,3 and transition to clusters 3,5 (Fig. 7d, right). For additional validation, we expect clusters with less defined fate to transition to many clusters, and clusters with predetermined fate to transition to fewer clusters. Accordingly, we observe a lower Shannon entropy of cluster transition for prometaphase-to-anaphase clusters 2, 6 than interphase-like clusters 1, 4 (Supplementary Fig. 7c). The SAM population phenotype trajectory reveals the entire cycle of mitosis on the 2D UMAP (Fig. 7e), validating the implied clockwise transition from right-to-left side of the UMAP back to the right-side of Fig. 7b, and identifies 4 prominent turning points corresponding to key appearance changes at prometaphase; metaphase/anaphase; telophase/interphase and interphase/prophase (Fig. 7e). SAM module analysis computes the expected decreasing contribution of shape (0.78), appearance (0.58) and motion (0.24) features to phenotypic variation. While distribution features remain most informative, we found that the contribution scores of global/regional features were highest in this dataset out of all datasets studied. This is due to the evident appearance change from round to elongated nuclei (Supplementary Fig. 7a). SAM-SPOT generated 20 modules with strong intra-feature correlation (Fig. 7f). Importantly, representative images and features demonstrate that SAM-SPOT modules unbiasedly capture all prototypical nuclei shape-appearances in mitosis (Supplementary Fig. 7e).

As an exemplar of AI neural network learnt features, we trained an autoencoder-based CNN-LSTM^93–96^ (Convolutional Neural Network-Long Short Term Memory) network to generate features for an input video frame. This network modifies the fixed imaging autoencoder CNN network used for LIVECell dataset for videos, by incorporating an additional bidirectional LSTM layer^96,97^, enabling the CNN-LSTM encoder to generate 256 CNN-LSTM features with a temporal context of 5 frames (“Methods”, Supplementary Fig. 7b). The number of features was chosen to match the 186 SAM-SPOT features. As for LIVECell analysis, to ensure like-for-like comparison with SAM-SPOT, the CNN-LSTM was trained using, and applied to, all 326 nuclei video sequences (“Methods”). CNN-LSTM-SPOT found 6 phenotype clusters. However, compared to SAM-SPOT, individual exemplar images within a cluster showed inconsistent shape-appearance (Fig. 7g). The phenomic landscape indicates that this is due to CNN-LSTM features not capturing the morphological difference of the nucleus being circular in interphase and elongated during prometaphase/metaphase (Fig. 7h, Supplementary Fig. 7i, j). Unsurprisingly, clustering of mitosis stage by phenotype cluster composition failed to separate pre-division and division stages (Fig. 7i). The stacked plot does detect clear emergence of elongated nuclei as the cell divides (cluster 5, Fig. 7j, left) and the next timepoint inter-cluster transition analysis shows cluster 5 arises from cluster 2 and transits to clusters 4, 6 post-division (Fig. 7j, right). However, the CNN-LSTM population phenotype trajectory has only two turning points, verifying that CNN-LSTM features are insufficiently informative of mitosis stage, recognizing only the coarse morphology changes between round and elongated nuclei (Fig. 7k). SPOT module analysis also indicates that this is the primary drawback of CNN-LSTM-features, which only generated 4 SAM modules (Fig. 7l, Supplementary Fig. 7f), and captured only a subset of the phenotypic diversity of SAM-SPOT (Fig. 7f, Supplementary Fig. 7e).

Finally, we also assessed the possibility of training a classifier using SAM-phenome and CNN-LSTM features to classify mitosis stage (“Methods”). As predicted by SPOT analysis, SAM-phenome (accuracy = 0.595, F1 = 0.714) significantly outperformed CNN-LSTM-features without (accuracy = 0.492, F1 = 0.611) and with additional feature normalization (accuracy = 0.475, F1 = 0.576) (Supplementary Data 4). SAM-phenome also outperformed LSTM extensions of DINOv3^90^ (best configuration, accuracy=0.466, F1 = 0.646), and O2-VAE^48^ (best configuration, accuracy = 0.475, F1 = 0.612) features (“Methods”). Confusion matrix analysis shows that while our SAM-phenome clearly identifies each mitosis stage, CNN-LSTM (Supplementary Fig. 7g, h), DINOv3-LSTM (Supplementary Fig. 7k) and O2-VAE-LSTM (Supplementary Fig. 7l, m) features all significantly confused stages, notably unable to distinguish metaphase from prometaphase and anaphase. All these demonstrate that despite explicit incorporation of longer-term temporal dependencies with LSTM, SAM-phenome still unexpectedly outperformed deep learning features by providing both a more comprehensive and accurate characterization of nuclear morphodynamics in terms of shape, appearance and motion, and in training a superior mitosis stage classifier.

## Discussion

A major technological barrier preventing the effective application of label-free timelapse imaging to biological discovery is a lack of standardization with regard to the measurement and temporal analysis of shape, appearance, and motion (SAM) characteristics of imaged objects. To overcome this barrier, we took inspiration from how scRNA-seq analysis workflows^6^ have inspired similarly streamlined analysis of spatial transcriptomics^98^, and developed a 2185-feature SAM phenome and companion standardized workflow, SPOT, for “temporal image sequencing analysis”. Importantly, we demonstrated its universality and effectiveness by analyzing diverse public computer vision and biological datasets, including both small and large-scale datasets, notably A2D^80^, a challenging dataset of >350 YouTube videos; the LIVECell^86^ dataset, with >1.5 million label-free image cells; single-cell tracking datasets^99^; and 326 video sequences (> 5000 frames) of the nucleus during cell division^91^.

Rational construction of the SAM phenome was critical to this success (Fig. 1, “Methods”). A sufficient number of image features is necessary to be (1) comprehensive; characterizing the properties of shape, appearance, and motion at multiple spatial scales (global, regional and pixelwise (i.e., distribution)); and (2) robust to artifacts from image acquisition, segmentation and tracking; achieved by computing redundant features with complementary information e.g., computing both area and convex area. However, excessive features make each SAM phenome too ‘unique’, and hamper pattern finding. As such, our final full 2185-feature phenome represents a good balance. This principle is best seen in our benchmarking of different subgroupings of SAM-S features on MPEG-7 shapes (Supplementary Data 2). The full SAM-S (kernel ECC) phenome (ranked 1st, Supplementary Data 2) outperformed all subgroupings, but only due to preprocessing of ECC features (*n* = 1152, 11th) to reduce its dimensionality to 100 features before concatenation. The raw SAM-S phenome (*n* = 1614) significantly underperforms, ranking 6th, thus lower than its constituent subgroupings: Shape context (3rd, *n* = 300), Zernike moment (4th, *n* = 25) and geometrical (5th, *n* = 17). This principle is further evidenced by our supervised learning experiments on Brodatz-textured MPEG-7 shapes where our SA phenome consistently outperforms CellProfiler and TISMorph features except in the low training data regime where the best feature set was that with the least number of features. It might also be supposed that an imbalance in the number of shape, appearance and motion-associated features in our SAM phenome may bias analysis. However, we do not find evidence of this empirically. The contribution scores computed by SPOT in all studied datasets reflect the expected relative importance of shape, appearance and motion. Notably, SAM-SPOT analysis identified specific shape, appearance and motion-associated modules in a simulated organoid dataset relating to the predefined shape-, appearance- and motion-specific phenotypes^61^.

The SAM phenome is constructed using conceptually rotationally-invariant operations. However, empirically the numerical invariance is inexact. Consequently, in simulation experiments with handwriting digits (Supplementary Fig. 1c–h) and analysis of mitosis (Fig. 7), where there is greater pairwise similarity between 180 ± 90° rotated or mirrored version of an object and a second unrotated 0 ± 90° object, we observed splitting and mirroring of the same object type in the phenomic landscapes. This was not a problem for SPOT analysis, which nevertheless generated well-defined consensus phenotype trajectories (Fig. 7e), but may make biological interpretations more difficult. To increase invariance, we recommend users to compute the mean SAM phenome over desired data augmentations of each input (Supplementary Fig. 1g, h). Future work will also seek to improve the independence between shape, appearance and motion features for improved interpretability and accountability. Currently, appearance features also strongly capture shape due to the masking (Supplementary Data 2). We aim to develop more shape-invariant appearance features by texture tiling or differential geometric mapping approaches^89^. We also only capture motion by centroid displacement and optical flow. While this description is comprehensive, the motion features are co-dependent on the object type (Supplementary Fig. 1i–o). To increase the independence and thereby the complementarity of motion features, future work will model object motion as the sum of affine and non-rigid transformation, first performing affine image registration then applying optical flow. Also, our motion features exhibit reduced ability to discriminate motion directionality (Supplementary Fig. 1i–o). We aim to improve this in future work by adapting SIFT to directly encode orientation fields.

In contrast to the spirit of the SAM phenome, SPOT aims to introduce a conceptually standardized workflow specifying a set of systematic modularized processing steps for temporal analysis, for which this paper provides an implementation for all steps that is broadly-applicable. However, as in scRNA-seq, the actual algorithmic implementation can be customized by users to optimally analyze a dataset-of-interest, when generality is not required. Then, algorithms for each SPOT step can be adjusted to best handle the nuances of the specific dataset, e.g., training custom segmentation and tracking models, training AI-based features instead of the SAM phenome, or alternative dimensionality reduction methods such as t-SNE^100^ or PaCMAP^101^.

Deep learning (DL) AI models which learn directly from large input datasets have revolutionized computer vision, delivering state-of-the-art performance in many applications^56,58,102–105^. As such, there is much interest in AI models for biological discovery^55^. Here, we benchmarked SPOT analysis with SAM phenome against AI-based features generated by autoencoder-based models trained by self-supervised learning. We cannot feasibly compare all literature-proposed architectures. Thus, we used a U-Net^106,107^ model, one each for fixed and live-imaging, that is representative of the essential architectural components of the most prevalent family of models used in biological studies: CNN-based autoencoders^46,48,89,108^. Moreover, we trained the models using a training objective of data reconstruction, which makes minimal assumptions of the dataset. While SAM phenomes delivered rich insight for both LIVECell and nuclei division datasets (Figs. 6 and 7), the neural network models learnt only superficial information and could not identify subtle phenotypes. The SAM phenome also trained superior machine learning classifiers of cell line and mitosis stage than the neural networks, despite for the latter, allowing integrating information from a time window of five frames, double the two frames used for computing our SAM phenome’s motion features. Our SAM phenome further trained superior classifiers than state-of-the-art DINOv3^90^ and O2-VAE^48^ architectures optimized for feature learning on both LIVECell and nuclei division datasets. These performance gaps were not simply due to a lack of training data. Indeed, the classification gap was widest in the >1.6 million cell LIVECell. Together, our benchmarking results challenge the assumption that self-supervised AI models automatically generate the ‘optimal’ features for an analysis. Al models are overparameterized and have complex, hierarchical architectures which can ‘shortcut’, and learn only superficial features without additional constraints^109^. As such, they are notoriously difficult to interpret leading to unpredictable results, particularly on new unseen datasets^110–112^. In short, AI models need to be carefully and explicitly designed with custom SAM-aware architecture to generate features comparable to our SAM phenome. Lastly, feature interpretation is critical for biological discovery. AI model-features are not suitable for direct interpretation^112^. Further analysis is often necessary such as correlating individual features to explicit SAM measurements like area^46,113^ and causal tracing^114^. Most detrimentally, being data-driven, AI models trained separately on different unique datasets necessarily yield different, unique feature sets, even if we fix the number of features, i.e., feature 1 in dataset 1 is not the same feature 1 in dataset 2. Also, despite being a foundational model and trained on a large and diverse corpus of computer vision datasets, pretrained DINOv3 features were significantly worse than either fine-tuned or retrained DINOv3 features (Supplementary Data 3). In contrast, features in our SAM phenome are dataset-agnostic, avoiding any need to retain large raw libraries for retraining. Feature 1 will always be ‘maximum curvature’. Importantly, by design, each feature can be associated with a distinct concept (shape, appearance or motion) and scope (global, regional, pixel-based).

In summary, we have shown that a single set of rationally-designed imaging features can achieve remarkable off-the-shelf performance universally across diverse datasets, while maintaining interpretability not yet easily achievable without significant investment in designing and training custom deep learning models. Together with SPOT, our complete workflow effectively democratizes the complexities of video analysis to the wider scientific and technology community, enabling the detection and characterization of dynamic imaging phenotypes as readily as genotypes in scRNA-seq.

SPOT is currently implemented for 2D videos but this is not a critical limitation. In a companion paper^61^, we demonstrate that SPOT can accurately and comprehensively characterize 3D organoids from 2D projections. SPOT can be universally applied to analyze both fluorescently-labeled and label-free live-cell imaging. Future work will develop a native 3D SAM phenome and investigate how best to extend appearance motion features to multiplex live-cell imaging. We provide SPOT as an installable Python package, which includes computation of the SAM phenome with only standard library dependencies to facilitate customization and extension. For example, users may further augment the SAM phenome to include additional features that are SAM-based or from different modalities e.g., genetics-based, computed by other external software.

## Methods

### Construction of 2185 SAM phenome

For Shape: we measured 4 area and perimeter statistics to capture size (area, convex area, perimeter and the equivalent circle diameter), 7 shape factor based statistics to capture shape (area-to-perimeter aspect ratio, major axis length, minor axis length, major-to-minor axis length ratio, moment eccentricity, solidity, extent), 7 curvature based statistics to capture the contour complexity after multiplying by the object’s equivalent diameter to make it scale-invariant (maximum, minimum, mean, standard deviation, skew and kurtosis of curvature, and mean absolute curvature), and 5 centroid distance statistics to capture variation of boundary points with respect to cell centroid, as a point of reference (maximum, mean, coefficient of variation, the ratio of maximum-to-minimum distance of a boundary point to the object centroid, and maximum distance between two boundary points) as global features; Hu moments^66^—a translation, scale and rotation-invariant shape descriptor (total 7 features) based on lower order image moments, Zernike moments^64,67^—rotation-invariant shape descriptor based on decomposing the binary shape into its lower-order radial spatial frequencies (total 25 features), and Fourier features^64,65^—a rotation-invariant shape descriptor based on applying Fourier analysis to decompose the contour into low and high fluctuations, as local-regional features (total 99 features); a histogram of chordal distances (total 8 features with each histogram being a feature), Euler characteristic curve^68^ (ECC)—a topological descriptor based on analyzing the topology of an object at thresholds across a number of directional axes (total 1152 features, considering 36 directions, 32 thresholds), and shape context^70^—a translation, scale and rotation-invariant descriptor based on capturing the pattern of the contour locally around select ‘keypoint’ boundary points (total 300 features, considering 12 angle bins, 5 distance bins, for 5 equidistantly selected boundary points) as local-distribution features.

For Appearance: we measure the mean and standard deviation of intensity values (total 2 features) within the object as global features; the mean and standard deviation of intensity values in each of the three concentric regions, representing an object’s border, middle and inner core, and generated by partitioning the object’s area into 3 bands of equal distance from its boundary (total 6 features) as local-regional features; Haralick features^71^—a descriptor of local image texture based on measuring statistics of the co-occurrence of a pixel’s intensity value to that of all neighboring pixels within a distance cut-off (total 13 features), and Scale-invariant Feature Transform (SIFT)^73,115,116^—a translation, scale, and rotation-invariant descriptor of local texture around a keypoint based on equipartitioning the image into 4 × 4 image patches, and constructing a histogram of the direction of image gradients using 8 angle bins, and concatenating the histograms from all image patches (total 128 features), as local-distribution features.

For Motion, we compute first the dense optical flow^62^ for each video using successive frames, giving a velocity vector at each image pixel at a time t. We additionally compute the curl and divergence of the optical flow, illustrated in Fig. 1. Curl quantifies the extent of rotational motion around a pixel (positive and negative value for anti-clockwise and clockwise rotation, respectively). Divergence quantifies the extent a pixel is a local motion source (positive divergence, local velocity vectors radiate circularly outwards from this pixel) or a motion sink (negative divergence, local velocity vectors radiate circularly inwards into this pixel). We then measure as motion features the mean and standard deviation of the displacement in the object contour between time t and t+1 as mean boundary speed statistics (total 2 features), and the mean and standard deviation of the magnitude, curl value, and divergence value of the optical flow within the object (total 6 features) as global features; the mean, standard deviation, and a 24-bin histogram of the optical flow magnitude in each of the same three concentric bands used for appearance, as local-regional features; SIFT applied to the optical flow magnitude (Speed SIFT, 128 features), curl value (Curl SIFT), and divergence value (Divergence SIFT) images as local-distribution features.

### Validation datasets

#### Perturbed handwriting dataset for testing SAM phenome invariance properties

One exemplar image was selected for each of the ten digits 0–9 from the test split of the standard handwriting dataset, MNIST^74^. Each digit was rotated 12 times to span 0–360° in increments of 30°. To generate flipped versions, rotated versions of each digit were left-right image flipped (Python numpy function *numpy.fliplr*). To generate polar orientation displaced versions, the numpy roll function was used to shift each rotated digit image in the 8 principal directions (up, down, left, right, and four diagonals) by 2 pixels. To simulate rotational motion in the next frame, all rotated digit images were further rotated by the specified angle in the anti-clockwise direction (positive sign) and the same angle in the clockwise direction (negative sign). To compute SAM phenomes, the images were binarized by thresholding and infilled to extract the largest external contour. For motion features, the optical flow was computed between the current “non-additionally-rotated” and next “additionally-rotated” image.

#### Computer vision datasets for testing SAM phenome

Computer vision datasets with manually curated reference annotations were used to validate that our proposed SAM phenome was able to capture and discriminate heterogeneity and be universally applicable as-is without having to refine or define new features. Performance was assessed quantitatively by comparing the result of k-means clustering on the computed respective SAM feature set, where the number of clusters, k was set to the known number of classes in the given dataset. Two standard quality of clustering metrics were used. Adjusted mutual information (AMI, 0–1, *scikit-learn metrics.adjusted_mutual_info_score*) is an adjustment of the Mutual Information (MI) metric which measures the similarity between two clusterings to account for chance, a value of 1 represents perfectly concordant clustering. Adjusted rand index (ARI, −0.5–1, *scikit-learn metrics.adjusted_rand_score*) is an adjustment of the Rand Index metric, which computes a similarity measure between two clusterings by considering all pairs of samples and counting pairs that are assigned in the same or different clusters in the predicted and true clusterings, to account for chance. It is 1 for perfect concordant clustering and lower bounded by −0.5 for particularly discordant clusterings. For the A2D dataset, we also assessed the performance of training a supervised classifier on our SAM features. Performance was reported using the balanced accuracy score (labeled only as ‘accuracy’ in figures), which is the average of the recall obtained on each class to account for imbalanced classes, and F-score (or F1-score), defined as 2*precision*recall/(precision + recall) for a single class. We report the mean across all classes weighted by the number of true instances in each class to account for class imbalance.

#### MPEG-7 shape dataset to test shape

The MPEG-7 Core Experiment CE-Shape-1 (shortened to MPEG-7) database^76^ is a standard shape dataset comprising a total of 1400 shapes as binary images; 20 unique image examples each of 70 different shape classes representing different unique everyday objects such as bone, bat, heart, horseshoe, octopus, and turtle, captured with different sizes, rotations, and poses (Fig. 3a). For each image, we binary dilated by 3 pixels, topologically simplified by binary-infilling holes and extracting the external boundary of the shape as a contour of (x,y coordinates) using marching squares (scikit-image *find_contours*). For shapes where the binary-infilling produces multiple disconnected masks (due to a fragmented contour), only the largest connected mask was analyzed. The number of contour coordinates varies across shapes. To ensure the same dimension of SAM phenome is computed, the boundary contour of each shape is resampled to 200 equidistantly spaced boundary points. All SAM-S features were computed and were used to conduct the analysis in Fig. 3 with no other filtering of features except for kernel map dimensionality reduction of ECC features.

#### Brodatz texture database to test appearance

The Brodatz texture database^79^ is a standard dataset for testing appearance descriptors in computer vision. It comprises 112 unique images representing different grayscale patterns. Here, we used the normalized Brodatz texture variant^78^, an improved and more challenging dataset that uses an intensity normalization process to eliminate the original’s grayscale background effect. To use the Brodatz textures for testing, we derived a 22,400 image dataset by cropping each of the 640 × 640 texture images into 100 non-overlapping 64 × 64 patches and using both the patch and the patch rotated by 90°. SAM-A features were computed directly using the patches, treating the whole patch as an object with a square boundary contour and used to conduct the analysis in Fig. 3 with no other filtering of features. Features were, however, power normalized and z-scored as described for SPOT, Stage 4, step i below.

#### Brodatz textured MPEG-7 shapes to test shape and appearance

To test the combined shape and appearance features, a dataset of normalized Brodatz texture MPEG-7 shapes was created. Five shape classes from MPEG-7 were chosen by equally sampling the measured eccentricity, which was used as a proxy measure of shape complexity. Five normalized Brodatz texture images were chosen randomly. To texture the MPEG-7 shapes, their images were all standardized by rescaling to 128 × 128 pixels. Brodatz texture images were rescaled to 192 × 192 images. Then for each of the 20 unique images in each MPEG-7 class, we take each 192 × 192 Brodatz texture image, sample 20 random 128 × 128 cropped patches and multiply with the MPEG-7 image to create 20 different textured variants of the same basic texture and shape. This gives a total dataset of 10,000 image patches (5 shape classes × 20 images per shape class × 5 texture classes × 20 per texture class). SAM-SA features were obtained by concatenating computed SAM-S (SAM-S (kernel ECC)) and SAM-A features and used to conduct the analysis in Fig. 3 with no other filtering of features. Features were, however, power normalized and z-scored as described for SPOT, Stage 4, step i below.

#### A2D object-motion video dataset to test shape, appearance and motion features in real-life conditions

To simultaneously detect joint variations in shape, appearance and motion (SAM) in a setting reminiscent of real application to a heterogeneous dataset we used the A2D dataset^80^. This dataset comprises 3782 videos sourced from YouTube, depicting seven classes of moving objects (adult, baby, bird, cat, dog, ball and car) performing nine different movements (still, labeled for no movement), climbing, crawling, eating, flying, jumping, rolling, running, and walking (Fig. 4a). No object performs all actions. There are 43 unique object-movement pairs, the frequency of which are unequally distributed. A video may contain more than one instance of an object-movement pair or instances of different object-movement pairs^80^. For each video, 3–5 non-contiguous frames are annotated.

#### Generating consecutive frame object segmentations for A2D dataset to compute SAM phenomes

To compute the full SAM features for each annotated object instance, we first use the Segment Anything model^56^ and the bounding box of its annotated contour to re-segment the object. Then, we used optical flow^62^ to predict the object’s bounding box in the immediate next frame and use it as the prompt to segment its outline using the Segment Anything model. This ensures objects were segmented in the same way across frames.

#### Evaluating clustering and classification performance on A2D test dataset split

The A2D dataset has 43 unique object-movement pairs and is pre-split into 3036 training videos and 746 testing videos. For both clustering and classification analysis, we use object instances in the training videos to set the parameters of the k-means and classifiers, reporting performance only on objects in the testing videos. Full SAM features are defined by concatenating computed SAM-S (SAM-S (kernel ECC), *n* = 562), SAM-A (*n* = 149) and SAM-M (*n* = 422) features and was used to conduct the analysis in Fig. 4 with no other filtering of features. Features were, however, power normalized and z-scored as described for SPOT, Stage 4, step i below.

#### A2D object and movement type similarity graph analysis from UMAP median coordinate

Using the median 2D-UMAP coordinates of each object, movement or object-movement type, compute the pairwise affinity matrix between types defined as ${\rm{affinity}}={e}^{-\frac{D}{\mu \left(D\right)}}$ where D is the pairwise Eucliean distance matrix between coordinates and $\mu \left(D\right)$ is the mean value of the D matrix. The final similarity graph plots a connection between type i and type j if the affinity between types i and j is greater than the mean value of the affinity matrix (after excluding the main diagonal self-connections).

### Biological cell datasets for testing SPOT analysis

#### LIVECell dataset of diverse cell morphologies to test SPOT analysis

The LIVECell^86^ dataset consists of 5,239 manually annotated, expert-validated, Incucyte HD phase-contrast microscopy images with a total of 1,686,352 individual cells annotated from eight different cell lines: A172 (glioblastoma), BT-474 (ductal breast carcinoma), BV2 (microglial mouse cells), Huh7 (hepatocyte), MCF7 (human breast cancer), SH-SY5Y (neuroblastoma), SKBr3 (human breast cancer), and SK-OV-3 (ovarian cancer). The images and corresponding cell masks are split into one train-val folder of 3727 images and a test folder of 1512 images. We used only the test folder images for SPOT analysis. The provided cell masks were used as-is without checking for and removing potential partial cells on the image borders prior to SAM feature computation. As cells are fixed, only shape and appearance features were measured. The compiled SA-phenomes were filtered and processed as described below but without performing those preprocessing steps specific for videos where time information is available and SPOT analysis (Stage 4) was applied. For comparing the training of a classifier based on SPOT SAM or neural network features, we used the train-val folder images to train the classifier and the test folder images to evaluate performance.

#### Single-cell tracking datasets to test SPOT temporal analysis

We selected two datasets (U373 and HL60) from the 2D single cell tracking challenge^99^ with distinct and easily interpretable phenotypes (distinct cell morphologies as U373 cells migrate, and distinct appearance changes as HL60 cells divide) to test SPOT analysis (Stage 4). Each dataset was comprised of two unique videos, with each video’s outline annotation and temporal tracking information already provided for all timepoints, which we use to avoid introducing additional errors in segmentation or tracking in SPOT, Stage 3. Full SAM features were computed and compiled for all annotated cell instances that were fully present in the imaged field-of-view. Cells on the border which were partially in-view were removed prior to SAM feature computation and the corresponding single cell tracks truncated correspondingly into separate contiguous tracklets. The compiled SAM phenomes were filtered and processed as described below and SPOT analysis (Stage 4) was applied to both datasets in the same manner, using all the data instead of setting a percentile cutoff to exclude potential outliers when computing the representative phenotype cluster exemplar images.

#### HeLa cell histone H2B dataset to test SPOT temporal analysis

We use a published dataset of 7 timelapse microscopy movies of human tissue culture cells (HeLa ‘Kyoto’ cells) expressing a fluorescent chromatin marker (histone H2B–monomeric (m) Cherry)^91^. The movies comprise 326 video sequences, whereby each sequence tracks a unique single cell, showing only its nucleus and the change in nucleus morphology as the cell undergoes interphase and the five phases of mitosis (prophase, prometaphase, metaphase, anaphase and telophase). For each frame the mitosis stage is provided but not the nuclei segmentation. To compute SAM features, we custom segmented the nuclei frame-by-frame by binary intensity thresholding. Briefly, each image was contrast enhanced by clipping the intensity to the 2nd and 99th percentile; subject to adaptive histogram equalization (*skimage.exposure.equalize_adapthist*) with a clip limit of 0.02; and median filtered with a size of 3 pixels. The image was binary Otsu thresholded to generate the segmentation which was then postprocessed by morphological binary closing (disk kernel, radius = 3 pixels) and binary filling holes. Connected component analysis was then applied to retain the spatial component closest to the image centroid. To correct for, particularly in anaphase, two separated chromosomes being erroneously segmented as one, watershed segmentation was applied for separation, only the most centrally located chromosome was retained in the image for SAM feature computation. Full SAM features were computed for each segmentation. The compiled SAM phenomes were filtered and processed as described below and SPOT temporal analysis (Stage 4) was applied.

### CellProfiler features computation, processing and comparison with SPOT

#### Computation of CellProfiler shape-related metrics

All 1400 image files in the MPEG-7 dataset were uploaded to CellProfiler v4.2.6. The images were processed using CellProfiler’s ‘ConvertImageToObject’ and ‘MaskObject’ modules in succession. The ‘MeasureObjectSizeShape’ module was then used to generate shape-related metrics derived from object boundaries. These metrics were exported using the ‘ExportToSpreadsheet’ module, before being filtered to remove irrelevant columns (such as centroid measurements) and non-numerical columns prior to analysis.

#### Computation of CellProfiler appearance-related metrics

All 22,400 images in the data augmented Normalized Brodatz dataset were uploaded to CellProfiler v4.2.6 (112 Brodatz image classes x 200 patches per Brodatz class). Generation of appearance-related metrics was completed by grouping the images into 10 batches to prevent crashing. Within each batch, patches were grouped based on their image of origin. The ‘MeasureGranularity’, ‘MeasureTexture’, ‘MeasureImageIntensity’, ‘MeasureImageQuality’, and ‘MeasureImageSkeleton’ modules were run in succession, selecting for all metrics available to be computed within each module’s settings, wherever relevant. These metrics were exported using the ‘ExportToSpreadsheet’ module, and filtered to remove duplicated, irrelevant (such as execution time-related), and non-numerical columns prior to analysis.

#### Computation of CellProfiler shape and appearance-related metrics

The 10,000 images in the Brodatz textured MPEG-7 shapes dataset we created were cropped using the tightest bounding box around the shape, then uploaded to CellProfiler v2.4.6. The appearance-related metrics were computed at the whole-image level for each crop, a design choice made due to inadequate performance of the ‘ConvertImageToObject’ module on this dataset. The shape-related metrics are identical to those calculated for the entire MPEG-7 dataset and thus were not recomputed. All appearance-related metrics were computed by running the ‘MeasureGranularity’, ‘MeasureTexture’, ‘MeasureImageIntensity’, ‘MeasureImageQuality’, and ‘MeasureImageSkeleton’ modules in succession. These metrics were exported using the ‘ExportToSpreadsheet’ module, and filtered to remove duplicate, irrelevant, and non-numerical columns prior to analysis. The shape-related metrics and appearance-related metrics were concatenated to form the combined CellProfiler shape-appearance descriptor.

#### Analysis of CellProfiler computed features

To ensure like-for-like comparison, CellProfiler-computed features were handled and processed in the exact same manner as SPOT’s SAM features. This was the case for each relevant validation dataset.

### TISMorph features computation, processing and comparison with SPOT

#### Computation of TISMorph shape-related metrics

All 1400 image files in the MPEG-7 dataset were processed using the official Github code (https://github.com/Elaheh-Alizadeh/Quantifiction-of-shape-and-structure), treating each image as *Cell_Binary*. We modify the *CalculateParameters.m* and execute only the spreading and irregularity measures related-code to compute shape-related metrics. For the code to work, non-square images had to be made square by appropriate zero padding, binary infilling and retaining the largest spatial component.

#### Computation of TISMorph appearance-related metrics

All 22,400 images in the data augmented Normalized Brodatz dataset were processed using the official Github code (https://github.com/Elaheh-Alizadeh/Quantifiction-of-shape-and-structure), treating each image as *Cell_Gray*. We modify the *CalculateParameters.m* and execute only the texture measures related-code to compute appearance-related metrics. This also required a *Cell_Binary*. As the full Brodatz image is a texture, i.e., its binary is one everywhere, we artificially pad each Brodatz image with a one pixel wide zero border.

#### Computation of TISMorph shape and appearance-related metrics

The 10,000 images in the Brodatz textured MPEG-7 shapes dataset were processed using the official Github code (https://github.com/Elaheh-Alizadeh/Quantifiction-of-shape-and-structure) with modifications to extract shape-related and appearance-related metrics as detailed above, and concatenated.

#### Analysis of TISMorph computed features

To ensure like-for-like comparison, TISMorph-computed features were handled and processed in the exact same manner as SPOT’s SAM features. This was the case for each relevant validation dataset.

### CNN autoencoder features computation, processing and SPOT analysis

#### Training data

Individual cells of the LIVECell dataset were cropped using the bounding box of their segmentation mask, defined by upper left corner coordinates, $({x}_{1},{y}_{1})$ and lower right corner coordinates $({x}_{2},{y}_{2})$ where ${x}_{1},{x}_{2}$ is the minimum and maximum value of the x-coordinate, and ${y}_{1},{y}_{2}$ is the minimum and maximum value of the y-coordinate across all pixels comprising its segmentation. Cropped images were resized to a standard size of 64 × 64 pixels, the same size used by SPOT to compute SAM features. Image intensities were min-max normalized per image to be in the range 0–1. Cells from the train-val dataset were used for CNN autoencoder training, reserving the test dataset for SPOT analysis and comparison with SAM-SPOT.

#### Model training

A convolutional neural network (CNN) was trained using Keras with Tensorflow backend with the Adam optimizer (default parameters) for 100 epochs, a batch size of 128 images and mean absolute error loss. The encoder network uses 4 convolutional 2D layers with 3 × 3 kernel, downsampling by stride 2 between layers. The first layer uses 32 filters, with the filter number doubling for each subsequent layer. To compute the 709 CNN features, the encoder output is flattened, processed by a dense layer of 1024 units, then by a dense layer of 709 units. The decoder network mirrors the encoder in reverse, transforming the 709 CNN features back to an image output. ReLU activation was used for all layers throughout.

#### SPOT Analysis of CNN autoencoder features

The 709 CNN features per image were used as-is with no other preprocessing. The same SPOT analysis with identical parameters as for SAM-SPOT was performed using the CNN features.

### CNN-LSTM autoencoder features computation, processing and SPOT analysis

#### Training data

The HeLa cell histone H2B dataset^91^ provides already cropped and centered nuclei videos for analysis. We resize individual video frames to a standard size of 64 × 64 pixels, the same size used by SPOT to compute SAM features. Image intensities were min-max normalized per image to be in the range 0–1. The individual cell videos were processed to generate all possible continuous 5-frame image sequences, that is frames 1 to 5 is one sequence, 2 to 6 another, 3 to 7 another, etc. (Supplementary Fig. 7b).

#### Model training

The convolutional neural network (CNN) architecture used for the CNN autoencoder analysis of the LIVECell dataset was adapted to incorporate temporal video information into model training. We apply the same CNN encoder to each frame of the input 5-frame sequence, process the outputs using a bidirectional LSTM layer of 16 filters to incorporate time dependencies, and then apply a dense layer of 186 units followed by the same CNN decoder to each frame to transform outputs back to images. This CNN-LSTM model was trained using Keras with Tensorflow backend for 100 epochs with a batch size 128 sequences and mean absolute error loss. After training, the CNN encoder can be used to extract the 186 CNN-LSTM features for single timepoint images.

#### SPOT analysis of CNN-LSTM autoencoder features

The 186 CNN-LSTM features per image were used as-is with no other preprocessing. The same SPOT analysis with identical parameters as for SAM-SPOT was then performed using the CNN-LSTM features.

### Assessment of classification performance using SPOT SAM and CNN autoencoder features

#### LIVECell dataset^86^

Due to the large size of this dataset, we trained a two-layer multi-layer perceptron (MLP) classifier with categorical crossentropy loss to predict the cell line from input features in Keras with Tensorflow backend. We weight the loss inverse to the number of cells in each cell line to account for the discrepancy. Each layer used 100 units and tanh activation. The Adam optimizer (learning rate = $5\times {10}^{-4}$, ${\beta }_{1}=0.5$) was used to train the minimum validation loss model with early stopping (patience = 5, min_delta = 0.0005). We train the optimal classifer for the test folder for both SPOT SAM and CNN autoencoder features by using the test folder as validation set for early stopping. We use the preprocessed SPOT SAM features (709 features) for training the classifier. All dataset-dependent preprocessing steps such as zero-valued and high variance features, kernel transform dimensionality reduction of ECC shape features, standard scaling and power transform normalization was set based on the train-val folder images and then applied to the test folder images to ensure features were processed in the same manner. As the CNN autoencoder features (709 features) were learned from images directly, we used them as-is to train the classifier without any preprocessing.

#### HeLa cell histone H2B dataset^91^

The total number of nuclei is relatively small compared to LIVECell (12,714 = 326 video sequences x (40-1) frames), therefore we trained a support vector machine (SVM) classifier (using the scikit-learn library: RBF kernel, C = 0.1, random_state=0, class_weight=‘balanced’, decision_function_shape=‘ovr’) to predict interphase and the five mitosis stages (prophase, prometaphase, metaphase, anaphase and telophase) from input features. The video sequences were evenly split according to the 7 videos they originate from to construct training (videos ‘0037’, ‘0039’, ‘0042’, ’0046’) and testing (videos ‘0038’, ‘0040’, ‘0045’) video frames. Classifier performance was reported only using the testing video frames. We use the preprocessed SAM features (186 features) used in SPOT analysis to train the SVM classifier. We use the CNN-LSTM autoencoder features (186 features) learnt from video frames directly, as-is to train the SVM classifier without any preprocessing.

### Additional neural network feature learning

#### DINOv3^90^

DINOv3 models (ViT‑Base/16) were trained for each of the LIVEcell and HeLa cell histone H2B mitosis datasets. For each dataset, we extracted the features given by the CLS token from (i) the pretrained model with CLS token dimensionality closest to the number of features of the respective filtered SPOT-SAM phenomes, (ii) a fine-tuned model initialized with the weights of the pretrained model, and (iii) a retrained model starting from randomly initialized weights. Models were trained using the official GitHub code (https://github.com/facebookresearch/dinov3) with AdamW optimitizer using DINOv3’s composite self‑supervised objective, which integrates DINO, iBOT, KoLeo, and Gram losses. We use the same training data as for the CNN and CNN-LSTM but upscaled input image patches to 224 × 224 pixels to utilize the standard multi‑crop augmentation protocol used in DINOv3 training. Training was conducted on NVIDIA A100 GPUs (40 GB). For the video HeLa mitosis dataset, the DINOv3 features were used instead of raw image patches to train a multi-layer perceptron LSTM model to integrate temporal information across consecutive five frame sequences, with model parameters configured corresponding to the same layers used to train the CNN-LSTM. The MLP encoded DINOv3 features were then used as the individual frame image features in the same manner as done for the CNN-LSTM.

#### O2-VAE^48^

O2-VAE models were trained for each of the LIVEcell and HeLa cell histone H2B mitosis datasets. Each model was trained using the official GitHub code (https://github.com/jmhb0/o2vae) and modifying the *configs/config_mefs.py* file to set the number of features in the vae bottleneck layer to the same number as DINOv3 and filtered SAM-SPOT features. We use the same training data as for training the CNN and CNN-LSTM. For the video HeLa mitosis dataset, similar to DINOv3, the per-frame trained O2-VAE features were used instead of raw image patches to train a multi-layer perceptron LSTM model to integrate temporal information across consecutive five frame sequences, with model parameters configured corresponding to the same layers used to train the CNN-LSTM. The MLP encoded DINOv3 features were then used as the individual frame image features in the same manner as for the CNN-LSTM.

### Determining the optimal number of boundary points used to represent 2D shapes

We used the diverse shapes of the MPEG-7 dataset to determine the optimal number of boundary points. For each object, we use the contour extracted using marching cubes (scikit-image *find_contours*) as described above as its ground-truth reference boundary. Then, for each object, we resampled its reference boundary with n equidistantly spaced boundary points, increasingn from 10 to 1000 at increments of 50. The directed Hausdorff distance, H(A,B) measures the largest of all distances from a point in a point set, A to the closest point in another point set, B. In general, H(A,B)≠H(B,A). We therefore defined the shape reconstruction error of the n boundary point resampled contour as the maximum of its two directed Hausdorff distances ($\max (H(A,B),{H}(B,A))$)with the reference boundary. Our choice of n=200 equidistance boundary points balances this reconstruction error with using a minimal number of points to minimize hard disk storage (Supplementary Fig. 1a, b).

### Shape, appearance and motion Phenotype Observation Tool (SPOT)

SPOT sets out several stages for analysis as depicted in Fig. 2.

#### Stage 1: Preprocess video datasets

SPOT requires 2D videos without stage drift or other image translation artifacts. To ensure this, raw acquired microscopy videos are preprocessed. Preprocessing involves (i) if applicable, the projection of any 3D video frames where each frame is acquired as a 3D z-stack to single 2D images. As default, we use extended-focus projection for label-free microscopy imaging and maximum intensity projection for fluorescent imaging, to optimally retain object features and maximize the number of objects visualized in the 2D projection image. 2D videos are then (ii) frame-by-frame registered to remove any potential global translation motion. We do this by firstly detecting and removing any blurry frames, automatically detected as having a mean image intensity gradient magnitude one standard deviation less than the mean computed across all video frames. Fourier transform cross-correlation^117^ was then applied sequentially to consecutive frames to register the entire video. Registration is essential for extended long-time timelapse data acquired in multiple acquisitions which often shifts the imaging position, sometimes by a significant distance.

#### Stage 2: Detect video objects

Individual object instances must be segmented from video frames to compute Shape and Appearance features. To also compute all motion features, every object must be tracked to link an object instance with the matching instance in the next frame. Tracking is also essential to identify and remove any transient objects whose tracks only last a few frames. In this study, we used, unless otherwise described, the provided segmentations and object tracking of each public dataset, if available.

For general use, the SPOT code repository only provides a custom trained organoid bounding box detector and segmentation model, developed for our companion organoid study but no general segmentation models, as segmentation is often more optimal if fine-tuned to individual datasets and the specifics of video acquisition. We note general segmentation models for 2D images of both natural objects^56^ and cells^118^ exist, and readily obtained from their respective sources. SPOT does, however, provide a general tracker to track segmentations provided as bounding boxes based on spatial overlap across successive frames to generate temporally consistent tracklets. Notably, linking between consecutive frames is aided by using the computed dense optical flow to handle potential large shape, appearance and movement changes. Tracklets are built starting with each detected box in frame 0. The coordinates of each bounding box in the next frame, $(x\left(t+1\right),{y}(t+1))$, is predicted by fitting a rigid motion model (scale, translation, rotation), T to the local optical flow, $(\Delta x,\,\Delta y)$ within the box, i.e., $\left(x\left(t+1\right),{y}\left(t+1\right)\right)=\left(x\left(t\right)+\Delta x\left(t\right),{y}\left(t\right)+\Delta y\left(t\right)\right)=T\left(\left(x\left(t\right),{y}\left(t\right)\right)\right)$. Using the predicted coordinates after fitting $T\left(\left(x\left(t\right),{y}\left(t\right)\right)\right)$, the boxes are matched to actual boxes of detected objects in the next frame, frame 1, via linear assignment bipartite matching (c.f. Scipy *linear_sum_assignment*), using the pairwise cost matrix constructed from 1-intersection-over-union (IoU) between predicted and actual detected boxes in frame 1. A minimal IoU cut-off is used to determine a successful pairing (default IoU≥0.25, i.e., 1-IoU≤0.75). The tracklets of successfully matched boxes are extended. Tracklets with unsuccessfully matched boxes in frame 0 are also extended opportunistically using the motion model predicted coordinates. Boxes unsuccessfully matched in frame 1 start new tracklets, adding to the pool of running tracklets to extend in subsequent frames. The linking process is repeated frame-by-frame consecutively until the end of the video and all detected boxes are a member of a tracklet. Running tracklets that have not been successfully matched to a ‘real’ object box corresponding to a segmentation after a specified number of frames are ‘terminated’ and no longer considered. This tracking process is designed to ensure coverage of all segmentations throughout the length of the video. Tracklets can finally be filtered by setting threshold values for (1) minimal lifetime coverage (measured as a fraction of tracklet length) and (2) mean confidence detection score across all boxes composing the tracklet (e.g., confidence score predicted by object detector or segmentation probability map).

#### Stage 3: Computation of shape, appearance, and motion (SAM) features

SAM features were computed with custom code using, where possible, standard implementations available in established Python libraries such as scikit-image and OpenCV (see our GitHub code, https://github.com/fyz11/SPOT). Detailed descriptions and relevant references for each feature are provided in Supplementary Data 1. Motion features are only computable for an object instance at timepoint t if it has a corresponding instance in the next timepoint, t+1. Most features in our SAM phenome are dimensionless (Supplementary Data 1). Where a measurement is not dimensionless, all measurements were extracted in standard physical units of micrometer (μm) for length and hours (h) for time.

#### Stage 4: Temporal analysis of shape, appearance, and motion (SAM) features

This stage comprises two parts which can be performed independently and then mix-and-matched. Part A implements a streamlined workflow to monitor temporal changes in phenotypic heterogeneity. Two primary analyses are performed: (1) a SAM population phenotype trajectory providing a continuous readout of temporal evolution driven by majority phenotypic states and (2) automatically finding a finite number of discrete phenotype clusters to characterize the full phenotypic heterogeneity, and the temporal phenotype cluster composition and inter-cluster transitions within a condition provide further interpretation of its phenotype trajectory. Part B focuses on analysis interpretation by capturing the Pearson correlation between individual SAM features across all objects in the analyzed dataset to consolidate individual features into a smaller number of ‘SAM modules’, similar to molecular pathways. The SAM modules are used to retrieve exemplar object images from the dataset, and find the driving features for interpretation.

### Part A: Discovery and analysis of dynamic phenotypic heterogeneity

#### Step i: Generating filtered SAM phenomes for analysis

Raw computed SAM phenomes require filtering; to remove those features that are too small or zero-valued and those that exhibit too high a variation across the dataset to be analyzed. Specifically, we remove all features that are zero-valued across all object instances, and those with zero variance across all objects (from possibly different experiments) to be compared in the same analysis. High variance features (defined typically 2 ensemble standard deviations above the ensemble mean feature variance) are also removed, $\sigma \left({f}_{i}\right)\ge {\mu }_{f}\left(\sigma \left({f}_{i}\right)\right)+k\cdot {\sigma }_{f}\left(\sigma \left({f}_{i}\right)\right)$ where ${f}_{i}$ denotes feature i; σ the standard deviation across all object instances; and ${\mu }_{f},{\sigma }_{f}$ the ensemble mean and standard deviation across all feature variances. The remaining features are normalized so that the value of each feature is comparable across features (for example, to enable shape area to be considered with equal weighting to shape curvature, where the raw values differ greatly in magnitude). First, all curvature-associated features with units of length^−1^ are multiplied by the equivalent diameter to correct for the inherent bias of measuring decreased curvature for objects of the same shape but of different size. Second, a radial basis function (RBF) kernel is applied using the Nystroem approximation (c.f. scikit-learn *Nystroem*) to compress the raw, sparse and discrete Euler characteristic curve (ECC) features to 100 dimensions which we found yielded improved clustering performance and made the inclusion of ECC features more beneficial than its exclusion (see Supplementary Data 2, SAM-S (ECC) vs SAM-S (kernel ECC)). Third, for data acquired as separate batches, we correct feature values to remove the ‘batch’ variation using linear regression. We consider ‘batch’ as the primary experimental source of variation, for example, a ‘batch’ could be individual plates imaged on different days or different patients. Fourth, the (possibly batch corrected) feature values are normalized by applying standard scaling (scikit-learn *StandardScaler*) followed by a power transformation (scikit-learn *PowerTransformer*, method=‘yeo-johnson’). The normalized feature values or SAM scores are plotted as ‘Score’ in figures. For timelapse imaging, particularly for label-free imaging, ridge regression (scikit-learn *Ridge*, alpha=1) using the frame number as the dependent variable and the SAM score of each feature as independent variables is used to select only those features that exhibit significant temporal variation - those with an absolute regression coefficient value greater than the mean absolute regression coefficient value across all features.

#### Step ii: Low-dimensional embedding to construct the SAM phenomic landscape

The SPOT SAM phenomic landscape aims to capture and visualize all SAM-based phenotypic variations across every object, every timepoint and every video in a single 2D coordinate space. As such, though any dimensionality reduction can be applied (Supplementary Fig. 4b), for best results the dimensionality reduction method should ‘spread’ the SAM variation in 2D space instead of collapsing object instances into branches or singular points as in scRNAseq for lineage inference. Consequently, we chose to use Uniform Maniform Approximation and Projection (UMAP)^81^ developed based on mathematical theory of topological spaces via the Python *umap-learn* implementation to project preprocessed SAM features into 2 dimensions for plotting and analysis. The following default parameters are used: n_neighbors = 100, random_state = 0, spread = 0.5, min_dist = 0.5, metric = ‘euclidean’. For very large datasets (>1 million points), or if it is required to be able to add new object points after initial mapping, we suggest users instead use a dimensionality reduction method supporting batch-based evaluation such as parametric UMAP or deep neural autoencoders.

#### Step iii: Computation of the SPOT SAM population phenotype trajectory

To construct temporal SAM phenotype trajectories in the phenomic landscape, the total temporal duration of video acquisition was partitioned into equal time intervals. For each time interval, a heatmap density image (see below) was computed of only those individual object instances present during that time interval. The heatmap image was partitioned into high- and low-density regions using the mean density (± 2 or 3 standard deviations) as a cut-off. The mean UMAP coordinate of the high-density regions (possibly more than one) is computed as the phenomic landscape coordinate that best summarizes the distribution of majority phenotypes during this time interval. The full phenotype trajectory is formed by chronologically linking together the mean UMAP coordinates of high-density regions from all time intervals.

#### Step iv: Partitioning of the phenomic landscape into discrete phenotype clusters

Clustering is used to mutually exclusively partition the phenomic landscape into a discrete number of phenotype clusters such that the object instances within each phenotype cluster share similar SAM features. Here we use k-means clustering to generate approximately equal-sized phenotype clusters. This equates to using a noninformative prior of the occurrence of phenotypic states. If it is known or hypothesized or desired that phenotype clusters should have different abundances, for example, we expect rare phenotypes, then alternative clustering such as density-based clustering methods may be more suitable and can be used instead. Scikit-learn k-means clustering (random_state=0) was applied to the UMAP projected 2-dimensional coordinates to cluster individual object datapoints into a discrete number of ‘phenotype clusters’ representing a set of prototype SAM characteristics for the dataset. As such, these clusters do not necessarily represent only biological phenotypes but also capture salient imaging artifacts such as blur or out-of-focus objects. The number of clusters was determined from a minimum of two clusters to a maximum of 20 clusters guided by the minimum of the elbow method applied to the k-means inertia loss. Using these phenotype clusters, the phenotypic diversity of a collection of object instances can be described by a histogram recording the occurrence frequency of each prototype. This approach naturally reconciles multi-parametric phenotypic heterogeneity and is motivated by the success of bag of words/features approaches in computer vision^119^.

Auto-generating exemplar object instances for each phenotype cluster: the first principal component (PC) obtained by applying PCA to all object instances within a phenotype cluster provides a measure of the contribution of each object instance to the variance within the cluster. Object instances within a cluster are ranked in descending order of the first PC value and the top rankings are visualized as exemplars and are aligned with the first PC. As PCA is sensitive to outliers caused by errors in the initial object segmentation or tracking, ranking was performed only for instances for which the values of the first PC were less than a specified percentile (for A2D^80^, 90th percentile, for LIVECell^86^, 100th percentile and for the HeLa cell histone H2B dataset^91^, 75th percentile). For the single cell tracking datasets^99^, where both reference segmentations and tracking were provided, we use all object instances without exclusion (i.e., the 100th percentile as cut-off).

#### Step v: Monitoring phenotype cluster dynamics

##### Temporal phenotype cluster frequency stacked barplots

Each object instance is assigned the phenotype cluster into which its 2D-UMAP coordinates fall. As for phenotype trajectory computation, the total temporal duration of video acquisition was partitioned into equal time intervals. For each time interval, a normalized histogram of the number of object instances within the interval is constructed where (the frequency of phenotype cluster i) = (# object instances in time interval of cluster id i)/(total # object instances in that time interval). The normalized histograms for all time intervals are concatenated chronologically to form a stacked barplot of phenotype cluster frequency over time.

##### Next timepoint phenotype cluster transition probabilities

We compute the next timepoint phenotype cluster transition probability using the individual object tracking information. Each object instance is first assigned the phenotype cluster corresponding to its 2D UMAP coordinate. Then, using the object tracks from SPOT, Stage 2, we construct the phenotype cluster label timeseries for each tracked object (Fig. 5c). We consider the probability of phenotype cluster *i* at time *t* transitioning to phenotype cluster *j* at time *t* + 1, as a first-order Markov process, and estimate the transition probability from the observed frequency statistics across all object tracks, $P\left({C}_{t+1}=j\left|{C}_{t}=i\right|\right)=\frac{\#\left({C}_{t+1}=j\left|{C}_{t}=i\right|\right)}{{\sum }_{k=0}^{N}\#\left({C}_{t+1}=j\left|{C}_{t}=i\right|\right)\,}$ where #() denotes the total number of instances we observed cluster *j* succeeding cluster *I* and *N* the total number of phenotype clusters. Generally, transitions to another cluster are rare in a temporal sequence. Therefore, for analysis, we visualized the self-cluster transitions, i.e., from cluster *i* to cluster *i*, separately as a heatmap. We obtain the inter-cluster transition matrix by subtracted out the self-cluster transitions (diagonal entries of $P\left({C}_{t+1}=j\left|{C}_{t}=i\right|\right)$). The inter-cluster transition matrix was then renormalized so that each row summed to 1, $P\left({C}_{t+1}=j\left|{C}_{t}=i,{j}\ne i\right|\right)=\frac{\#\left({C}_{t+1}=j\left|{C}_{t}=i,j\ne i\right|\right)}{{\sum }_{k=0}^{N}\#\left({C}_{t+1}=j\left|{C}_{t}=i,j\ne i\right|\right)\,}$ and visualized as a graph. In the graph, arrows are drawn from *i* to *j*, and colored by cluster *i*. The opacity of the arrow representing a higher inter-cluster transition probability, transparent being 0 and solid between the upper probability cut-off visualized. To enhance the differences in the inter-cluster transition matrix, we raised the matrix to an exponent (γ=3 typically) and used a lower cutoff = 0.0, and an upper cutoff = 0.6, unless otherwise stated. The graph was generated using the Python hmmviz package. To compute the transition entropy for each phenotype cluster, the Shannon entropy was computed for each row of the inter-cluster transition matrix.

### Part B: Discovery and analysis of dynamic phenotypic heterogeneity

#### Step vi: Automatic grouping of filtered SAM phenome features into SAM modules

SAM modules group features that covary in the dataset, specifically features that exhibit greater Pearson correlation between those features in the same module than between those features in a different module. To identify SAM modules, we use all of the compiled SAM phenomes to be analyzed across all conditions. Following filtering and preprocessing above (SPOT Stage 4A, step i), the (# features) × (# features) pairwise Pearson correlation coefficient matrix between the retained and transformed SAM features is computed using all object instances as ‘samples’. To automatically group features into SAM modules and determine the number of SAM modules, we use hierarchical clustering with average linkage, together with clustering quality metrics. Hierarchical clustering generates a dendrogram whereby the root node groups all features as one, and progressive branch splitting corresponds to splitting of the larger parental grouping into unique smaller mutually exclusive groupings. At the bottommost leaves of the dendrogram, the individual SAM modules are the individual SAM features. To determine the optimal number of modules, we used as the clustering quality metric, the Davies-Bouldin (DB)^120^ score, defined as the average similarity measure of each cluster with its most similar cluster, where similarity is the ratio of within-cluster to between-cluster distances. The lower the DB score, the better the clustering, with the minimum DB score as 0. Computing the DB score as a function of the increasing number of modules corresponding to progressive dendrogram branch splits, we observe the DB score increases then decreases with typically, multiple peaks. We set the number of modules corresponding to the first peak in the DB score. This strikes a balance between having too few and too many modules to describe phenotypic heterogeneity.

#### Step vii: Finding exemplar object instances and evaluating SAM module expression in individual phenotype clusters

Principal component analysis (PCA) finds a linear transformation of the data such that the first coordinate or first principal component corresponds to the direction of greatest variance of the data. The expression of SAM module i for each object instance is defined as the first principal component after applying PCA to the preprocessed and normalized SAM features from SPOT Stage 4A, step i (i.e., the SAM scores of final features) that form SAM module i. As the normalized SAM features have zero mean and the same standard deviation, and as PCA without whitening preserves this property for SAM expression, we define a score of the extent an object instance expresses SAM module i as the expression of SAM module i minus the maximum expression for any other SAM module. For each SAM module, object instances are ranked in descending order of this score and the top instances are visualized as exemplars.

#### Scoring the contribution of feature type and spatial scope

Principal component analysis (PCA) finds a linear transformation of the given input data such that the first principal component corresponds to the direction of greatest variance of the data. SPOT uses this property to derive a score of the contribution of feature type; shape, appearance or motion and spatial scope; global, regional or distribution to the phenotypic variation in each dataset. Each feature in the SAM phenome is annotated with its feature type and spatial scope (see Supplementary Data 1). To compute the contribution of feature type, we evaluate first the ‘expression’ of shape, appearance and motion of each object instance, defined by the first principal component after applying PCA to the subset of preprocessed and normalized SAM features from SPOT Stage 4A, step i, categorized as shape, appearance and motion, respectively. PCA is applied again to all object instances’ shape, appearance and motion expression but treating the expression as ‘samples’ i.e., rows and object instances as ‘features’ i.e., columns. The absolute value of the coefficients of the first principal axis, associated with the first principal component, defines the contribution score of shape, appearance, and motion, respectively, to the data variance. The contribution of spatial scope is computed separately in the same manner.

#### Phenomic landscape density heatmaps

To produce flow cytometry-like density heatmaps of 2D UMAP projected points of object instances efficiently, we estimate the Gaussian kernel density of points using an image-based approximation. We rasterize the UMAP plot as an image of desired resolution (e.g., 2000 × 2000 pixels). Individual 2D UMAP coordinates $\left({uma}{p}_{x},{uma}{p}_{y}\right)$ are transformed to integer image coordinates using $\left({imag}{e}_{x},{imag}{e}_{y}\right)=\left(\left\lfloor {resolution}\times \frac{{uma}{p}_{x}-\min \left({uma}{p}_{x},{uma}{p}_{y}\right)}{\max \left({uma}{p}_{x},{uma}{p}_{y}\right)\,-\min \left({uma}{p}_{x},{uma}{p}_{y}\right)}\right\rfloor,\, \left\lfloor {resolution}\times \frac{{uma}{p}_{y}-\min \left({uma}{p}_{x},{uma}{p}_{y}\right)}{\max \left({uma}{p}_{x},{uma}{p}_{y}\right)\,-\min \left({uma}{p}_{x},{uma}{p}_{y}\right)}\right\rfloor \right)$, where ⌊⌋ is the floor operator, max is the maximum value of UMAP $\left(x,y\right)$ coordinates plus a padding value (we use 1) and min is the minimum of UMAP $\left(x,y\right)$ coordinates minus a padding value (we use 1). Then, each UMAP point for the condition under consideration contributes +1 to the image pixel value at the computed corresponding image coordinate, producing a count of the number of individual points that map to a particular image pixel. The resultant image is finally Gaussian smoothed with an automatically determined sigma, $\sigma=\frac{1}{4}\left({mean}\left({Euclidean\; pairwise\; distance\; between\; UMAP\; image\; coordinates}\right)\right)$. The full pairwise distance matrix between all points does not need to be used for computation. We found a random sample of 1000 points sufficient to obtain a smoothly varying density heatmap.

#### Clustering of SAM temporal trajectories

To cluster the temporal phenotype trajectories (computed as described above) of objects from different conditions, a pairwise distance matrix is constructed using multidimensional dynamical time warping (DTW) (through the *dtaidistance* Python package) to measure the distance between any two trajectories. Compared to pairwise Euclidean distances, the use of DTW accounts for variations in trajectory speed. The resulting distance matrix, D, is unbounded in magnitude; for clustering D is transformed into an affinity matrix, A with entries in the range from 0-1 using the formula, ${A}_{{ij}}=\exp \left(-\frac{{D}_{{ij}}}{{\rm{std}}\left(D\right)}\right)$ where std() is the standard deviation of distances in D. The rows of the affinity matrix are used as the features describing the trajectory of each condition. By default, the average linkage and the Euclidean metric were then used to hierarchically cluster different conditions by their features.

#### Statistics and reproducibility

All experiments were conducted using publicly available datasets. We used all available data without exclusion unless otherwise specified in the figure panel. No data was excluded. Statistical outliers were detected and filtered out during analysis as part of our SPOT workflow. We validated the replicability and general applicability of SPOT on publicly available datasets used for benchmarking. For computer vision: MPEG7 shape dataset, Brodatz textures, A2D dataset and MNIST dataset. For cell imaging: cell tracking challenge datasets, LIVECell and a Hela cell histone H2B dataset part of the CellCognition project. Blinding was not applicable in this study. For benchmarking we used the provided train/val/test splits when available. Randomization was used to create the train/test split for benchmarking mitosis stage classification in the Hela cell histone H2B dataset. Supplementary Table 1 documents all n numbers used in each figure panel.

### Reporting summary

Further information on research design is available in the Nature Portfolio Reporting Summary linked to this article.

## Supplementary information

Supplementary Information

Reporting Summary

Description of additional supplementary files

Supplementary Movie 1

Supplementary Data 1

Supplementary Data 2

Supplementary Data 3

Supplementary Data 4

Transparent Peer Review file

## Source data

Source Data

## Data Availability

All datasets used in this manuscript are publicly available and details are provided in the “Methods” and Supplementary Table 1. The filled, dilated, and textured MPEG-7 datasets we created are available on Zenodo (10.5281/zenodo.20648294)^121^. Source data are provided with this paper.

## Code availability

The developed general SPOT Python library (SAM-SPOT) used to compute and analyze SAM phenomes is available at GitHub, https://github.com/fyz11/SPOT and installable from the Python Package Index, PyPI for free academic use with example scripts and example data. An archived version with permanent DOI is available on Zenodo (https://zenodo.org/records/20629367)^122^. Further information and requests for resources should be directed to and will be fulfilled by the lead contact, Xin Lu (xin.lu@ludwig.ox.ac.uk).
