# Supplementary material for: Development of a universal imaging “phenome” using shape, appearance and motion (SAM) features and the SAM Phenotype Observation Tool (SPOT)
Source: Nat Commun. 2026 Aug 17;17:8409. doi: 10.1038/s41467-026-75505-8 (PMC13483437; doi:10.1038/s41467-026-75505-8)
Supplement: Supplementary file 1 [file 41467_2026_75505_MOESM1_ESM.pdf]

SUPPLEMENTARY INFORMATION

Development of a universal imaging "phenome" using Shape, Appearance and Motion (SAM) features and the SAM Observation Tool (SPOT)

Felix Y. Zhou[1,2,4,5,6^*], Adam Norton-Steele[1^], Lewis Marsh[1,3,7], Helen M. Byrne[1,3], Heather A. Harrington[3,8], and Xin Lu[1*]

[1]Ludwig Institute for Cancer Research, Nuffield Department of Medicine, University of Oxford, Oxford, OX3 7DQ, UK

[2]Lyda Hill Department of Bioinformatics, University of Texas Southwestern Medical Center, Dallas, TX, USA

[3]Mathematical Institute, University of Oxford, Woodstock Road, Oxford OX2 6GG, UK

[4]Present address: Department of Cell & Developmental Biology, Vanderbilt University, Nashville, TN, USA

[5]Present address: Center for Computational Systems Biology, Vanderbilt University, Nashville, TN, USA

[6]Present address: Vanderbilt Ingram Cancer Center, Vanderbilt University Medical Center, Nashville, TN, USA

[7]Present address: Novo Nordisk Research Centre Oxford (NNRCO), Innovation Building, Roosevelt Dr, Headington, Oxford, OX3 7FZ, UK

[8]Present address: Max Planck Institute of Molecular Cell Biology and Genetics, Dresden, Germany

^These authors contributed equally

*Corresponding authors

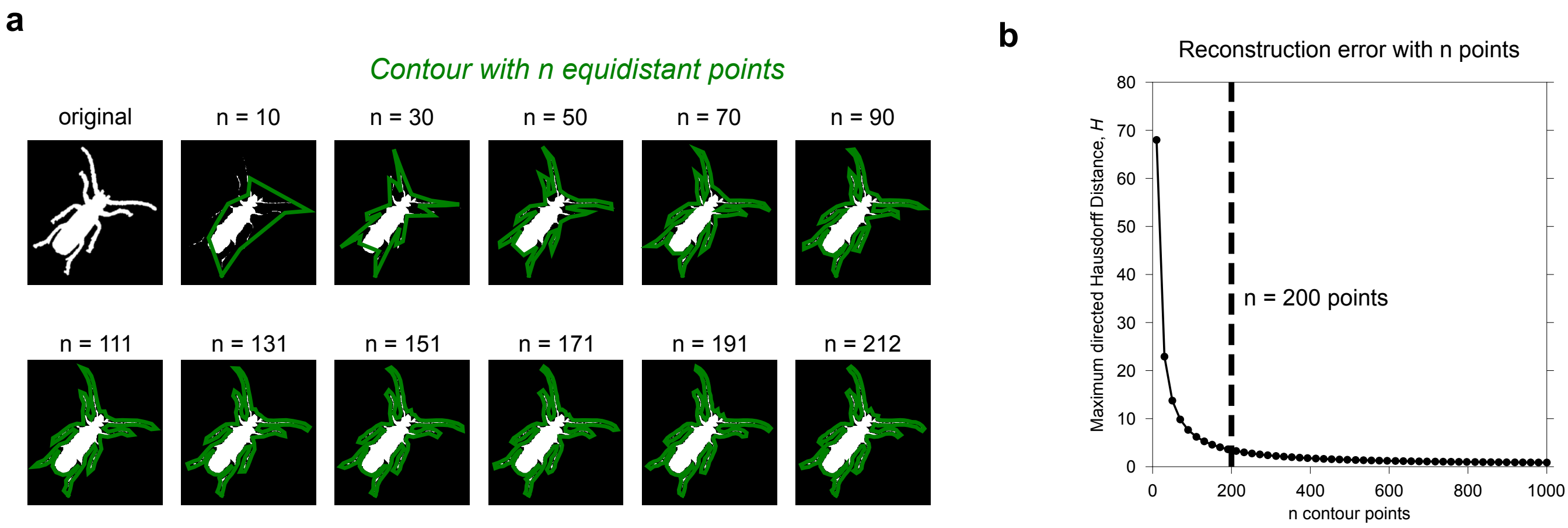

Supplementary Figure 1. Brightfield phase contrast imaging has limited depth resolution and extended focus projection of a z-stack to a 2D image. a) Individual z-slices of the acquired 8-slice z-stack at day 0 (left), its 2D maximum intensity projection (middle), and 2D extended focus projection used in this paper (right). Each z-slice is separated by a depth of 200 µm. b) Same as a) of the same organoids acquired at day 6. c) Reslice of the z-stack image along the indicated color line indicated in the corresponding z=8 xy-slice on the left. Black triangle mark the edge of the organoid. All scale bars: 250 µm.

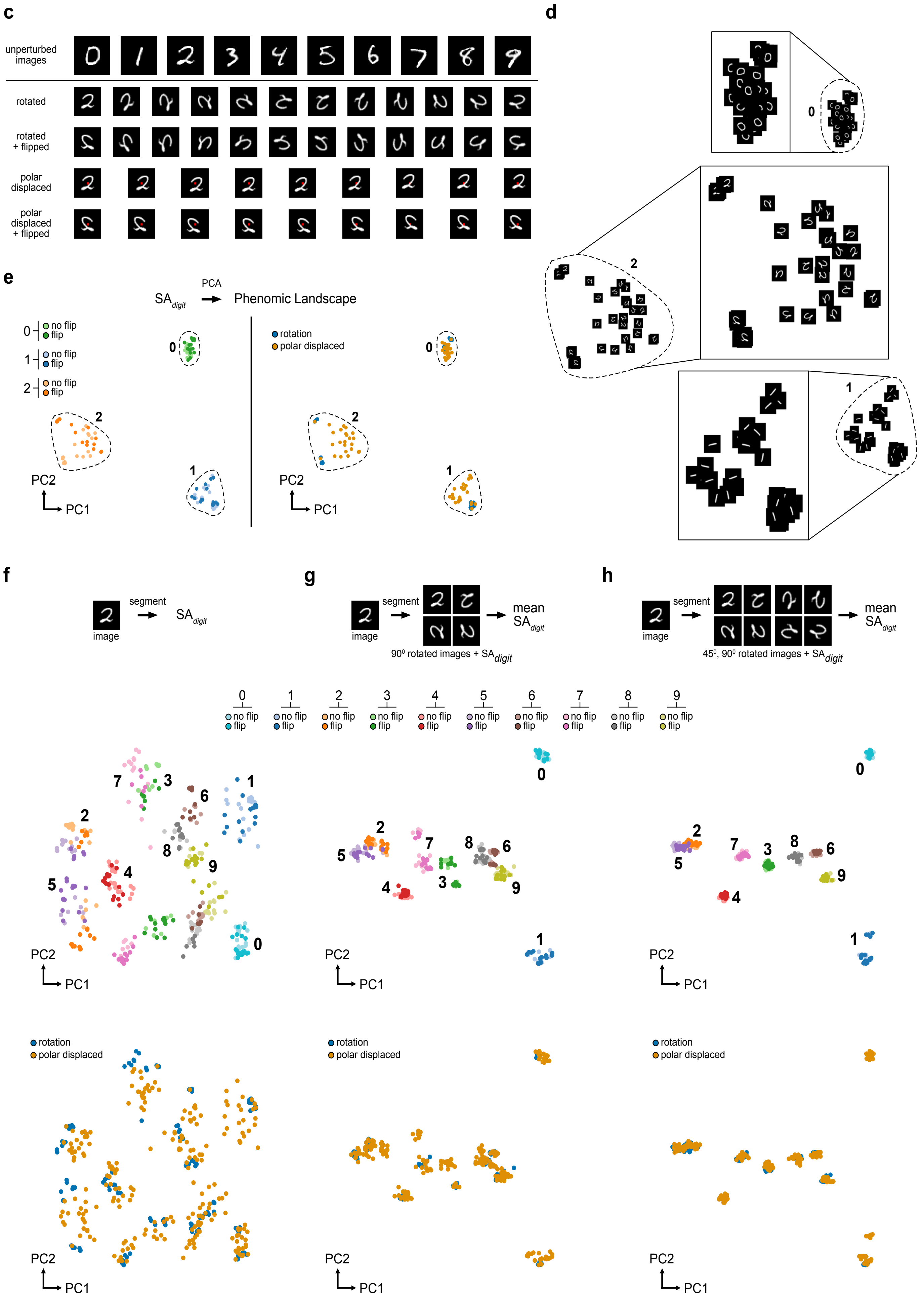
c
unperturbed images
rotated
rotated + flipped
polar displaced
polar displaced + flipped
d
e
SA digit
PCA
Phenomic Landscape
no flip
flip
rotation
polar displaced
PC2
PC1
f
segment
image
SA digit
g
90° rotated images + SA digit
mean SA digit
h
45°, 90° rotated images + SA digit
mean SA digit

Supplementary Figure 1 contd. Assessment of the invariance properties of the shape-appearance (SA) phenome. c) Single image exemplars for the digits 0-9, and resultant images of applying rotation, left-right flipping and polar displacement to the digit 2. d) Principal component analysis (PCA) embedding of the shape, appearance (SA) phenomes of rotated, left-right flipped and polar displaced images of the digits 0,1,2 (12 rotation angles, 9 polar displacements, and flipped variant, n=126 instances). PC plot of the same points colored by unique digit and flipped status (left panel) and by rotational or polar displaced status (right panel). The two plots separated by a solid black line. e) Corresponding digit images plotted at the PCA coordinates in d) with zoom-ins. PCA analysis of all ten digits using the f) individual input image SA phenomes as-in b); or the mean SA phenome averaging all individual SA phenomes of g) all 900 increment rotated versions of the input image, and h) all 450 increment rotated version of the input image as depicted. Panels in f)-h) are organized column-wise with a shared color legend for unique digits. Points colored in f)-h) by unique digit and flipped status (upper panel) and by rotational or polar displaced status (lower panel). The greater the invariance of features, the greater all transformations of the same digit collapse to a single point in the PCA embedding.

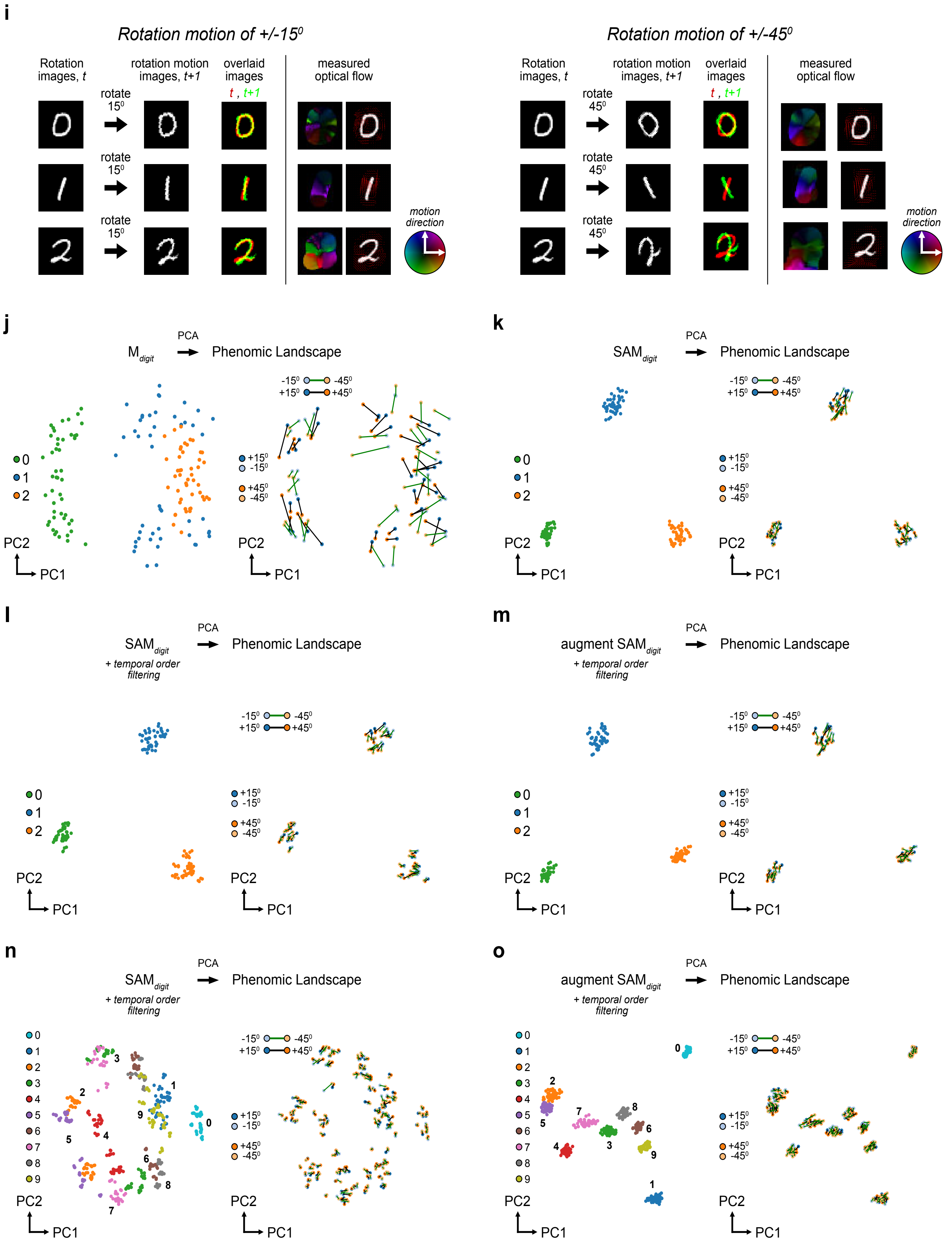

Supplementary Figure 1 contd. Assessment of the invariance properties of the combined shape, appearance, motion (SAM) phenome. i) Representative examples of applying a 150 (left panel) and 450 (right panel) rotation as motion to digits 0,1,2, and the computed optical flow between the non-rotated and rotated image. j) Principal component analysis (PCA) embedding of motion (M) only phenome for digits 0,1,2 rotated and subject to ±150 (left panel) and ±450 (right panel) motion rotations (12 rotations per digit, n=144 instances). PCA embedding of the same data

in j) using k) the combined shape, appearance and motion (SAM) phenomes; l) additionally using temporal order filtering, regarding the rotated versions of a digit as part of a timeseries; and m) additionally using the augment SAM phenomes by concatenating the mean SA phenomes using 900 rotations of each input (see Supplementary Figure 1g) to the M phenome. n), o) PCA analysis of all ten digits using the same phenomes as l) and m) respectively. In j)-o) left plot color points by unique digit identity, right plot color digits by rotation motion and links the same digit image subject to -150 and -450 rotation motion (green line), and the same image subject to +150 and +450 rotation motion (black line). We expect these links to align in the same general orientation in the PCA across digits.

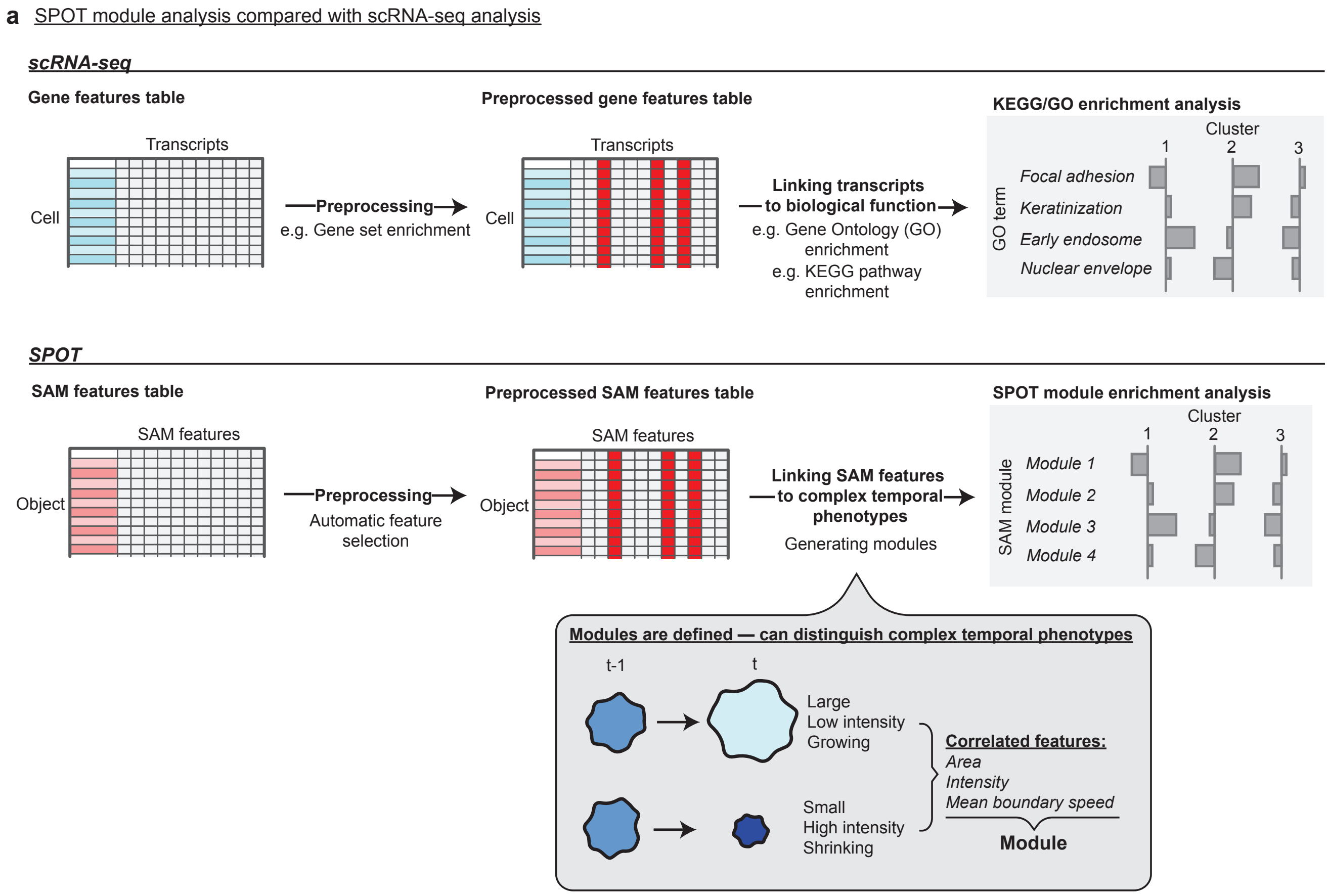

Supplementary Figure 2. SPOT module analysis is analogous to enrichment analysis after scRNA-seq. a). In scRNA-seq analysis, the expression scores of the filtered and preprocessed transcripts are used to identify enrichment of biological pathways via gene ontology (GO) or KEGG pathway enrichment. Similarly, SPOT module analysis hierarchically clusters features in the filtered and preprocessed SAM phenome to first identify modules and then perform module enrichment. Each module represents a more conceptually higher-level complex phenotype than any single feature. Module enrichment analysis scores the ‘expression’ level of each module within a given grouping of object instances.

b Schematic of SAM module analysis

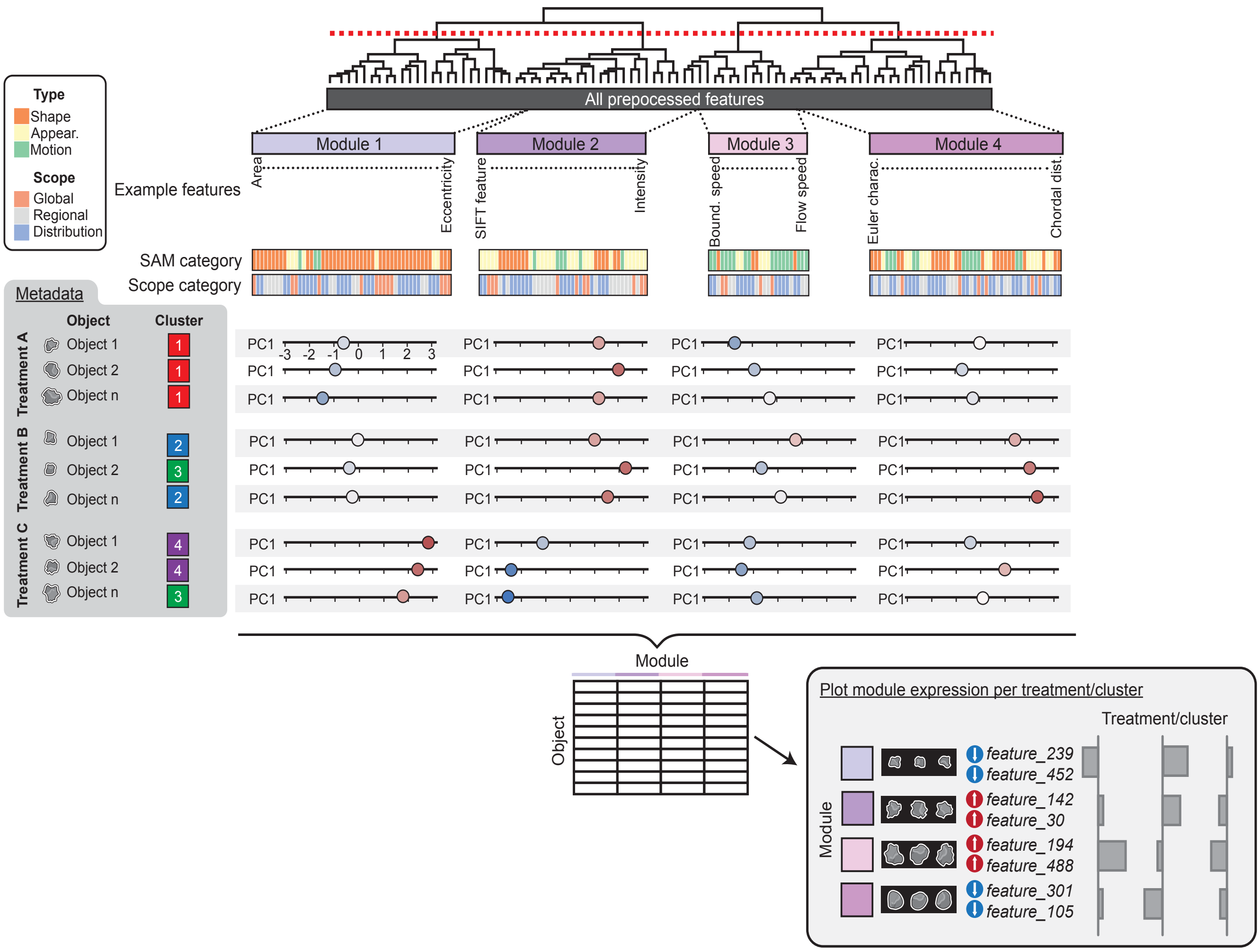

c Schematic of SAM contribution calculation

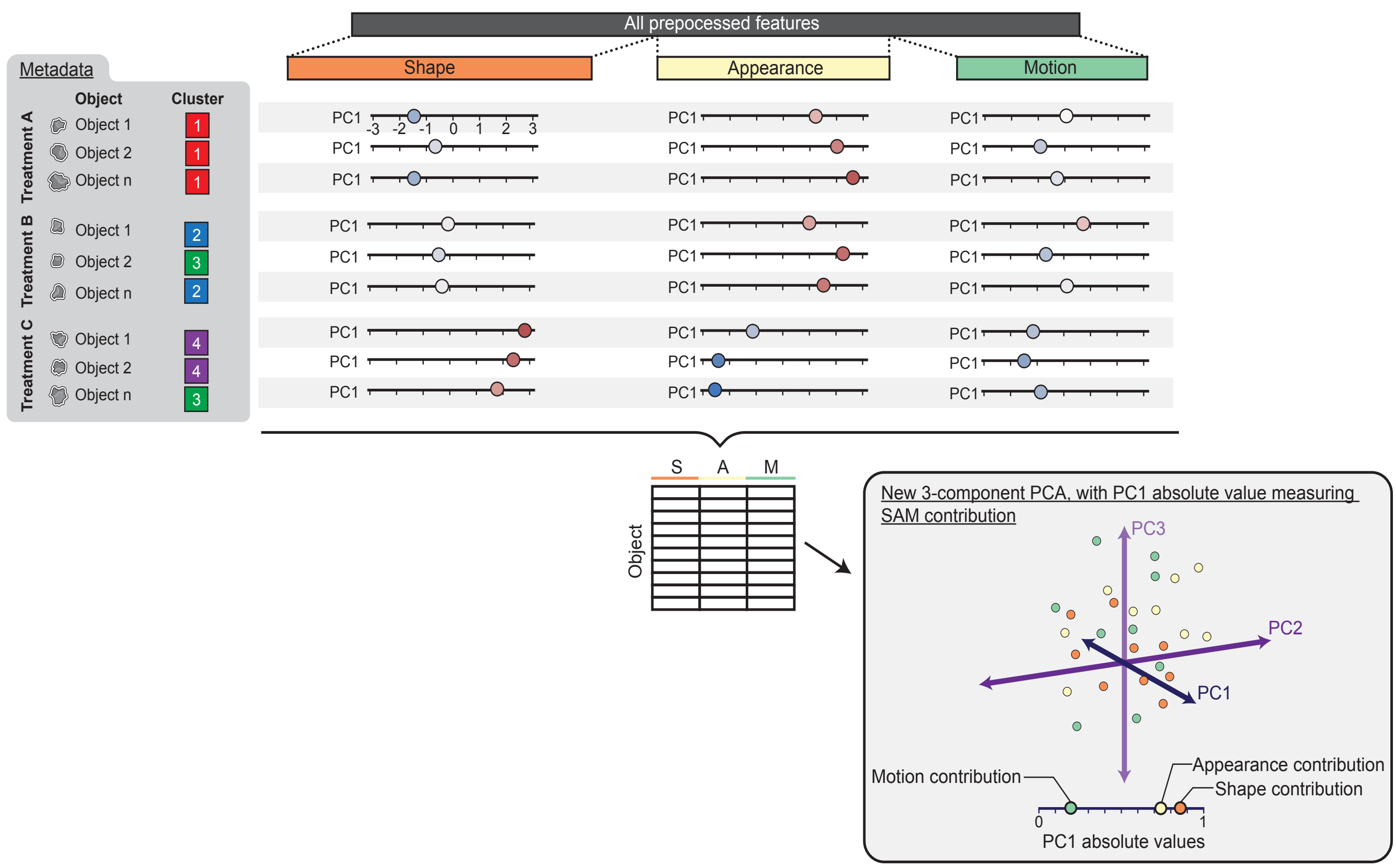

Supplementary Figure 2 cont. Schematic of SPOT module analysis and computation of SAM/scope contributions. b) Identification of SAM modules, groupings of similarly covarying single SAM features by hierarchical clustering. Example SAM and scope categories are displayed for each module, illustrating that correlated features within a module comprise diverse SAM and scope categorizations. A module represents a complex SAM phenotype that cannot be captured by any single feature. For each module, an expression score is generated by calculating the first principal component (PC1) score for each object instance (Methods). The PC1 score measures the contribution of an object instance to the axis of maximal variance, so a high positive PC1 score for a given object instance indicates that its module feature values align closely with PC1. The PC1 expression score can be both positive and negative. A high positive expression indicates the module contributes significantly to the overall variance of the object instances in the dataset, and an enrichment of the complex phenotype the module represents. A high negative expression also indicates a signific nt contribution of the module, but the object instances in the dataset display the inverse of the complex phenotype the module represents e.g. if the module represents an elongated shape phenotype, the inverse phenotype is a spherical shape. Individual modules are interpreted by finding representative exemplar object instances and top driving single features (Methods). Module expression can be computed for any grouping of object instances by experimental condition, timepoint, or SAM phenotype cluster for comparative analysis. c) Shape, appearance, and motion contribution scores can be generated by individually subdividing the features into their SAM categories which serve as modules, so that for each category, we compute the PC1 scores as the category expression for all object instances. A PCA is performed with the category expression to determine the contribution of each category to the first principal component axis. The absolute value of the contribution is defin d as the contribution of the SAM category on a scale of 0 to 1, (Methods).

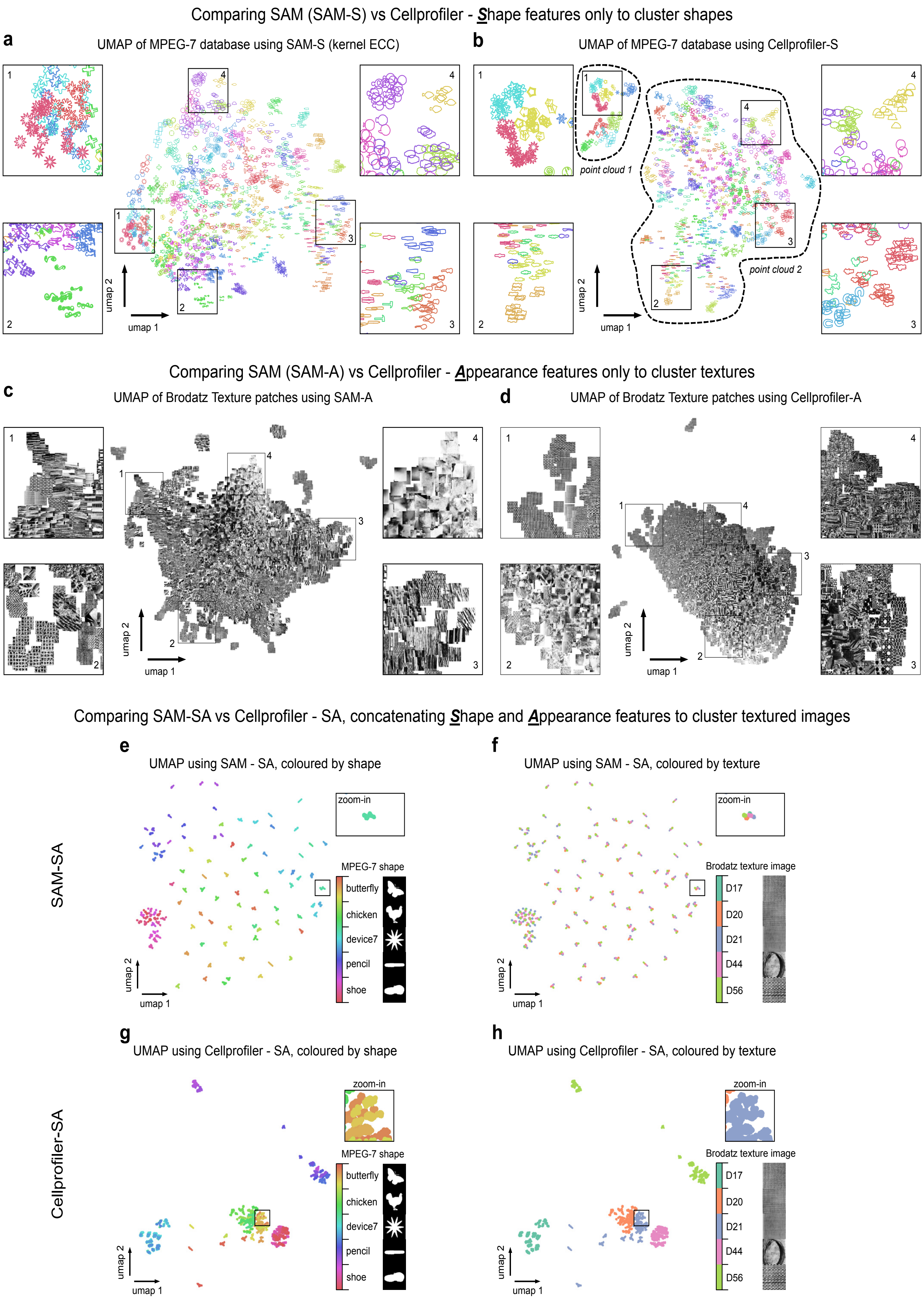
Comparing SAM (SAM-S) vs Cellprofiler - Shape features only to cluster shapes
a
UMAP of MPEG-7 database using SAM-S (kernel ECC)
b
UMAP of MPEG-7 database using Cellprofiler-S
point cloud 1
point cloud 2
umap 1
umap 2
Comparing SAM (SAM-A) vs Cellprofiler - Appearance features only to cluster textures
c
UMAP of Brodatz Texture patches using SAM-A
d
UMAP of Brodatz Texture patches using Cellprofiler-A
Comparing SAM-SA vs Cellprofiler - SA, concatenating Shape and Appearance features to cluster textured images
e
UMAP using SAM - SA, coloured by shape
f
UMAP using SAM - SA, coloured by texture
g
UMAP using Cellprofiler - SA, coloured by shape
h
UMAP using Cellprofiler - SA, coloured by texture
SAM-SA
Cellprofiler-SA
zoom-in
MPEG-7 shape
butterfly
chicken
device7
pencil
shoe
Brodatz texture image
D17
D20
D21
D44
D56

Supplementary Figure 3. Comparison of using SPOT vs CellProfiler computed shape and appearance features to map phenotypic diversity in computer vision datasets. a) Shape phenomic landscape constructed by applying 2D-UMAP to SPOT's shape-related features, SAM-S (kernel ECC), same as in Fig. 3b. b) Shape phenomic landscape constructed by applying 2D-UMAP to CellProfil r's shape-related features. Each point is an individual shape image in the MPEG-7 dataset colored by its shape category. Similar shapes colocalize to the same local region of the landscape as shown by zoomed-in images 1-4 but are less distinctly separated compared to SAM-S in (a). Moreover, the landscape comprises two point-clouds, indicated by dash black contour lines. c) Appearance phenomic landscape constructed by applying 2D-UMAP to SPOT's appearance-related features, SAM-A, same as in Fig. 3d. d) Appearance phenomic landscape constructed by applying 2D-UMAP to CellProfiler-A, CellProfile 's set of exclusively appearance-related features. Each shape is a 64x64 image patch cropped from the original 512x512 112 Normalized Brodatz texture images. Images cropped from the same original Brodatz texture image and primarily images with the same intensity hue appear to the same local region of the landscape as shown by zoomed-in images 1-4. This contrasts with SAM-A in (c) which organizes primarily by texture. e,f) Shape-appearance phenomic landscape constructed by applying 2D-UMAP to the concatenated SAM-S and SAM-A feature sets of the textured shapes created from five selected MPEG-7 shape categories (Fig. 3e) and five randomly selected Normalized Brodatz texture images. Each point is a 128x128 image colored by e) its source MPEG-7 shape category and f) source Brodatz texture. g,h) Shape-appearance phenomic landscape for concatenated CellProfiler-S and CellProfiler- applied to the same dataset as e,f), colored by g) its source MPEG-7 shape category and g) source Brodatz texture.

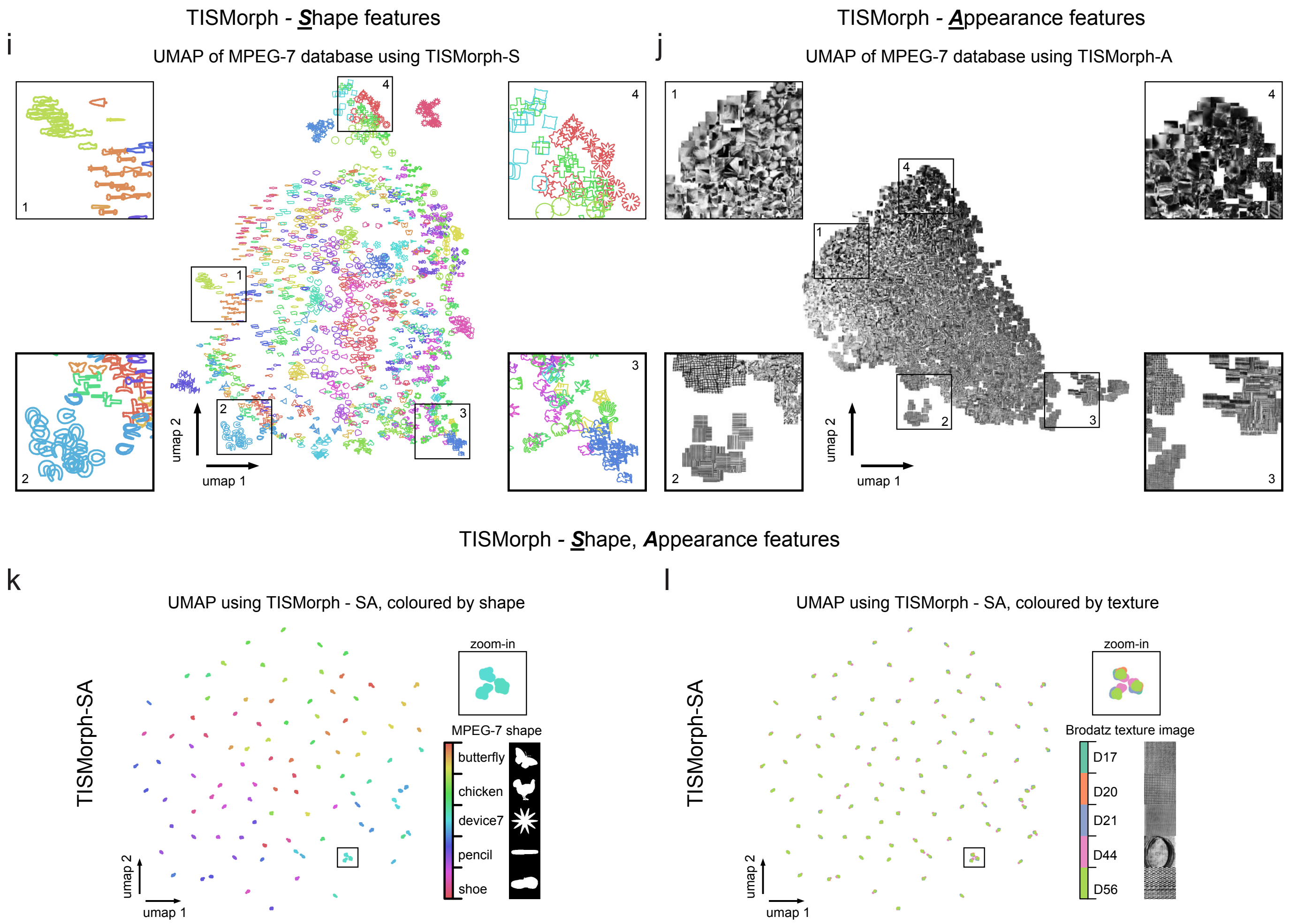

Supplementary Figure 3 cont. TISMorph computed shape and appearance features to map phenotypic diversity in computer vision datasets. i) Shape phenomic landscape constructed by applying 2D UMAP to TISMorph's shape-related features (TISMorph-S) in the MPEG-7 shape dataset. j) Appearance phenomic landscape constructed by applying 2D UMAP to TISMorph's appearance-related features (TISMorph-A) in the Normalized Brodatz texture image dataset. k,l) Shape-appearance phenomic landscape constructed by applying 2D UMAP to the concatenated TISMorph-S and TISMorph-A feature sets in the textured MPEG-7 shape dataset we created.

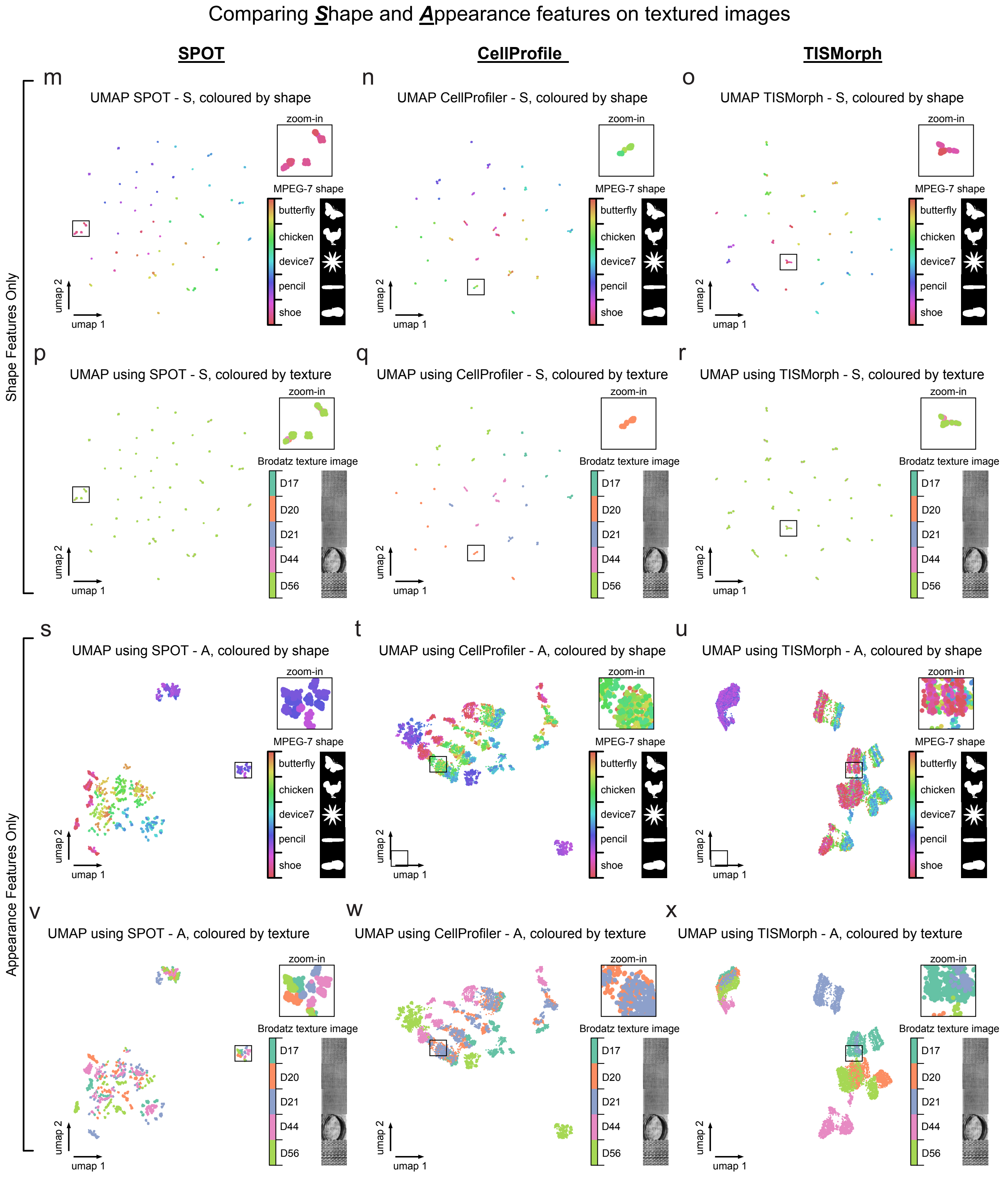

Supplementary Figure 3 cont. Comparison of specific information captured by shape and appearance features in SPOT, TISMorph and CellProfiler features on Brodatz textured MPEG7 shapes. m-o) Shape phenomic landscape constructed by applying 2D UMAP to the shape-related features of SPOT, Cellprofiler and TISmorph (left to right) coloured by MPEG-7 shape class. p-r) same phenomic landscape as in m-o) coloured by Brodatz texture class. s-u) Appearance phenomic landscape constructed by applying 2D UMAP to the appearance-related features of SPOT, Cellprofi er and TISmorph (left to right) coloured by MPEG-7 shape class. v-x) same phenomic landscape as in s-u) coloured by Brodatz texture class. An ideal set of shape features should cluster individual shape classes but show no discrimination for texture. Likewise, an ideal set of appearance features should cluster individual texture classes but show no discrimination for shape.

a

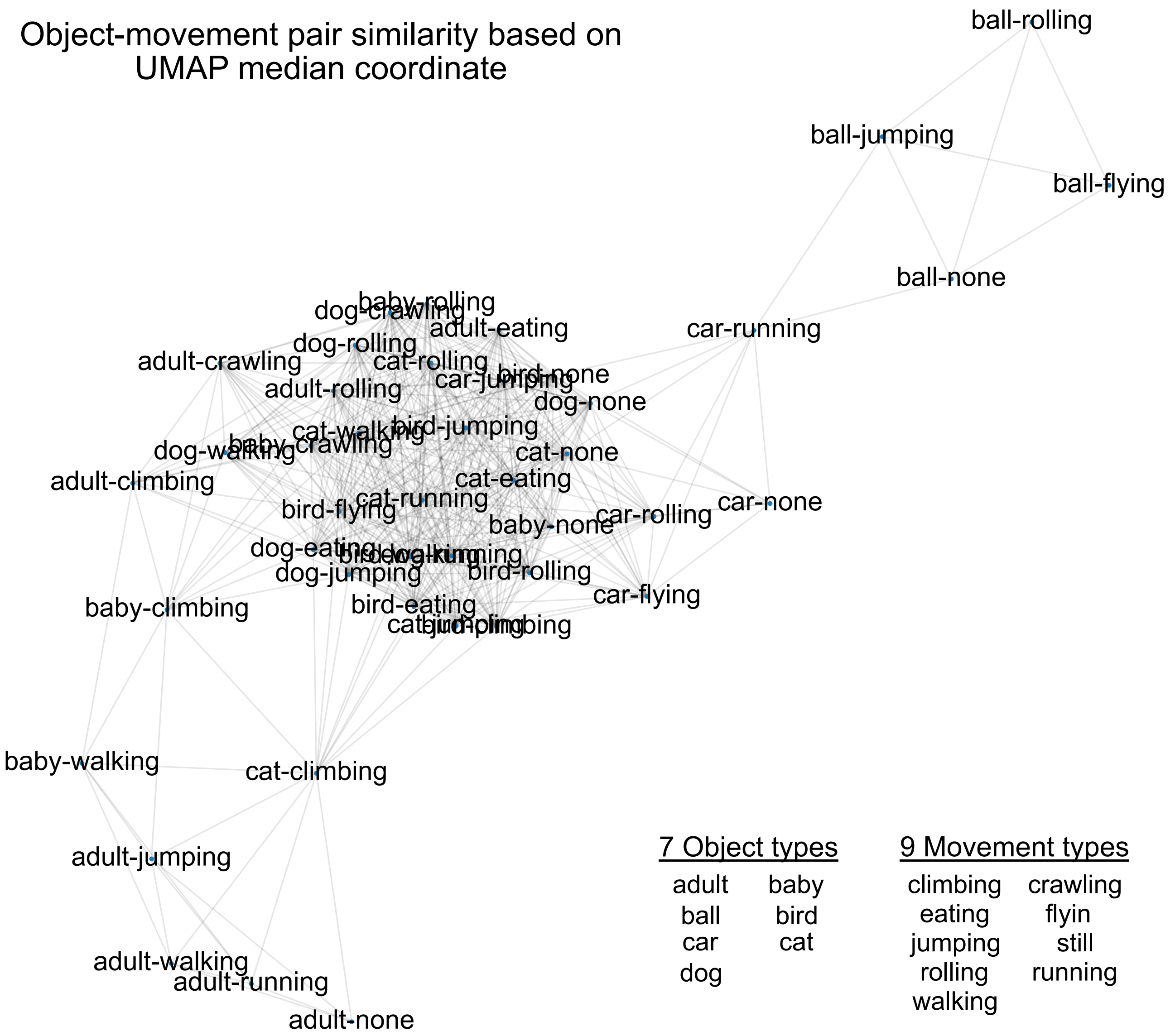

Supplementary Figure 4. Similarity graph between the 43 unique object-movement classes of the A2D dataset. a). Graph computed using the pairwise distance between the median 2D-UMAP phenomic landscape coordinates of each unique object-movement class pairing, (Methods). Visualization uses a Kamada-Kawai force-directed layout initialized with the median UMAP coordinates[86]. None refers to the still action type.

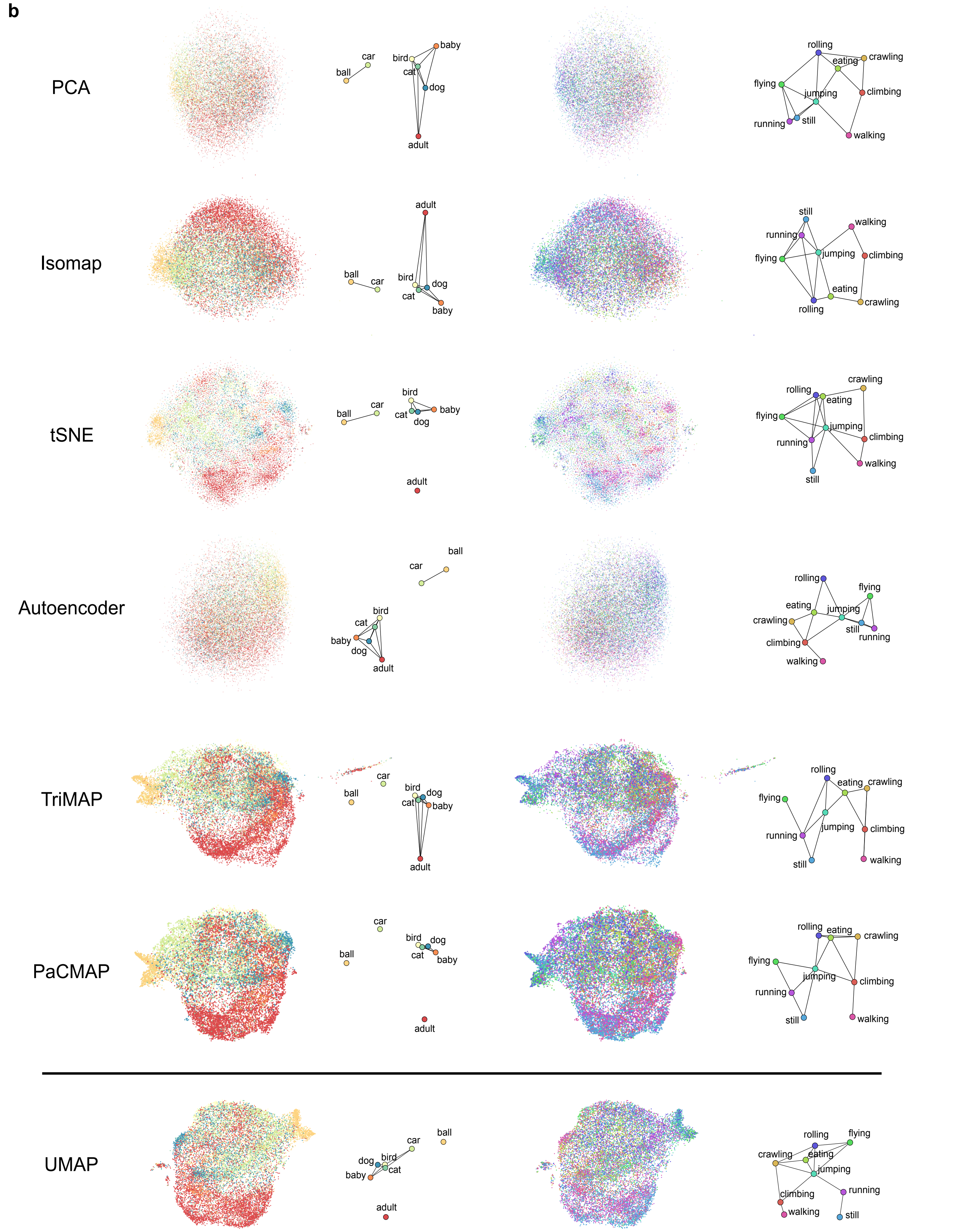

Supplementary Figure 4 cont. Comparison of different dimensionality reduction techniques and resultant similarity graphs of the seven unique object and nine unique movement classes of the A2D dataset. b). Computed 2D phenomic landscape with each indicated dimensionality reduction method. Each individual point is an object and is colored by object (first column) and

movement (third column) class. Object (second column) and movement (fourth column) similarity graphs were computed using the pairwise distance between the median 2D phenomic landscape coordinates of each dimensionality reduction method (Methods). Graphs were visualized using a Kamada-Kawai force-directed layout initialized with the median UMAP coordinates[86].

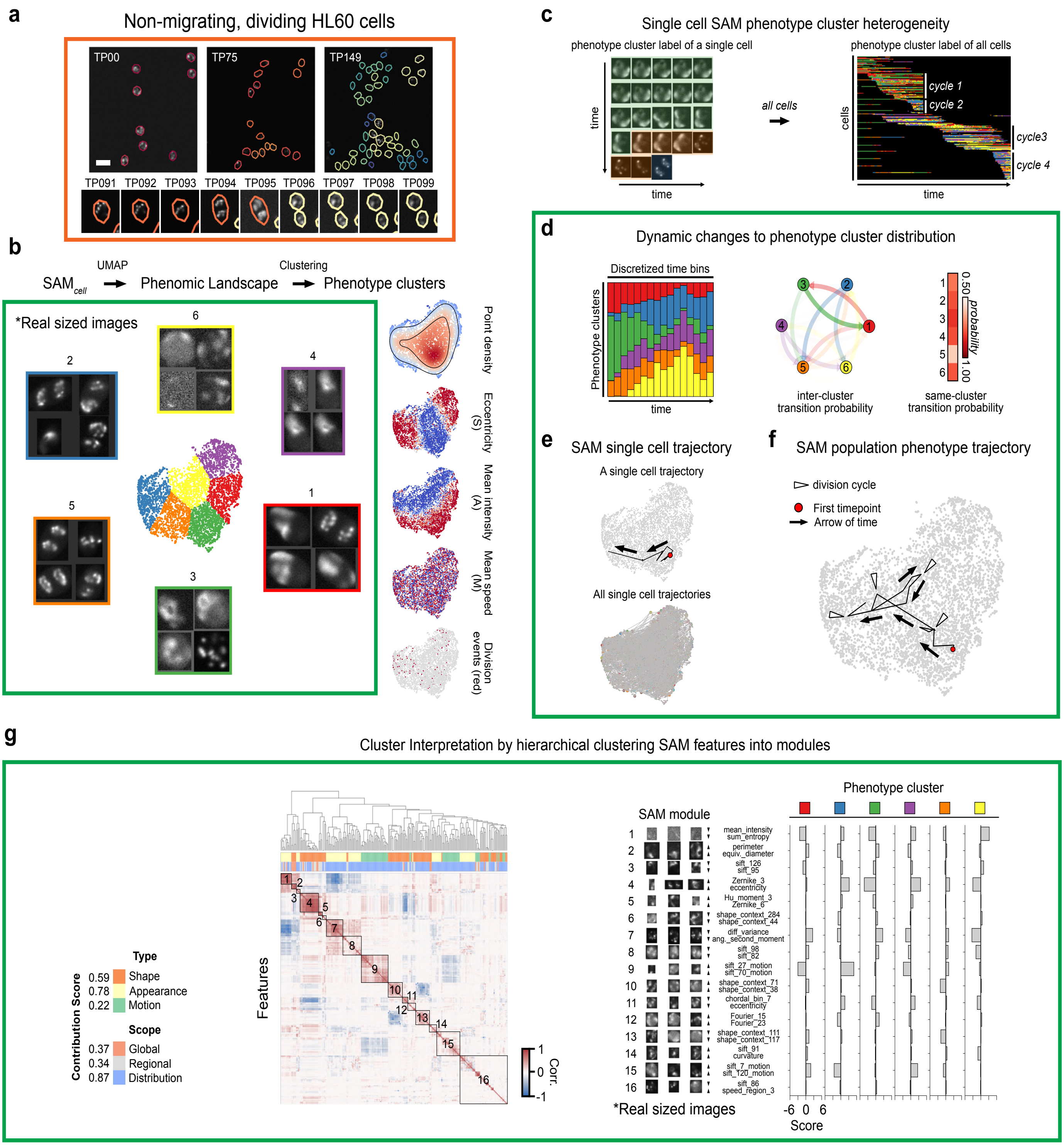

Supplementary Figure 5. SPOT characterizes phenotypic heterogeneity of single cell division. a) Snapshot of the full field-of-view at timepoint 0, 75 and 149 of simulated HL60 cell nuclei with the boundary of each nucleus uniquely colored (top), and snapshots of select cells before or after cell division (bottom). The videos show majority cell division with little migration. Each timepoint is a video frame and is 29 min. Scalebars: 10 µm. b) SAM phenomic landscape constructed as in Figure 5b. c) Mapping a single cell tracked over time (left) and all continuously tracked cells (right) into the SAM phenomic landscape of b) and coloring each temporal instance by the corresponding phenotype cluster. d) Stacked barplot showing the relative frequency of each phenotype cluster over discrete time bins (left). Graph showing the next timepoint relative transition probability to another phenotype cluster (centre), and heatmap showing the probability of staying in the same phenotype cluster (right). Arrows colored by the source cluster. The more transparent the arrow the smaller the probability of transition. e) Single SAM phenotype trajectory summarizing the temporal phenotype

dynamics of a single HL60 cell (top) and the SAM phenotype trajectory of all cells (bottom) with starting timepoint colored red. Black arrow shows the directionality of time. f) Single population-level SAM phenotype trajectory summarizing the temporal evolution and phenotypic diversity across all cells. Starting timepoint is colored red. Black arrows show the directionality of time. White arrows depict a cell division cycle. g) Contribution score of shape, appearance and motion, and global, regional and distributional features in explaining the dataset variance defined as the absolute value of the first principal component (left). Automated hierarchical clustering of the covariation between SAM features to identify principal SAM feature modules (middle, modules outlined and numbered along the diagonal). Expression of each SAM module (labeled 'score' in barplot) in each phenotype cluster (right). Each module is depicted with their top three most representative images, its top driving SAM features and whether each feature is enriched (up arrow) or depleted (down arrow).

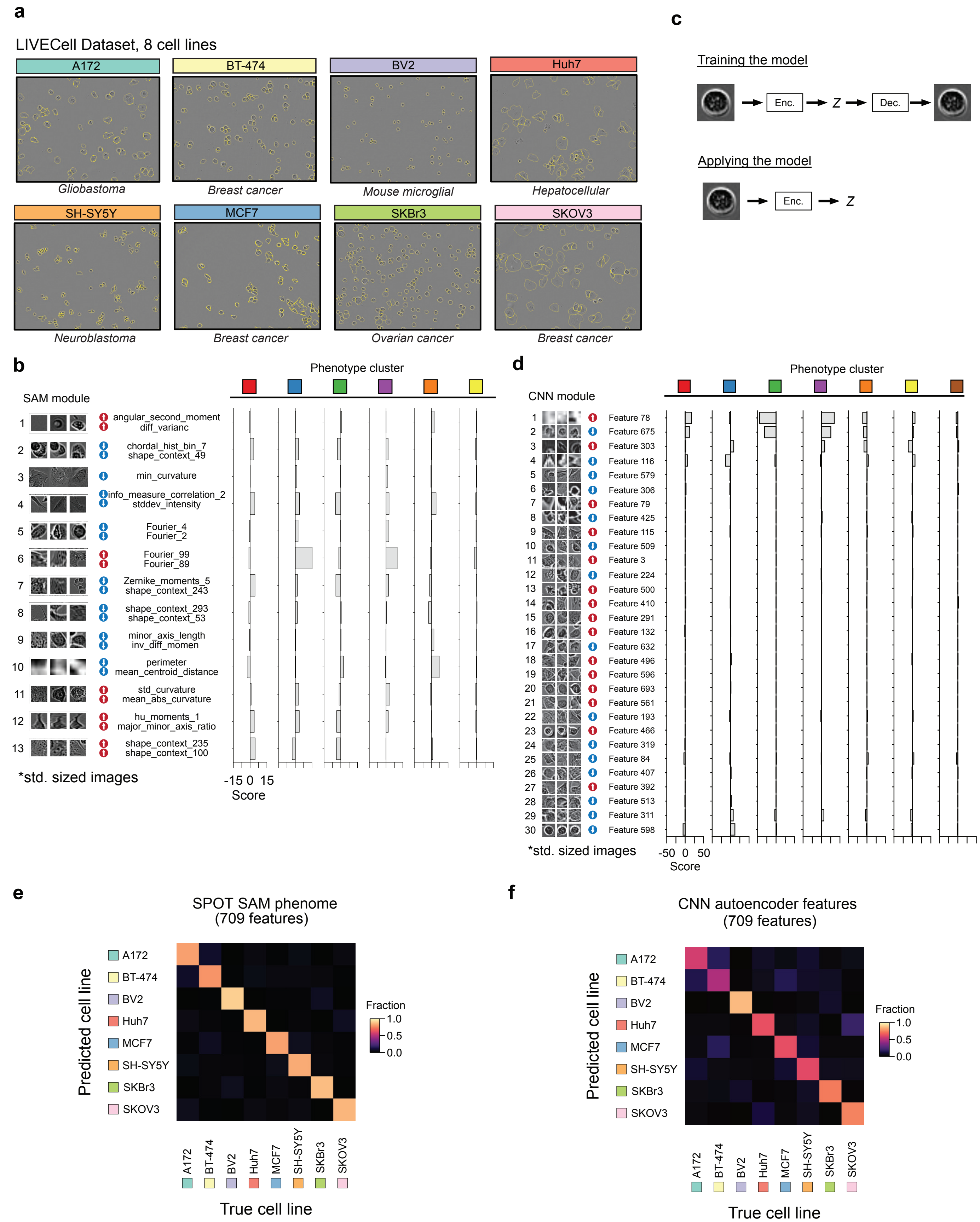

Supplementary Figure 6. SPOT analysis of LIVECell dataset using SAM and CNN autoencoder features. a) Example phase-contrast images, with provided manual cell outlines (yellow) for each of the 8 cell lines in the LIVECell dataset. b) Schematic of training a convolutional neural network autoencoder to extract features from image crops (resized to standard size, 64 x 64 pixels same

as that used in SPOT analysis) using reconstruction (top), and then applying the trained encoder to extract features (bottom). c,d) Expression of each module (labeled as ‘score’ in barplots, Methods) in each phenotype cluster following hierarchical clustering of c) SAM and d) CNN features into modules. Each module is depicted with its three selected most representative images, its top driving features, and whether the feature is enriched (up arrow) or depleted (down arrow). e) Confusion matrix using the SPOT filtered SAM phenome after preprocessing (parameters set by the train split) and feature normalization on the test split. f) Confusion matrix using the CNN encoder generated features as-is on the same test split as the SAM phenome, with no feature selection or normalization.

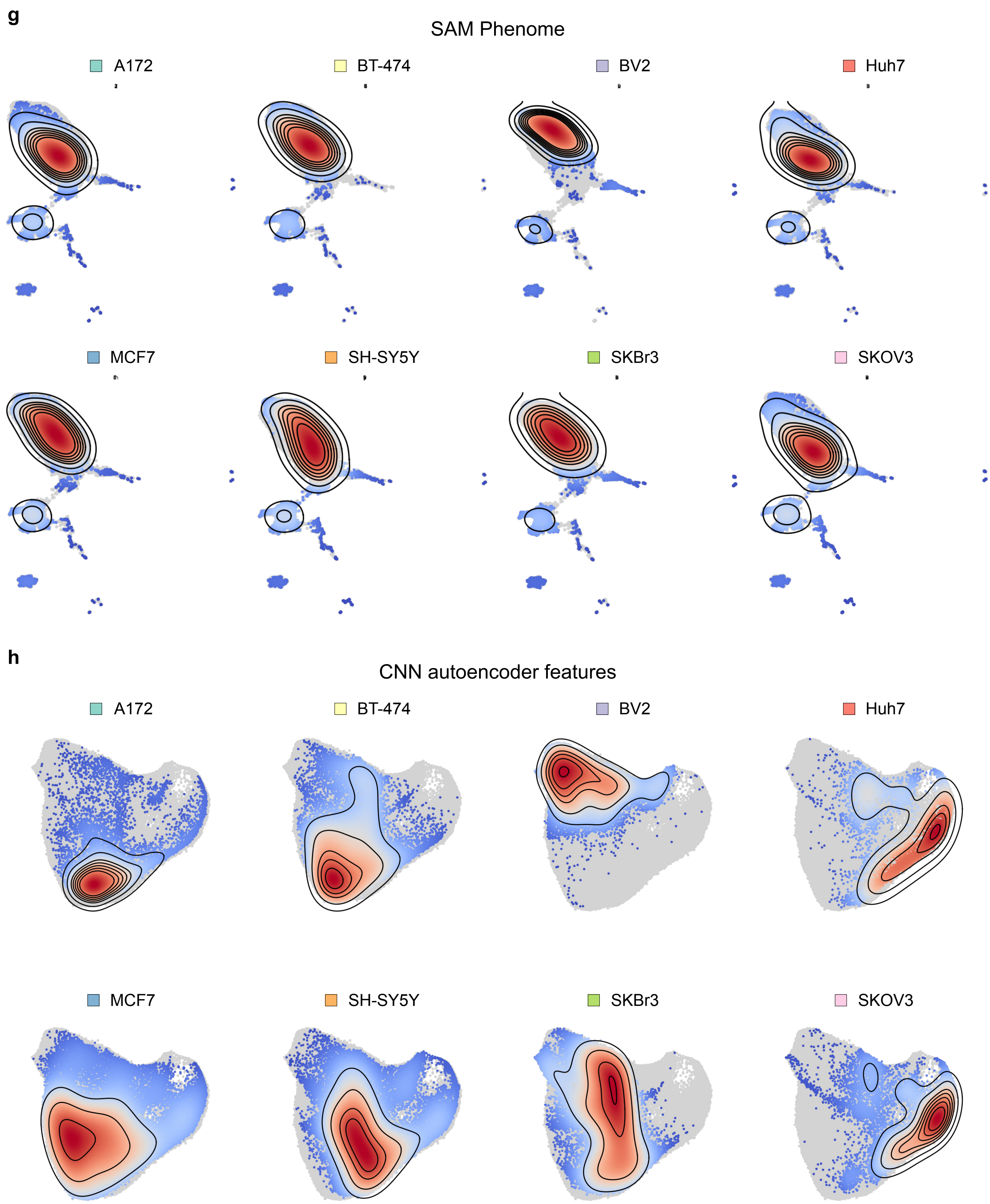

Supplementary Figure 6 cont. Density heatmap of individual cell lines when mapped into the SAM phenome or CNN features phenomic landscape. g) Heatmap of the local point density of cells mapped into the phenomic landscape generated by applying UMAP to the SAM phenome. h) Same as a) after applying UMAP to the CNN features. Contour lines depict mean + 1, 2, 3, 3.5, 4, 4.5 and 5 standard deviations of the point density.

i

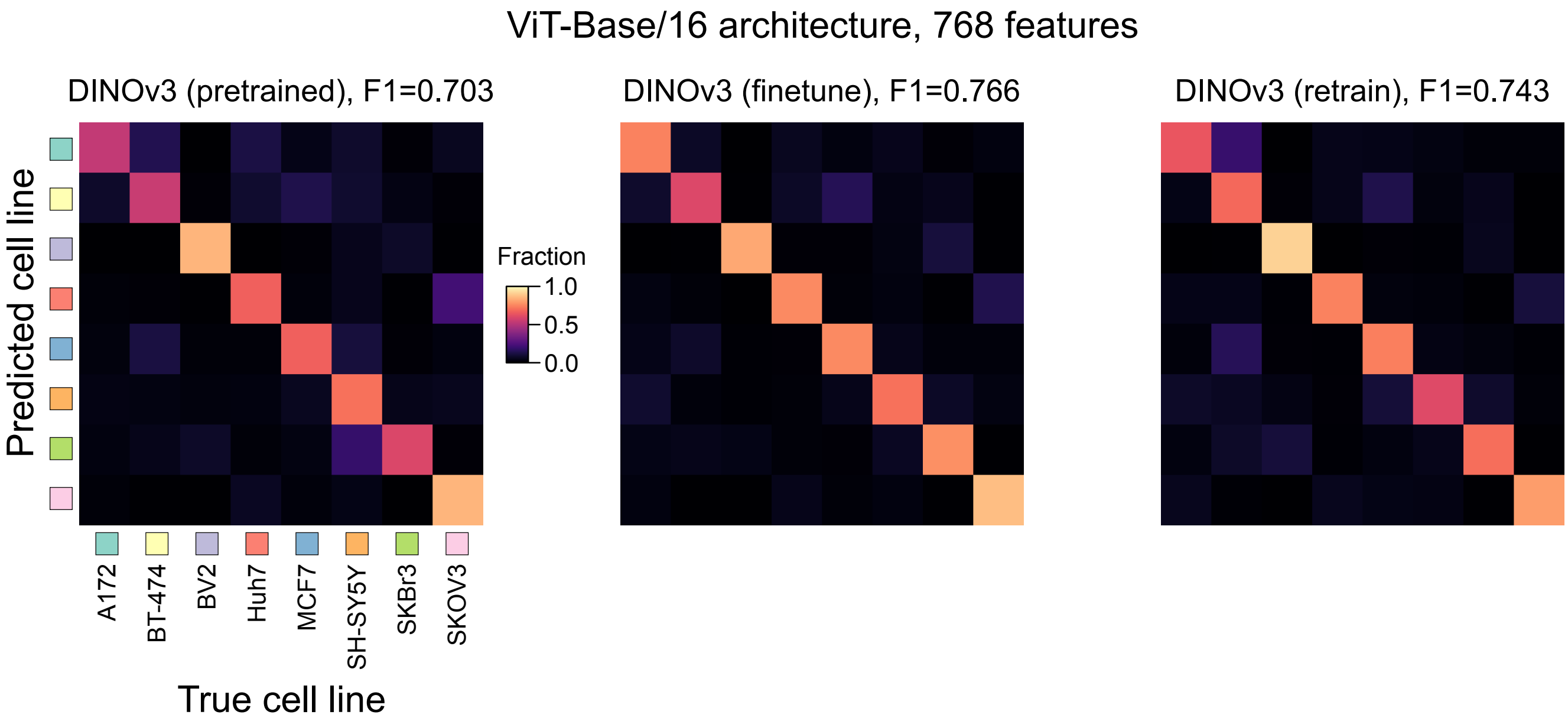

j

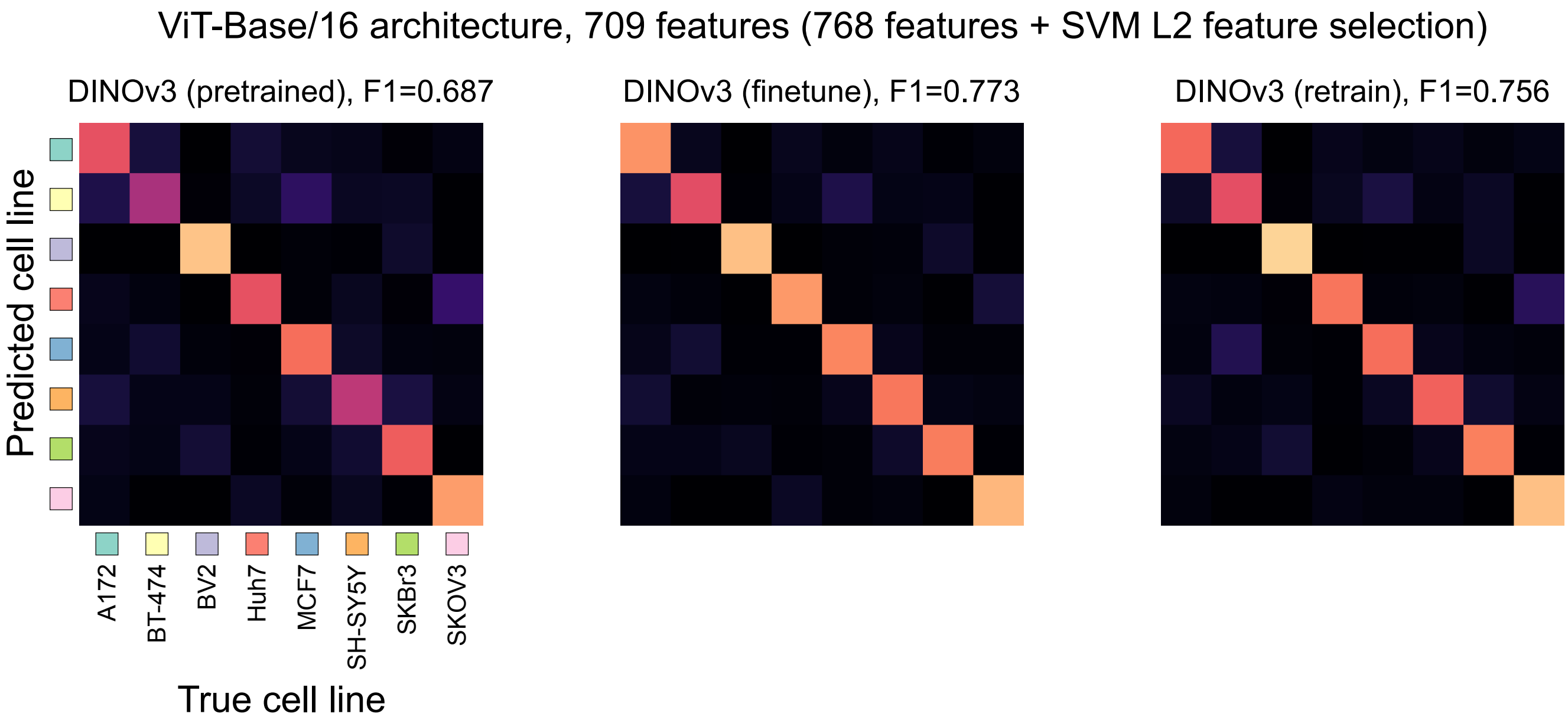

Supplementary Figure 6 cont. Cell line classification performance using self-supervised DINOv3 features. i) Confusion matrix of classification using a pretrained, fine-tuned and retrained DINOv3 model with ViT Base/16 architecture and CLS token dimensionality of 768 (left to right). j) Confusion matrix of the same models in i) after using a linear support vector machine with L2 penalty to rank individual features and select the most relevant 709 features to match the filtered SPOT-SAM phenomes.

k

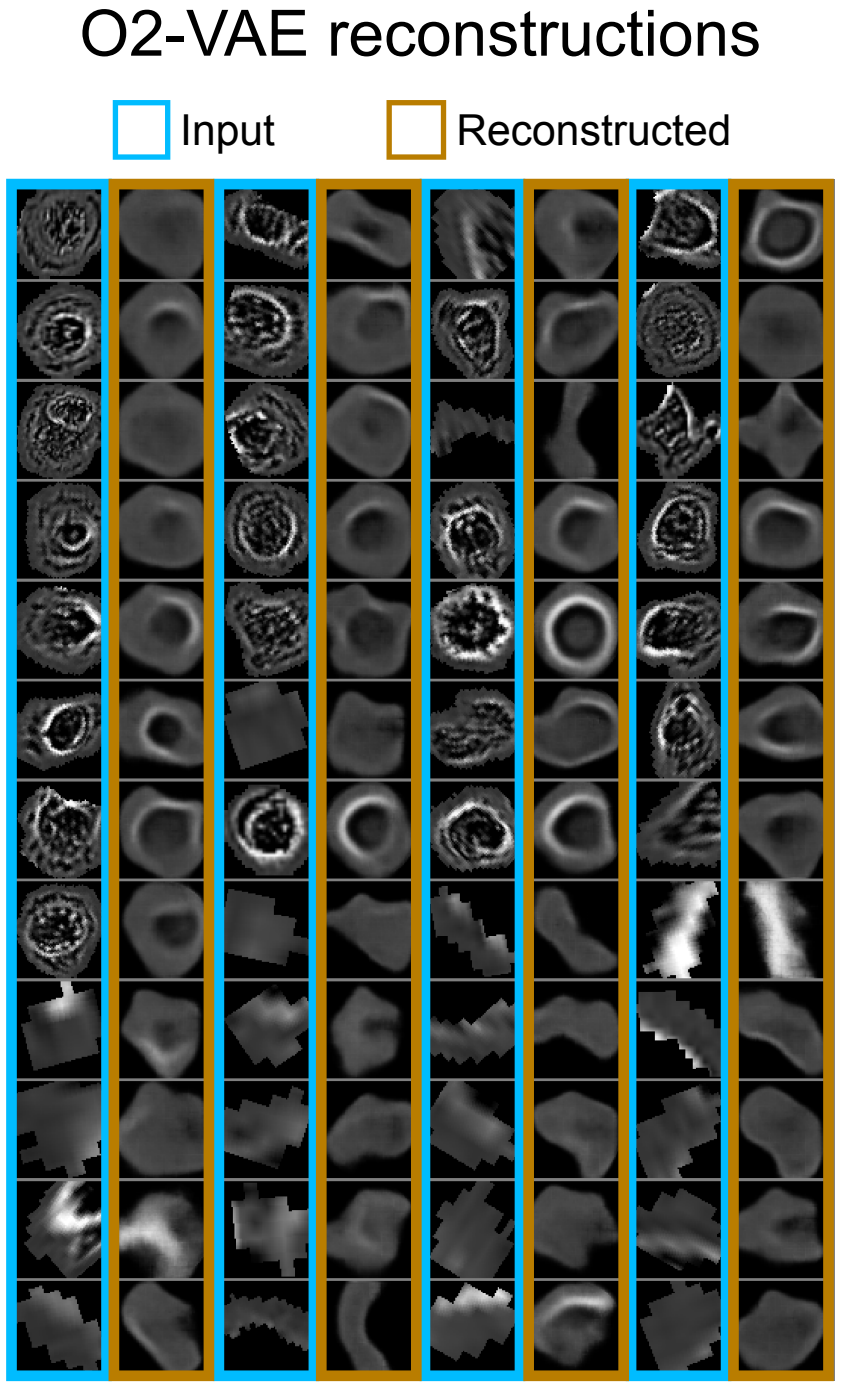

l

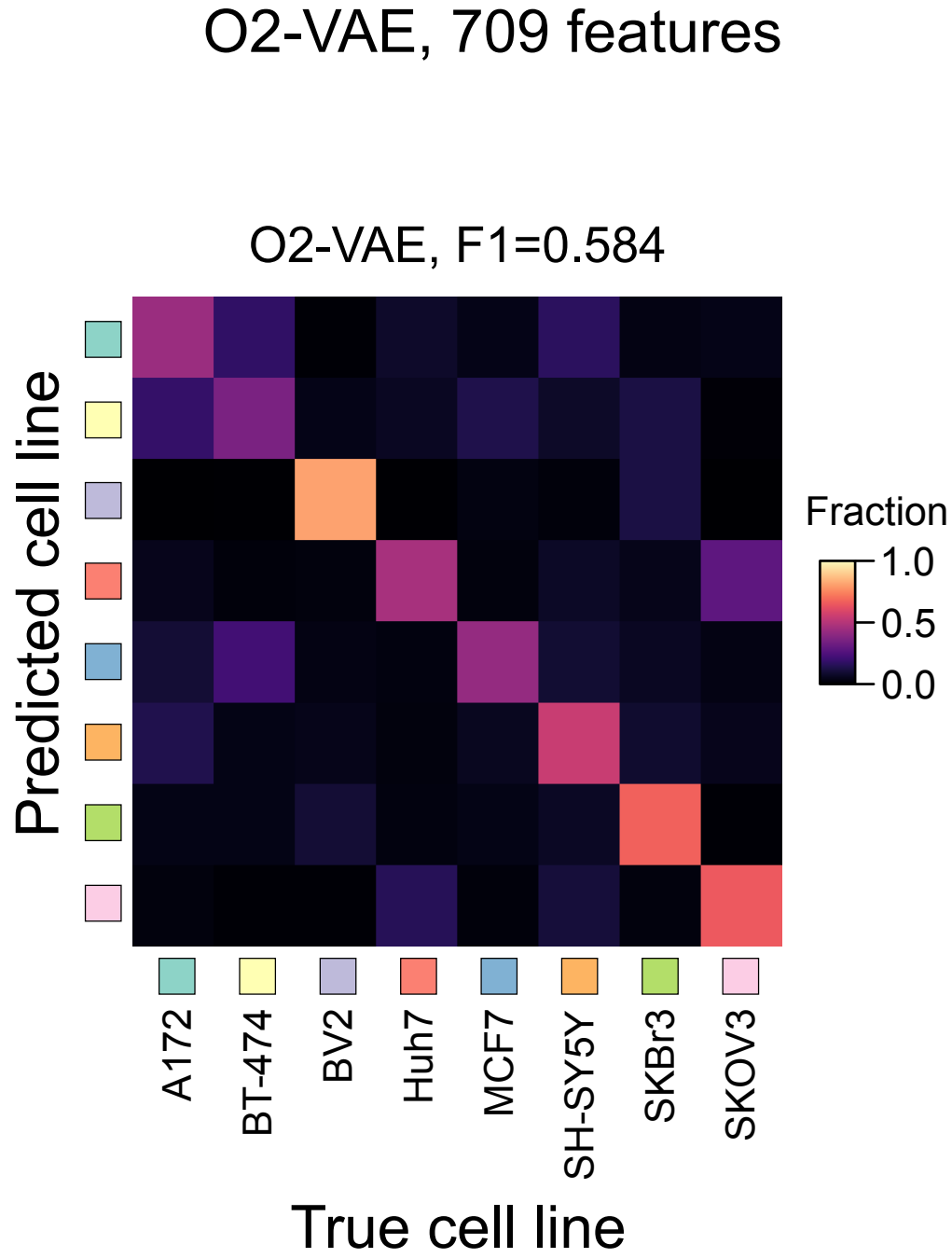

Supplementary Figure 6 cont. Cell line classification performance using self-supervised O2-VAE features. k) Confusion matrix of classific tion using a pretrained, fine-tuned and retrained DINOv3 model with ViT Base/16 architecture and CLS token dimensionality of 768 (left to right). l) Confusion matrix of the same models in a) after using a linear support vector machine with L2 penalty to rank individual features and select the most relevant 709 features to match the filtered SPOT-SAM phenomes.

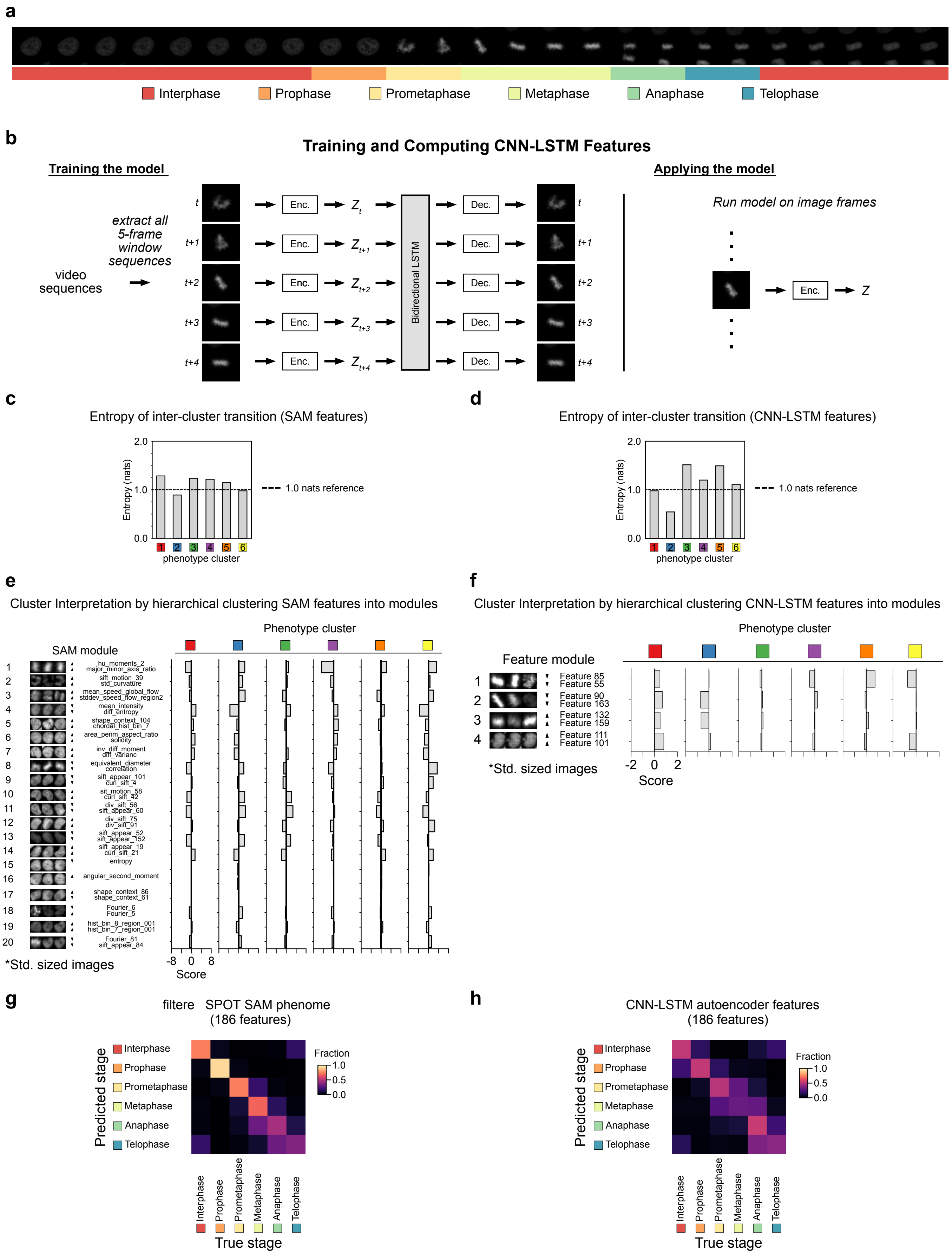

Supplementary Figure 7. SPOT analysis of HeLa cell histone H2B dataset using SAM and CNN-LSTM autoencoder features. a) Representative video from the HeLa cell histone H2B dataset for a single cell, showing the changes in nuclear morphology as the cell divides and the corresponding manually annotated staging, depicted as a colorbar below. A unique color is ascribed to each of the six stages. b) Schematic of training the Convolutional Neural Network – Long Short-Term Memory (CNN-LSTM) autoencoder to learn to extract temporally-informed features trained on 5-frame image crop sequences (each crop resized to standard size, 64 x 64 pixels same as

that used in SPOT analysis) by reconstruction (left), and then applying the trained CNN encoder to extract features from individual single timepoint image crops (right). c), d) Shannon entropy of cluster transition for each phenotype cluster found by SPOT analysis of the SAM phenome or CNN-LSTM features respectively. e) Automated hierarchical clustering of the covariation between SAM features to identify principal SAM feature modules. The expression of each SAM module (labeled as ‘score’ in barplots, Methods) in each phenotype cluster. Each module is depicted with its three selected most representative images, its top driving SAM features and whether the feature is enriched (up arrow) or depleted (down arrow) (see Methods). f) Automated hierarchical clusters of the covariation between CNN-LTSM features to identify principal feature modules, as for e). g) Confusion matrix using the SPOT filtered SAM phenome after preprocessing (parameters set by the train split) and feature normalization on the test split. h) Confusion matrix using the trained CNN-LSTM encoder generated features as-is on the same test split as the SAM phenome, with no feature selection or normalization.

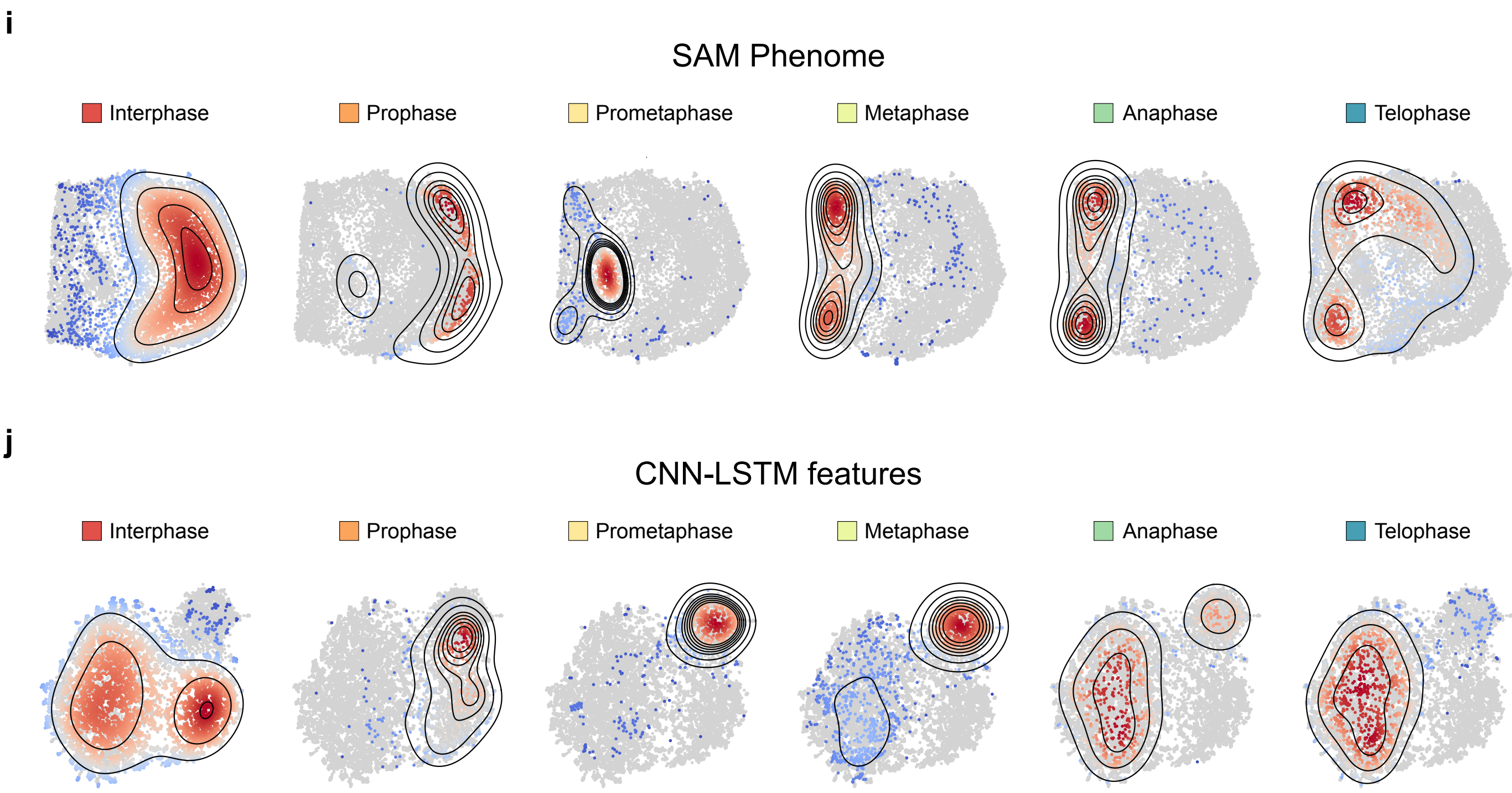

Supplementary Figure 7 cont. Density heatmap of cell division stage when mapped into the SAM phenome or CNN-LSTM features phenomic landscape. i) Heatmap of the local point density of cells mapped into the phenomic landscape generated by applying UMAP to the SAM phenome. j) Same as a) after applying UMAP to the CNN-LSTM features. Contour lines depict mean + 1, 2, 3, 3.5, 4, 4.5 and 5 standard deviations of the point density.

k

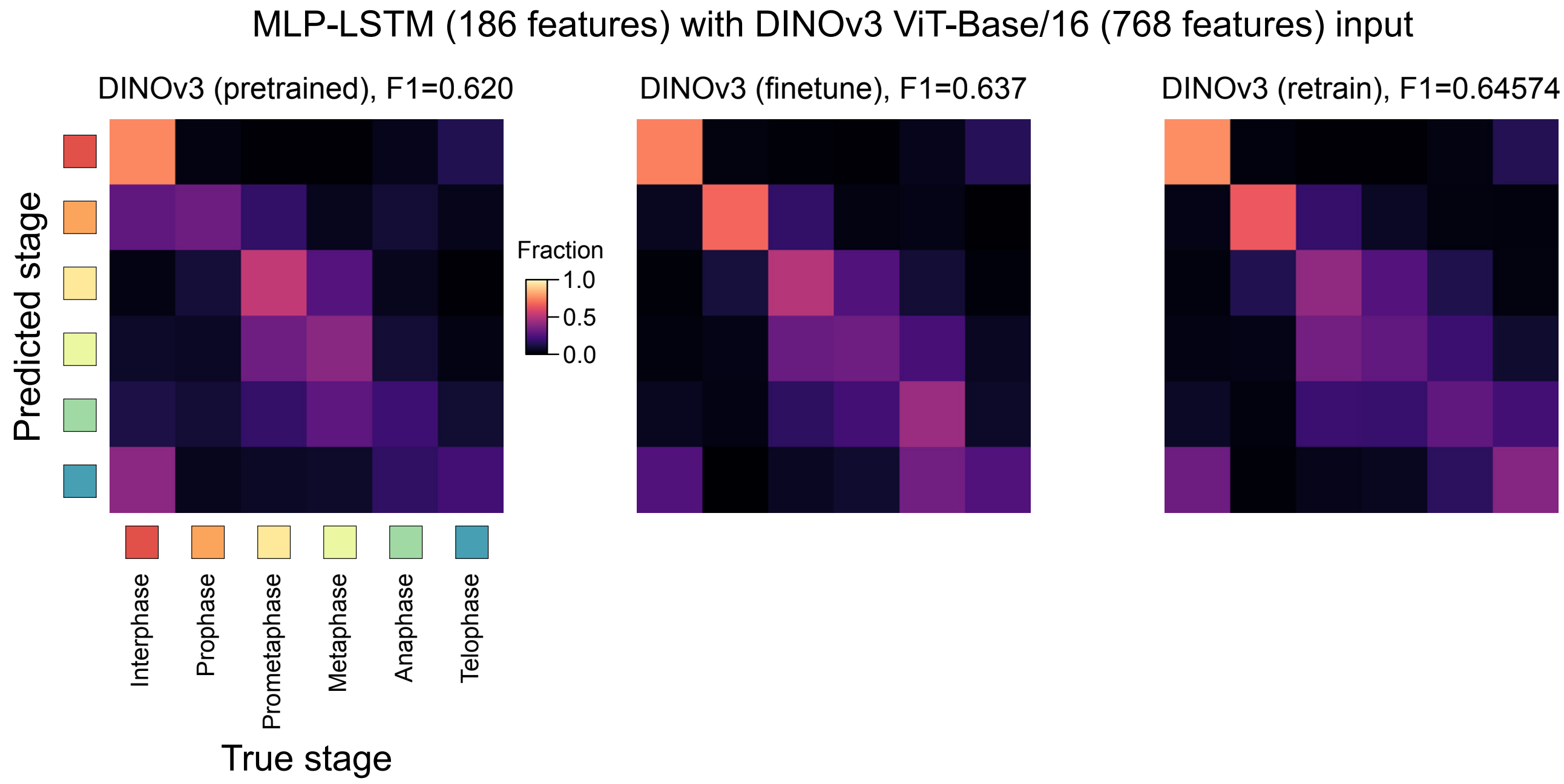

Supplementary Figure 7 cont. Mitosis stage classification performance using self-supervised DINOv3 features. k). Confusion matrix of classification using a MLP-LSTM autoencoder model with 186 features matching the feature number in the filtered SPOT-SAM phenomes, trained on the 768 features generated by a pretrained, fine-tuned and retrained DINOv3 model (left to right).

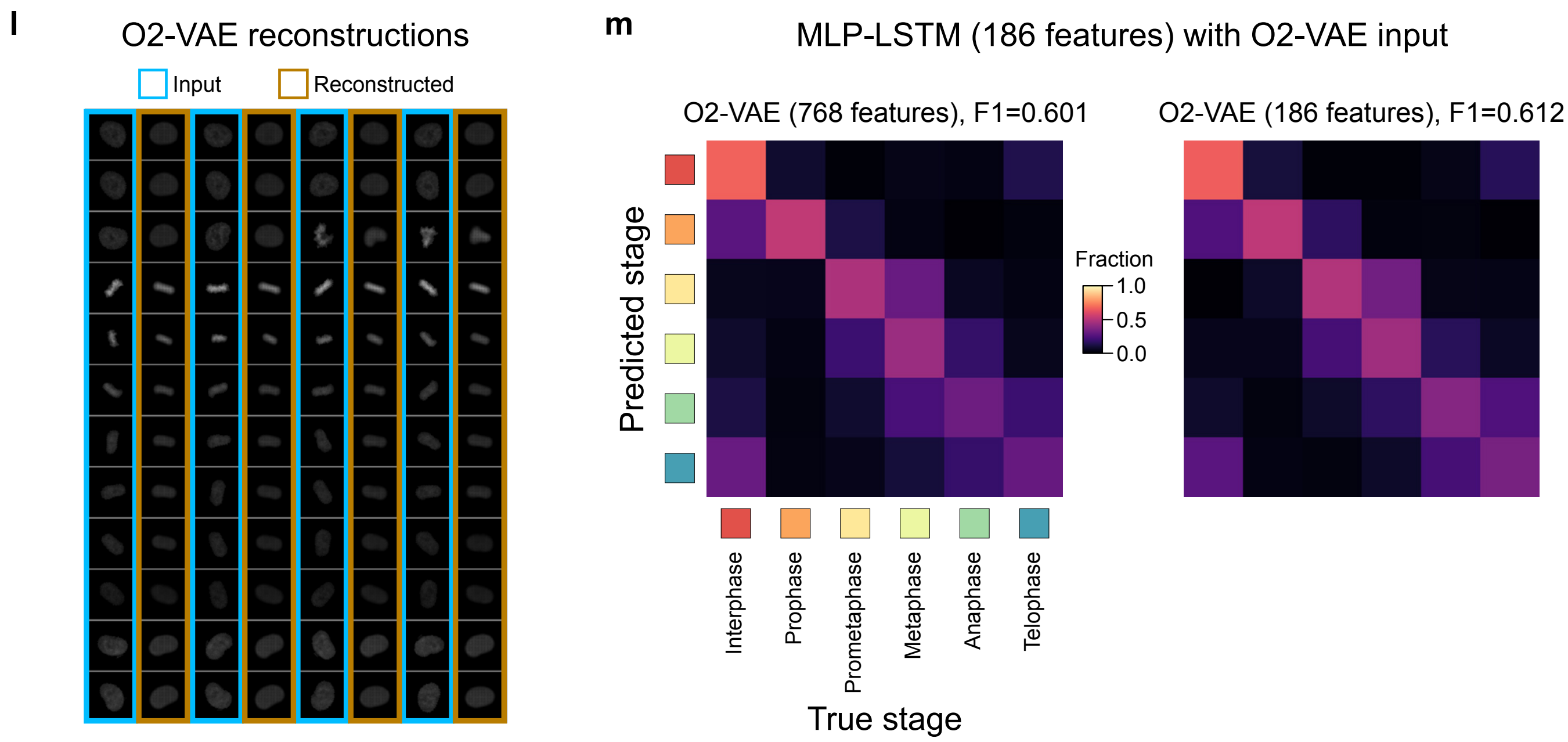

Supplementary Figure 7 cont. (l) Mitosis stage classification performance using self-supervised O2-VAE features. (m) Confusion matrix of classification using a MLP-LSTM autoencoder model with 186 features matching the feature number in the filtered SPOT-SAM phenomes, trained on either 768 or 186 features generated by a trained O2-VAE model (left to right).

SUPPLEMENTARY TABLES

Supplementary Table 1. Information on datasets used per figure.

Figure	Dataset	Statistics
Figure 1	n/a	n/a
Figure 2	n/a	n/a
Figure 3a,b	MPEG-7	n=1400 shapes, m=70 categories,<br>each category has 20 shapes
Figure 3c	Normalized Brodatz Textures	n=112 images
Figure 3d	Augmented Normalized Brodatz Textures (this work)	n = 112 images<br>m = 22,400 instances<br>(non-overlap crops of 640x640 image to 64x64, plus their 90^0 rotated version)
Figure 3e	MPEG-7	n=1400 shapes, m=70 categories,<br>each category has 20 shapes
Figure 3f,g	Brodatz textured MPEG-7 dataset (this work)	n = 5 shapes,<br>m = 5 textures,<br>l = 10,000 instances (5 shape classes x 20 images per shape class x 5 texture classes x 20 per texture class)
Figure 4	A2D YouTube	n = 20,026 frames, m = 3782 videos<br>capturing 7 object and 9 movement types
Figure 5a	2D Single Cell Tracking Challenge Dataset (U373 cells, train-split)	n = 1 video
Figure 5b-g	2D Single Cell Tracking Challenge Dataset (U373 cells, train-split)	k = 2 videos,<br>n = 1189 instances,<br>m = 29 tracks/cells
Figure 6	LIVECell dataset (test-split)	n = 451,518 cells:<br><br>A172: 32,935<br>BT-474: 32,565<br>BV2: 89,821<br>Huh7: 11,502<br>SH-SY5Y: 75,886<br>MCF7: 96,882<br>SKBr3: 64,860<br>SKOV3: 47,067
Figure 7	Hela H2B mitosis dataset	k = 7 videos<br>n = 12,714 nuclei instances<br><br>interphase: 7871<br>prophase: 460<br>prometaphase: 698<br>metaphase: 1645<br>anaphase: 714<br>telophase: 1326

		m = 326 nuclei/tracks
Supplementary Figure 1a	MPEG-7	n=1 image
Supplementary Figure 1b	MPEG-7	mean of 1400 images per n contour point setting
Supplementary Figure 1c	MNIST	n=1 image per digit,<br>each digit is augmented to be 42 images
Supplementary Figure 1d,e	MNIST	n=3 digits, m=42 images per digit
Supplementary Figure 1f-h	MNIST	n=10 digits, m=42 images per digit
Supplementary Figure 1i	MNIST	n=3 digits, m=2 rotations (+15, +45 degree rotations)
Supplementary Figure 1j-m	MNIST	n=3 digits, m = 4 motion rotations (+/-15, +/-45), l = 12 rotations, i.e. each digit has 48 images
Supplementary Figure 1n,o	MNIST	n=10 digits, m = 4 motion rotations (+/-15, +/-45), l = 12 rotations, i.e. each digit has 48 images
Supplementary Figure 2	n/a	n/a
Supplementary Figure 3a,b	MPEG-7	n=1400 shapes, m=70 categories,<br>each category has 20 shapes
Supplementary Figure 3c,d	Augmented Normalized Brodatz Textures (this work)	n = 112 images<br>m = 22,400 instances<br>(non-overlap crops of 640x640 image to 64x64, plus their 90^0 rotated version)
Supplementary Figure 3e-h	Brodatz textured MPEG-7 dataset (this work)	n = 5 shapes,<br>m = 5 textures,<br>l = 10,000 instances (5 shape classes x 20 images per shape class x 5 texture classes x 20 per texture class)
Supplementary Figure 3i	MPEG-7	n=1400 shapes, m=70 categories,<br>each category has 20 shapes
Supplementary Figure 3j	Augmented Normalized Brodatz Textures (this work)	n = 112 images<br>m = 22,400 instances<br>(non-overlap crops of 640x640 image to 64x64, plus their 90^0 rotated version)
Supplementary Figure 3k-x	Brodatz textured MPEG-7 dataset (this work)	n = 5 shapes,<br>m = 5 textures,<br>l = 10,000 instances (5 shape classes x 20 images per shape class x 5 texture classes x 20 per texture class)
Suppl. Fig 4a	A2D YouTube	n=43 unique actor-movement type pairings
Supplementary Figure 4b	A2D YouTube	n = 20,026 frames, m = 3782 videos

		capturing 7 object and 9 movement types
Supplementary Figure 5a	2D Single Cell Tracking Challenge Dataset (HL60 synthetic cells, train-split)	n = 1 video
Supplementary Figure 5b-g	2D Single Cell Tracking Challenge Dataset (HL60 synthetic cells, train-split)	k = 2 videos,<br>n = 5045 instances,<br>m = 185 tracks/cells
Supplementary Figure 6a	LIVECell dataset (test-split)	n = 1 representative image per cell line
Supplementary Figure 6b,d-j,l	LIVECell dataset (test-split)	n = 451,518 cells:<br><br>A172: 32,935<br>BT-474: 32,565<br>BV2: 89,821<br>Huh7: 11,502<br>SH-SY5Y: 75,886<br>MCF7: 96,882<br>SKBr3: 64,860<br>SKOV3: 47,067
Supplementary Figure 6k	LIVECell dataset (train-split)	n = 48 cells<br><br>Generally, in train/val split:<br>1,011,452 cells<br><br>A172: 83,023<br>BT-474: 82,815<br>BV2: 240,218<br>Huh7: 20,557<br>SH-SY5Y: 165,105<br>MCF7: 212,485<br>SKBr3: 168,336<br>SKOV3: 38,913<br><br>test split:<br>451,518 cells<br><br>A172: 32,935<br>BT-474: 32,565<br>BV2: 89,821<br>Huh7: 11,502<br>SH-SY5Y: 75,886<br>MCF7: 96,882<br>SKBr3: 64,860<br>SKOV3: 47,067
Supplementary Figure 7a	Hela H2B mitosis dataset	n = 1 nuclei (1 video sequence)
Supplementary Figure 7c-k,m	Hela H2B mitosis dataset	k = 7 videos (all)<br>n = 12,714 nuclei instances<br><br>interphase: 7871<br>prophase: 460<br>prometaphase: 698<br>metaphase: 1645<br>anaphase: 714<br>telophase: 1326

		m = 326 nuclei/tracks
Supplementary Figure 7l	Hela H2B mitosis dataset	n = 48 nuclei instances<br><br>Generally, in train split:<br>4 videos (n=7098 instances):<br><br>interphase: 4355<br>prophase: 250<br>prometaphase: 415<br>metaphase: 927<br>anaphase: 417<br>telophase: 734<br><br>In test split:<br>3 videos (n=5616 instances):<br><br>interphase: 3516<br>prophase: 210<br>prometaphase: 283<br>metaphase: 718<br>anaphase: 297<br>telophase: 592
