# Supplementary material for: Development of a universal imaging “phenome” using shape, appearance and motion (SAM) features and the SAM Phenotype Observation Tool (SPOT)
Source: Nat Commun. 2026 Aug 17;17:8409. doi: 10.1038/s41467-026-75505-8 (PMC13483437; doi:10.1038/s41467-026-75505-8)
Supplement: Supplementary file 2 [file 41467_2026_75505_MOESM2_ESM.pdf]

nature portfolio

Corresponding author(s): Felix Zhou
Xin Lu

Last updated by author(s): 11/06/2026

Reporting Summary

Nature Portfolio wishes to improve the reproducibility of the work that we publish. This form provides structure for consistency and transparency in reporting. For further information on Nature Portfolio policies, see our Editorial Policies and the Editorial Policy Checklist.

Statistics

For all statistical analyses, confirm that the following items are present in the figure legend, table legend, main text, or Methods section.

n/a	Confirmed	
☐	☒	The exact sample size (*n*) for each experimental group/condition, given as a discrete number and unit of measurement
☐	☒	A statement on whether measurements were taken from distinct samples or whether the same sample was measured repeatedly
☒	☐	The statistical test(s) used AND whether they are one- or two-sided<br>*Only common tests should be described solely by name; describe more complex techniques in the Methods section.*
☒	☐	A description of all covariates tested
☒	☐	A description of any assumptions or corrections, such as tests of normality and adjustment for multiple comparisons
☐	☒	A full description of the statistical parameters including central tendency (e.g. means) or other basic estimates (e.g. regression coefficient) AND variation (e.g. standard deviation) or associated estimates of uncertainty (e.g. confidence intervals)
☒	☐	For null hypothesis testing, the test statistic (e.g. *F*, *t*, *r*) with confidence intervals, effect sizes, degrees of freedom and *P* value noted<br>*Give P values as exact values whenever suitable.*
☒	☐	For Bayesian analysis, information on the choice of priors and Markov chain Monte Carlo settings
☒	☐	For hierarchical and complex designs, identification of the appropriate level for tests and full reporting of outcomes
☐	☒	Estimates of effect sizes (e.g. Cohen's *d*, Pearson's *r*), indicating how they were calculated

Our web collection on statistics for biologists contains articles on many of the points above.

Software and code

Policy information about availability of computer code

Data collection	All datasets used are publicly available and cited within the manuscript. The methods for processing the datasets are documented in Methods section of the paper.
Data analysis	All experiments were analyzed using our custom developed SPOT library, which is available free for academic use at https://github.com/fyz11/SPOT, installable from the Python Package Index (https://pypi.org/project/SAM-SPOT), and archived in Zenodo (https://zenodo.org/records/20629367). Example scripts and datasets are provided with the code for verification. CellProfiler features were computed using CellProfiler v4.2.6 downloaded from https://cellprofiler.org/releases. TisMORPH features were computed using code from https://github.com/Elaheh-Alizadeh/Quantifiction-of-shape-and-structure.git. Some image to video conversion was performed using FIJI, available at https://fiji.sc/. Extended focus projection uses the method of the ImageJ stack focusser plugin (https://imagej.net/ij/plugins/stack-focuser.html) either in Fiji or our Python version implementation in the SPOT library. Neural network models for feature extraction and classifiers were trained using Keras 3.3.3 with Tensorflow 2.16.1 backend with Python. DINOv3 training was performed using the official implementation available at https://github.com/facebookresearch/dinov3.git, and O2-VAE models were trained using the official code from https://github.com/jmhb0/o2vae.git.

For manuscripts utilizing custom algorithms or software that are central to the research but not yet described in published literature, software must be made available to editors and reviewers. We strongly encourage code deposition in a community repository (e.g. GitHub). See the Nature Portfolio guidelines for submitting code & software for further information.

Data

Policy information about availability of data

All manuscripts must include a data availability statement. This statement should provide the following information, where applicable:

- Accession codes, unique identifiers, or web links for publicly available datasets
- A description of any restrictions on data availability
- For clinical datasets or third party data, please ensure that the statement adheres to our policy

The developed general SPOT Python library used to compute and analyze SAM phenomes is available at GitHub, https://github.com/fyz11/SPOT, installable from the Python Package Index (https://pypi.org/project/SAM-SPOT), and archived in Zenodo (https://zenodo.org/records/20629367) for free academic use with example scripts and example data.

Research involving human participants, their data, or biological material

Policy information about studies with human participants or human data. See also policy information about sex, gender (identity/presentation), and sexual orientation and race, ethnicity and racism.

Reporting on sex and gender	N/A
Reporting on race, ethnicity, or other socially relevant groupings	N/A
Population characteristics	NA
Recruitment	NA
Ethics oversight	NA

Note that full information on the approval of the study protocol must also be provided in the manuscript.

Field-specific reporting

Please select the one below that is the best fit for your research. If you are not sure, read the appropriate sections before making your selection.

☒ Life sciences ☐ Behavioural & social sciences ☐ Ecological, evolutionary & environmental sciences

For a reference copy of the document with all sections, see nature.com/documents/nr-reporting-summary-flat.pdf

Life sciences study design

All studies must disclose on these points even when the disclosure is negative.

Sample size	We used all available data without exclusion with the designated train/val/test splits when available. For Hela H2B mitosis dataset where the splits were not provided we created an approximate 50-50 train/test split between the 7 videos by randomization.
Data exclusions	No data was excluded. Statistical outliers were detected and filtered out during analysis as part of out SPOT workflow.
Replication	We validated the replicability and general applicability of SPOT on publicly available datasets used for benchmarking. For computer vision: MPEG7 shape dataset, Brodatz textures, A2D dataset and MNIST dataset. For cell imaging: cell tracking challenge datasets, LIVECell and a Hela cell histone H2B dataset part of the CellCognition project.
Randomization	Randomization was used to create an approximate 50-50 train/test split between the 7 videos of the Hela H2B mitosis dataset for testing classifier training. Otherwise, we applied SPOT to all the available data as indicated.
Blinding	Blinding was not applicable in this study. We applied SPOT to all the available data

Reporting for specific materials, systems and methods

We require information from authors about some types of materials, experimental systems and methods used in many studies. Here, indicate whether each material, system or method listed is relevant to your study. If you are not sure if a list item applies to your research, read the appropriate section before selecting a response.

Materials & experimental systems

n/a	Involved in the study
☒	☐ Antibodies
☒	☐ Eukaryotic cell lines
☒	☐ Palaeontology and archaeology
☒	☐ Animals and other organisms
☒	☐ Clinical data
☒	☐ Dual use research of concern
☒	☐ Plants

Methods

n/a	Involved in the study
☒	☐ ChIP-seq
☒	☐ Flow cytometry
☒	☐ MRI-based neuroimaging

Plants

Seed stocks

Report on the source of all seed stocks or other plant material used. If applicable, state the seed stock centre and catalogue number. If plant specimens were collected from the field, describe the collection location, date and sampling procedures.

Novel plant genotypes

Describe the methods by which all novel plant genotypes were produced. This includes those generated by transgenic approaches, gene editing, chemical/radiation-based mutagenesis and hybridization. For transgenic lines, describe the transformation method, the number of independent lines analyzed and the generation upon which experiments were performed. For gene-edited lines, describe the editor used, the endogenous sequence targeted for editing, the targeting guide RNA sequence (if applicable) and how the editor was applied.

Authentication

Describe any authentication procedures for each seed stock used or novel genotype generated. Describe any experiments used to assess the effect of a mutation and, where applicable, how potential secondary effects (e.g. second site T-DNA insertions, mosiacism, off-target gene editing) were examined.
