# Supplementary material for: Development of a universal imaging “phenome” using shape, appearance and motion (SAM) features and the SAM Phenotype Observation Tool (SPOT)
Source: Nat Commun. 2026 Aug 17;17:8409. doi: 10.1038/s41467-026-75505-8 (PMC13483437; doi:10.1038/s41467-026-75505-8)
Supplement: Supplementary file 3 [file 41467_2026_75505_MOESM3_ESM.pdf]

Supplementary Movie 1. Reference cell segmentation and tracking of migrating glioblastoma-astrocytoma U373 and dividing HL60 leukaemia cells from the 2D cell tracking challenge. Videos show the curated reference individual cell segmentations and tracks for both videos in the training data of U373 and HL60 cells used for SPOT analysis. Individually tracked cells are colored uniquely. Where cells move partially out of the field of view, these individual instances are removed prior to analysis and are not labeled. Consequently, if a cell leaves the field of view and later returns, the separate tracks are treated as separate entities for Hidden Markov Model (HMM) analysis. Movie frames are acquired every 15 min for U373 and every 29 min for HL60. Scalebar: 50 μm for U373, 10 μm for HL60.

Supplementary Data 1. SAM features

Supplementary Data 2. SAM features computer vision validation plus supervised

Supplementary Data 3. MLP cell line classifier using SAM or DL features: LIVECell

Supplementary Data 4. SVM mitosis classifier using SAM or DL features: HeLa
