# Supplementary material for: Development of a universal imaging “phenome” using shape, appearance and motion (SAM) features and the SAM Phenotype Observation Tool (SPOT)
Source: Nat Commun. 2026 Aug 17;17:8409. doi: 10.1038/s41467-026-75505-8 (PMC13483437; doi:10.1038/s41467-026-75505-8)
Supplement: Supplementary file 4 [file 41467_2026_75505_MOESM9_ESM.pdf]

nature portfolio

Peer Review File

Development of a universal imaging “phenome” using Shape, Appearance and Motion (SAM) features and the SAM Observation Tool (SPOT)

Corresponding Author: Professor Xin Lu

This file contains all reviewer reports in order by version, followed by all author rebuttals in order by version.

Version 1:

Reviewer comments:

Reviewer #1

(Remarks to the Author)
I don't agree with the split of the original paper to two.

(Remarks on code availability)
I cannot run it successfully

Reviewer #2

(Remarks to the Author)
I would like to congratulate the authors on the substantial improvements made to their manuscript. Splitting the original version into two separate manuscripts has significantly improved the clarity and readability of the work, making the core concepts and contributions more accessible to the reader.

The addition of more biologically relevant datasets, particularly the inclusion of the LIVECell database, greatly enhances the manuscript’s applicability to real-world cell-based imaging studies. I found the inclusion of machine learning classifiers to predict cell lines and mitotic states particularly compelling. These results convincingly demonstrate that SAM-SPOT extracts phenotypically meaningful and discriminative features that can be used for accurate classification.

I’m particularly curious about how SAM-SPOT would perform in the context of cells exposed to a diverse panel of compounds with known, distinct, or overlapping mechanisms of action. It would be interesting to explore whether SAM-SPOT can effectively stratify such compounds based on the dynamic phenotypic responses it captures. But that does not need to be part if the current manuscript.

(Remarks on code availability)

Reviewer #4

(Remarks to the Author)
1. What are the noteworthy results?

The authors claim that a universal set of 2185 image-derived features is developed to quantify an object’s phenotype (“standard” image-based “phenome”); subsequently, they provide a tool for image “sequencing” analysis of phenomes. For temporal analysis, they compute the HMM phenotype cluster transition graph that shows the different state of each detected phenome cluster over time.

2. How does it compare to the established literature?

I. In the introduction, the authors mention two previous feature descriptors, CellProfiler and TISmorph. But they only compare

to Cellprofiler. The comparison is not enough. The authors should compare to TISmorph36 too.

II. The comparison is limited to two methods, i.e., merely done for CNN and CNN-LSTM based autoencoder. The authors should compare to more methods e.g. generative models and graph-based models. Two examples of such methods: A) Dynamic graph convolutional foldingnet in "3D single-cell shape analysis of cancer cells using geometric deep learning", B) "DeepSea is an efficient deep-learning model for single-cell segmentation and tracking in time-lapse microscopy". Then given their claim of rotation-invariance, they should compare to different VAE tackling rotation-invariance and not just a naive auto-encoder: C) “ME-VAE: Multi-Encoder Variational AutoEncoder for Controlling Multiple Transformational Features in Single Cell Image Analysis”

3. Does the work support the conclusions and claims, or is additional evidence needed?

I. In suppl. Fig. 8 the number of features differ between SAM and CNN-LSTM. What happens if the number of features set to be the same? That way it may be more fair to compare. The same should be done for the table of results comparing the accuracy of CNN and CNN-LSTM (right now SAM has not the same number of features as the other two).

II. In all illustrations of HMM cluster transition probability, it looks like a state machine rather than a probabilistic state machine (HMM. The author should annotate what each edge coloring represents as a probability.

III. See points 2 and 5 and address them too.

4. Are there any flaws in the data analysis, interpretation and conclusions? Do these prohibit publication or require revision?

I cannot see any.

5. Is the methodology sound? Does the work meet the expected standards in your field?

I. I do not understand their HMM. I thought first Kmeans is used to find 7-8 clusters and then for "each cluster" the temporal analysis is done (this is what the authors claim in line 1319 section). This makes sense to me. However, in their HMM transition (shape of state machine with color-coded arrows), I see that they show transition between those 7-8 clusters. Why this? and why not the state transition for each cluster separately? If the author answer this as "we want to see transition between different clusters" then my judgment is that their modeling is not sensible - they should NOT first do Kmeans to detect states of an HMM (the whole point of HMM is to find those states). Instead, they should have directly applied HMM and then Viterbi to find the most probable sequence, which would unfold the trajectory of states - the states would be detected by fitting the HMM and would not need to have K-means in advance.

II. The authors should provide answer to this: why SAM? I am not sure if motion is sufficiently captured since it is "Motion as combined changes in the object’s shape, appearance, and centroid between consecutive timepoints". That is, why not separately consider rotation and other potential spacial variations which can change the perception. See, for example, “ME-VAE: Multi-Encoder Variational AutoEncoder for Controlling Multiple Transformational Features in Single Cell Image Analysis”. Finally, a way to convince reviewers is to show an example of "rotation" and an example of "polar orientation" that is correctly detected using their method, and it is not confused and perceived as a change in shape.

III. The authors state that "Motion as combined changes in the object’s shape, appearance, and centroid between consecutive timepoints". Mathematically, thinking SAM should capture mutually exclusive features of change, but due to their definition of motion, it implies that motion does not capture a distinct feature. How do the authors support the sufficiency of their definition of the motion?

IV. How is it determined that rotation should always be ignored? Maybe a rotation is part of a meaningful motion? I am not sure about this but I put this as a question to be answered.

V. The preprocessing contains removal of highly variant features "All dataset dependent preprocessing steps such as zero-valued and high variance features". I do not see why this is sensible. Conversely, I can see that high variance features are useful signals.

6. Is there enough detail provided in the methods for the work to be reproduced?

Yes.

(Remarks on code availability)
1. What are the noteworthy results?

1) The authors claim that a universal set of 2185 image-derived features is developed to quantify an object’s phenotype (“standard” image-based “phenome”); 2) they provide a tool for image “sequencing” analysis of phenomes. For temporal analysis, they compute the HMM phenotype cluster transition graph that shows the different state of each detected phenome cluster over time.

2. How does it compare to the established literature?

I. In the introduction, the authors mention two previous feature descriptors, CellProfiler and TISmorph. But they only compare to Cellprofiler. The comparison is not enough. The authors should compare to TISmorph36 too.

II. The comparison is limited to two methods, i.e., merely done for CNN and CNN-LSTM based autoencoder. The authors should compare to more methods e.g. generative models and graph-based models. Two examples of such methods: A) Dynamic graph convolutional foldingnet in "3D single-cell shape analysis of cancer cells using geometric deep learning", B) "DeepSea is an efficient deep-learning model for single-cell segmentation and tracking in time-lapse microscopy". Then given their claim of rotation-invariance, they should compare to different VAE tackling rotation-invariance and not just a naive auto-encoder: C) “ME-VAE: Multi-Encoder Variational AutoEncoder for Controlling Multiple Transformational Features in Single Cell Image Analysis”

3. Does the work support the conclusions and claims, or is additional evidence needed?

I. In suppl. Fig. 8 the number of features differ between SAM and CNN-LSTM. What happens if the number of features set to be the same? That way it may be more fair to compare. The same should be done for the table of results comparing the accuracy of CNN and CNN-LSTM (right now SAM has not the same number of features as the other two).

II. In all illustrations of HMM cluster transition probability, it looks like a state machine rather than a probabilistic state machine (HMM. The author should annotate what each edge coloring represents as a probability.

III. See points 2 and 5 and address them too.

4. Are there any flaws in the data analysis, interpretation and conclusions? Do these prohibit publication or require revision?

I cannot see any.

5. Is the methodology sound? Does the work meet the expected standards in your field?

I. I do not understand their HMM. I thought first Kmeans is used to find 7-8 clusters and then for "each cluster" the temporal analysis is done (this is what the authors claim in line 1319 section). This makes sense to me. However, in their HMM transition (shape of state machine with color-coded arrows), I see that they show transition between those 7-8 clusters. Why this? and why not the state transition for each cluster separately? If the author answer this as "we want to see transition between different clusters" then my judgment is that their modeling is not sensible - they should NOT first do Kmeans to detect states of an HMM (the whole point of HMM is to find those states). Instead, they should have directly applied HMM and then Viterbi to find the most probable sequence, which would unfold the trajectory of states - the states would be detected by fitting the HMM and would not need to have K-means in advance.

II. The authors should provide answer to this: why SAM? I am not sure if motion is sufficiently captured since it is "Motion as combined changes in the object’s shape, appearance, and centroid between consecutive timepoints". That is, why not separately consider rotation and other potential spacial variations which can change the perception. See, for example, “ME-VAE: Multi-Encoder Variational AutoEncoder for Controlling Multiple Transformational Features in Single Cell Image Analysis”. Finally, a way to convince reviewers is to show an example of "rotation" and an example of "polar orientation" that is correctly detected using their method, and it is not confused and perceived as a change in shape.

III. The authors state that "Motion as combined changes in the object’s shape, appearance, and centroid between consecutive timepoints". Mathematically, thinking SAM should capture mutually exclusive features of change, but due to their definition of motion, it implies that motion does not capture a distinct feature. How do the authors support the sufficiency of their definition of the motion?

IV. How is it determined that rotation should always be ignored? Maybe a rotation is part of a meaningful motion? I am not sure about this but I put this as a question to be answered.

V. The preprocessing contains removal of highly variant features "All dataset dependent preprocessing steps such as zero-valued and high variance features". I do not see why this is sensible. Conversely, I can see that high variance features are useful signals.

6. Is there enough detail provided in the methods for the work to be reproduced?

Yes.

Version 2:

Reviewer comments:

Reviewer #4

(Remarks to the Author)
All my questions are answered and addressed. Thank you for all the effort.

(Remarks on code availability)

Open Access This Peer Review File is licensed under a Creative Commons Attribution 4.0 International License, which permits use, sharing, adaptation, distribution and reproduction in any medium or format, as long as you give appropriate credit to the original author(s) and the source, provide a link to the Creative Commons license, and indicate if changes were made.
In cases where reviewers are anonymous, credit should be given to 'Anonymous Referee' and the source.
The images or other third party material in this Peer Review File are included in the article's Creative Commons license, unless indicated otherwise in a credit line to the material. If material is not included in the article's Creative Commons license and your intended use is not permitted by statutory regulation or exceeds the permitted use, you will need to obtain permission directly from the copyright holder.
To view a copy of this license, visit https://creativecommons.org/licenses/by/4.0/

Reply to Reviewers' main concerns:

The present manuscript (NCOMMS-25-25126) is a new submission, born from the initial review of our resubmitted, revised organoid-focussed manuscript NCOMMS-23-61673-C. Specifically it focusses on the development and benchmarking of the analytical method underpinning the original submission on public datasets from computer vision and cell imaging. A substantial portion of the work in this manuscript has therefore been reviewed by three reviewers.

In this first review, the Reviewers raised three main concerns:

1) SPOT's and SAM phenome's ability to deal with the presence of artefacts in analysis;

2) the impact of potential inaccuracies in object segmentation and tracking; and

3) SPOT's suitability for 3D time-series imaging data.

To address all three of these concerns systematically and objectively, we decided to divide the initial submission into two independent yet complementary companion manuscripts (explained in detail below). Our replies to reviewers' main comments relevant to this methods-focussed work (namely points 1 and 2) are summarised below and a point-by point reply is also attached for reference.

1. Potential sensitivity to artefacts:

Reviewer 2: My main concern is the images that are used for the organoids. SAM quantifies 2185 features, so it is very sensitive to imaging artifacts.
From image 3B it is very evident that the clusters are mainly based on imaging artefacts. For example, cluster 3 are out of focus organoids, cluster 2 are overexposed organoids, cluster 7 are underexposed organoid, cluster 4 out of focus and underexposed... Although there might be some phenotypic differences, features extracted from these images are completely unreliable due to the imaging artefacts.

Reply:
We apologise for not making it clear that including and reconciling artefacts within SPOT's analysis is a unique designed feature. The phenotype clustering is designed to identify different 'modes' or SAM imaging 'states' in the dataset by discretely partitioning the UMAP phenomic landscape. Each cluster is not necessarily a biological phenotype, and more than one cluster could relate to the same biological process e.g., two clusters relate to cell death. The primary biological analysis is in the phenotype evolution of organoids between conditions. SPOT assumes that over time, only phenotype clusters corresponding to true biological phenotype dynamics will exhibit differential temporal evolution, and this gives a signature to cluster conditions. Not all analysis uses this clustering. SAM phenotype trajectories are derived by the majority UMAP density in each temporal bin. In all cases, artefacts, when present, are assumed to not exhibit temporal evolution and to affect all conditions equally. We demonstrate the interpretation of phenotype cluster in using the simulated organoid dataset (Fig. 2 of organoids-focussed

manuscript NCOMMS-23-61673-C, included below for reference) and new supplementary chemotherapy drug screens analysed with the addition of a fluorescent caspase marker (Suppl. Fig. 3a of organoids-focussed manuscript NCOMMS-23-61673-C, included in the response to Reviewer 2, point 5, below). In the latter, we found at least three SAM phenotype clusters associated with elevated caspase expression, indicative of different modes of death.

Imaging artefacts are almost always present in some manner during timelapse acquisition. Importantly, these artefacts differ between imaging modality and imaged subject. As a tool designed for high-content analysis, we believe that it would be a limitation to have to firstly carefully remove artefacts, which requires domain expertise and potentially extensive manual proofreading. Naturally, if the user has trained an appropriate artefact detector for their data, this can be applied to further improve analysis and enhance biological interpretation. Nevertheless, as we demonstrate by analysing simulated organoids subject to defined artefact type perturbations in Suppl. Fig. 2 of our companion manuscript NCOMMS-23-61673-C, SPOT is either robust to an artefact type (such as blurring and intensity changes due to photobleaching), or, it makes the artefacts easily identifiable in the 2D UMAP to allow data subsetting (see below for details of simulated organoid datasets A and B, and Suppl. Fig. 2a, 2b, 2c). For example, when we perturbed the ground truth simulated dataset with 'union' segmentation errors of varying severities (illustrated in companion manuscript NCOMMS-23-61673-C's Suppl. Fig. 2b a, included below), the UMAP cleanly partitions the ground truth from the perturbed conditions. We can thus subset for and reperform analysis on only the unaffected images (companion manuscript NCOMMS-23-61673-C Suppl. Fig. 2b c, included below). We include extracts from the Methods section of our companion organoid-focussed manuscript which detail the simulated organoids datasets as an appendix at the end of this point-by-point rebuttal.

Simulated Dataset A

We generated a synthetic dataset of simulated 3D organoids, that evolve across time from a small, round initial state into a phenotypically distinct final state (7 conditions, 45 organoids per condition, and 30 frames per organoid). Each of the conditions share a common starting state with identical shape, appearance, motion behaviour parameterisation, before diverging over the duration of the 30-frame time course into specifically-parameterised, condition-specific end states. To generate this dataset, we define one condition-dependent end state as the 'base organoid', with 'basal' parameter values. Then, using the base organoid parameterisation as the template, we modified parameters influencing each of shape, appearance, and motion in isolation, to create 6 further extremal 'high' or 'low' conditions for each concept. The dataset is comprised of 3-dimensional organoid simulations and their 2-dimensional projections along with pixel-perfect segmentation masks. To generate the 2-dimensional projections, we used an equivalent method to the extended depth of focus projection used on our real timelapse z-stacks. We subjected the entirety of Simulated Dataset A to 8 different perturbation types, each across 9 severity levels, to identify effects on the analysis relative to the known ground truth.

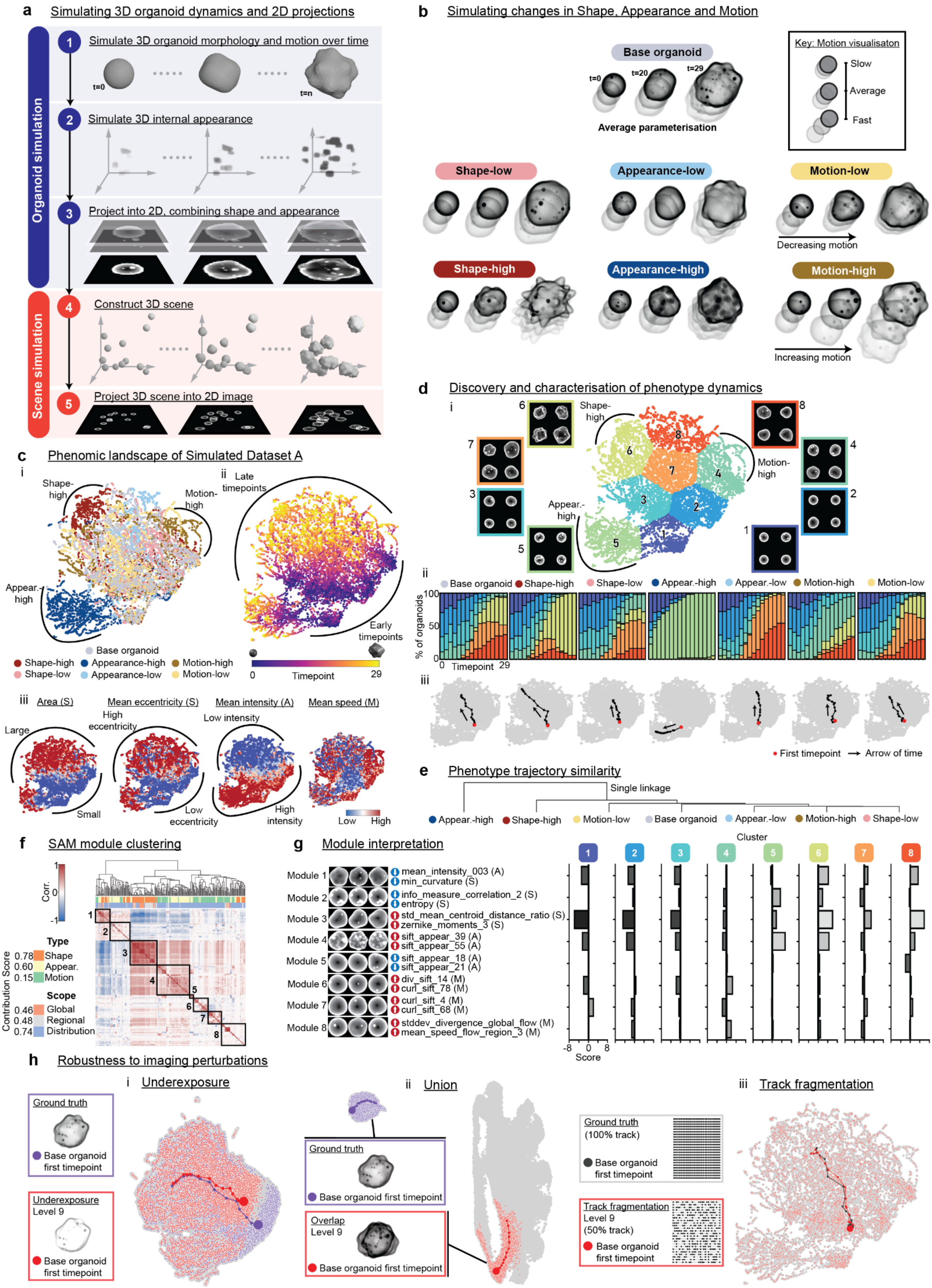
a Simulating 3D organoid dynamics and 2D projections
Organoid simulation
1 Simulate 3D organoid morphology and motion over time
t=0
t=n
2 Simulate 3D internal appearance
3 Project into 2D, combining shape and appearance
Scene simulation
4 Construct 3D scene
5 Project 3D scene into 2D image
b Simulating changes in Shape, Appearance and Motion
Base organoid
t=0
t=20
t=29
Average parameterisation
Key: Motion visualisaton
Slow
Average
Fast
Shape-low
Appearance-low
Motion-low
Decreasing motion
Shape-high
Appearance-high
Motion-high
Increasing motion
c Phenomic landscape of Simulated Dataset A
i
Shape-high
Motion-high
Appear.-high
Base organoid
Shape-high
Appearance-high
Motion-high
Shape-low
Appearance-low
Motion-low
ii Late timepoints
Early timepoints
0 Timepoint 29
iii Area (S)
Mean eccentricity (S)
Mean intensity (A)
Mean speed (M)
Large
Small
High eccentricity
Low eccentricity
Low intensity
High intensity
Low High
d Discovery and characterisation of phenotype dynamics
i
Shape-high
Motion-high
Appear.-high
ii
Base organoid
Shape-high
Shape-low
Appear.-high
Appear.-low
Motion-high
Motion-low
% of organoids
100
50
0
0 Timepoint 29
iii
First timepoint
Arrow of time
e Phenotype trajectory similarity
Single linkage
Appear.-high
Shape-high
Motion-low
Base organoid
Appear.-low
Motion-high
Shape-low
f SAM module clustering
Corr.
1
0
-1
Type
Shape
Appear.
Motion
Scope
Global
Regional
Distribution
Contribution Score
0.78
0.60
0.15
0.46
0.48
0.74
g Module interpretation
Module 1 mean_intensity_003 (A) min_curvature (S)
Module 2 info_measure_correlation_2 (S) entropy (S)
Module 3 std_mean_centroid_distance_ratio (S) zernike_moments_3 (S)
Module 4 sift_appear_39 (A) sift_appear_55 (A)
Module 5 sift_appear_18 (A) sift_appear_21 (A)
Module 6 div_sift_14 (M) curl_sift_78 (M)
Module 7 curl_sift_4 (M) curl_sift_68 (M)
Module 8 stddev_divergence_global_flow (M) mean_speed_flow_region_3 (M)
Cluster
-8 0 8
Score
h Robustness to imaging perturbations
i Underexposure
Ground truth
Base organoid first timepoint
Underexposure
Level 9
Base organoid first timepoint
ii Union
Ground truth
Base organoid first timepoint
Overlap
Level 9
Base organoid first timepoint
iii Track fragmentation
Ground truth
(100% track)
Base organoid first timepoint
Track fragmentation
Level 9
(50% track)
Base organoid first timepoint

Figure 2 of* NCOMMS-23-61673-C*. SPOT analysis of synthetic organoid video dataset with prescribed shape, appearance and motion dynamics.* *a) *Schematic depicting the main steps in the creation of the synthetic 3D organoid dataset. Individual organoid dynamics were generated by evolving 3D meshes and simulating internal appearance changes across time. For each timepoint, shape and appearance were then independently projected and combined into a single 2D image. 3D scenes are built, and associated 2D projections are generated.* ***b)*** *Schematic of the 7 organoid conditions comprising Simulated Dataset A. The first, intermediate and last timepoints are displayed, with motion represented by shadows.* ***c)*** *UMAP plots (n=9744) colored to display (i) ground truth phenotypes, (ii) timepoint of each synthetic organoid instance, (iii) size-standardized representative image patches for each phenotype cluster, and (iv) relative expression of select SAM metrics.* ***d)*** *Characterization of phenotype dynamics – UMAP phenomic landscape partitioned with phenotype clusters, colored dark blue to dark orange and numbered in ascending order of mean shape eccentricity. Clusters were identified by k-means clustering and the elbow method. For each cluster, four representative images arranged as a 2x2 grid are shown, using their real size for comparison of scale across clusters. (i) Stacked barplots show the temporal changes in phenotype cluster relative frequency across discrete time bins, per phenotype. (ii) Temporal phenotype trajectories show the progression in organoid SAM in each condition across the phenomic landscape across time. Red point indicates the origin of the trajectory at t=0.* ***e)*** *Single-linkage hierarchical clustering of phenotype trajectories.* ***f)*** *Automated hierarchical clustering of the covariation between SAM features to identify SAM feature modules (left, modules outlined and numbered along the diagonal).* ***g)*** *Mean expression of each SAM module for each cluster (as defined in 2d), with bars shaded by relative expression score (right). Each module is depicted with its top three most representative images, its top two driving SAM features and whether the feature is enriched (up arrow) or depleted (down arrow). Individual SAM features are described in depth in our companion methods-focussed manuscript (Reviewers are requested to contact the Editor for access).* ***h)*** *UMAP landscape of all datapoints (light grey) for the underexposure, union, and track fragmentation perturbation conditions with the ground truth (purple) and perturbation level 9 (red) emphasized. Trajectories for the ground truth and perturbation level 9 are plotted on top for the 'base organoid' condition only. Only perturbation level 9 is emphasized in the tracking error plot as the ground truth is all points.*

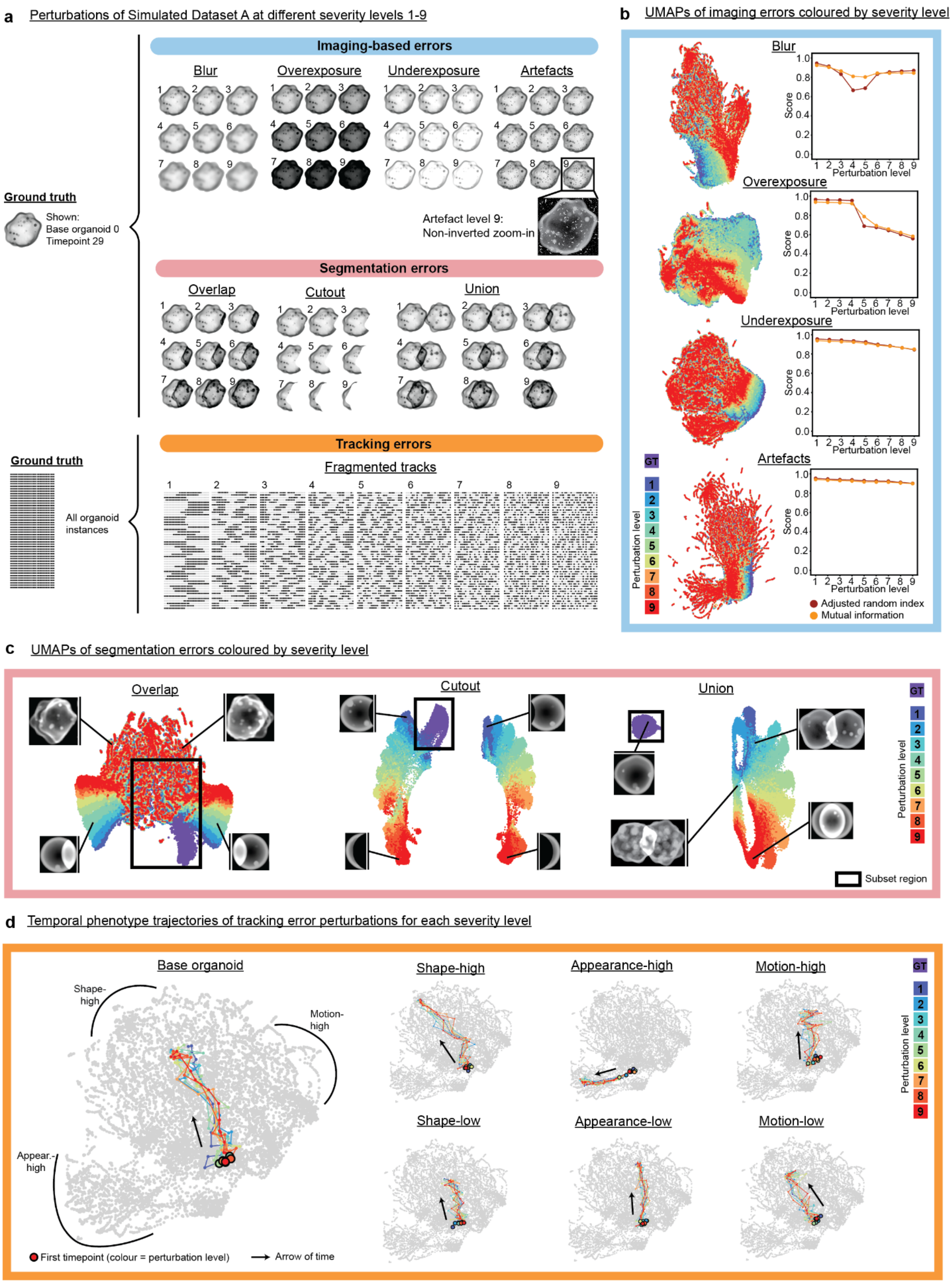

Supplementary Fig. 2b of NCOMMS-23-61673-C. Robustness of SPOT analysis to imaging artefact, segmentation and tracking errors. a) *Depiction of simulated perturbations grouped into imaging, segmentation and tracking error categories across 9 severity levels. Imaging and segmentation errors are illustrated using frame 29 of 'base organoid' 1 as a reference image. Fragmented tracks are represented by consecutive black squares in the horizontal direction. The ground truth dataset has 100% complete organoid tracks, 100% black squares. Each increasing severity level includes an additional track*

breaking but overall temporal coverage (total black squares) is fixed at 50%. Organoid exemplar images are depicted with inverted intensity such that underexposure appears bright and overexposure appears dark. A non-inverted artefact level 9 exemplar image is shown for clarity. ***b)*** *For each of blur, overexposure, underexposure and artefact, a single UMAP plot (n=97440) was generated to jointly analyze ground-truth and its perturbations, colored by perturbation level. After K-means clustering as in Fig.2c, adjusted random index and mutual information was computed to assess phenotype clustering agreement between the ground truth and each perturbation level.* ***c)*** *UMAP for each segmentation perturbation are colored by perturbation severity level, showing that the ground truth can easily be isolated (black crop boxes), thereby enabling exclusion of a both severe and subtle segmentation error manifestations. Reference images are depicted alongside each UMAP, with black lines locating each instance on the plot.* ***d)*** *Temporal phenotype trajectories of each condition, for ground-truth and all track fragmentation levels.*

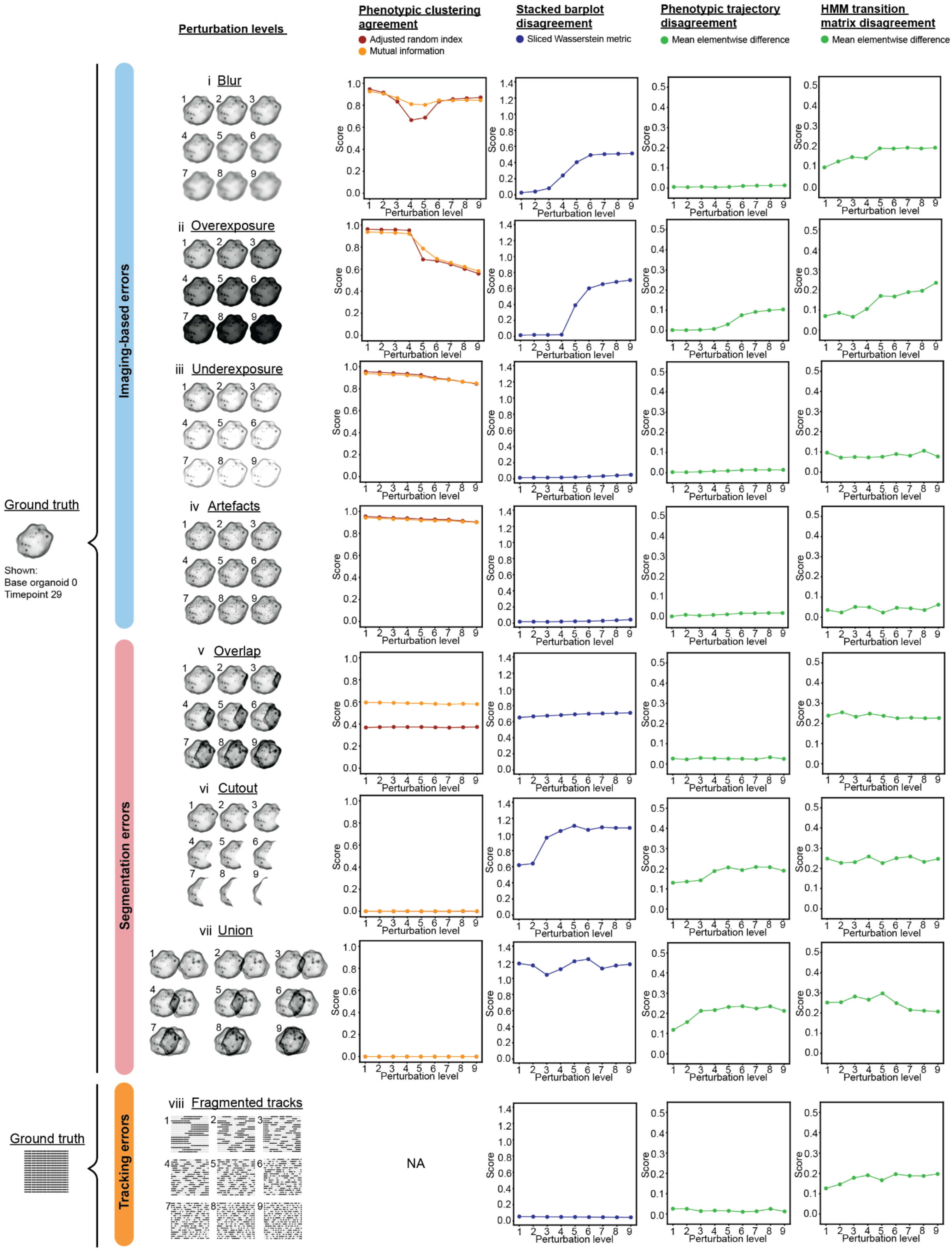
Perturbation levels
Phenotypic clustering agreement
Adjusted random index
Mutual information
Stacked barplot disagreement
Sliced Wasserstein metric
Phenotypic trajectory disagreement
Mean elementwise difference
HMM transition matrix disagreement
Mean elementwise difference
Ground truth
Shown:
Base organoid 0
Timepoint 29
Imaging-based errors
i Blur
ii Overexposure
iii Underexposure
iv Artefacts
Segmentation errors
v Overlap
vi Cutout
vii Union
Tracking errors
viii Fragmented tracks
Ground truth
NA
Score
Perturbation level

Supplementary Fig. 2c of NCOMMS-23-61673-C. ***Quantitative assessment of SPOT analysis robustness to imaging artefact, segmentation and tracking errors.*** *Schematic of increasing severity level of perturbation, and i) phenotype clustering agreement, ii) stacked barplot, iii) phenotype trajectory and iv) HMM cluster transition matrix disagreement metrics of ground-truth with each perturbation level (left to right). For agreement metrics, the higher the value, the greater the concordance with ground-truth. For disagreement metrics, the lower the value, the greater the concordance with ground-truth. Multiple independent evaluation measures were used to evaluate phenotype clustering agreement and stacked barplot disagreement. Mean elementwise difference was used to compute phenotype trajectory and HMM cluster transition matrix disagreements, (Methods).*

2. Potential inaccuracies in object segmentation and tracking

Reviewer 1: The success of the whole pipeline is dependent on accurate object tracking/tracklet. Can you quantify the accuracy of this step of your pipeline and discuss how this accuracy will affect the downstream results numerically?

Reviewer 2: The label-free organoid segmentation is not accurate enough, and tracking of the organoids is not consistent over time (e.g. video 4) or the density of the organoids is way too high (e.g. video 5). Organoids merge together, overlap, get detected – undetected,… so there is no consistency that allows for kinetic measurements crucial for SAM. Again, this can all be resolved by the use of proper imaging-compatible culturing techniques and plates.

Reply:
SPOT inherently includes safeguards that may affect analysis. We use the state-of-the-art tracking paradigm of 'detect then link' whereby organoids are detected independently in each frame, then detected organoids are linked across successive frames by bipartite matching. We demonstrate this in our companion, organoids-focussed manuscript: with organoid detection, SPOT enforces a minimum temporal consistency in segmentation; all instances of a tracked organoid must have a frame-to-frame shape overlap (as measured by intersection-over-union (IoU)) greater than a user specified threshold. If this cut-off is too high, matching may be too stringent to shape changes between frames. We use a cut-off of IoU=0.25. We also emphasise that the tracking is implemented only to identify genuine organoids (alive or dead) which we define as being tracked for a minimum number of frames (here, 5 frames) from potential short-lived transient artefacts e.g., floating debris. In SPOT analysis, the tracked organoids at different timepoints are mapped as independent individual points in the UMAP. Only the motion features require tracking to compute. Even so, it depends only on a consecutive pair of accurate segmentations. In summary, SPOT is expected to be robust even if not all object instances are perfectly segmented (due to overlap for example), and robust in the case where some object instances are undetected or filtered out (such as by the IOU tracker, causing fragmented tracks). We validate our claims using a dataset of simulated 3D organoids, ("Simulated Dataset A", see details in response just above) by introducing varying degrees of segmentation errors 'cutout' and 'union'), that could result from overlap (see figure in response just above, named Suppl. Fig. 2b in our companion

organoid-focussed manuscript NCOMMS-23-61673-C). If such instances occur and are highly prevalent, we find they cluster distinctly and can be removed (Suppl. Fig. 2b c of companion manuscript NCOMMS-23-61673-C, included in response just above). In addition, we simulated the effect of various degrees of track fragmentation (such as that caused by organoid instances remaining undetected or being filtered out), showing that the conclusions drawn from SPOT analysis remain unaffected (Suppl. Fig. 2b d of companion manuscript NCOMMS-23-61673-C, included in response just above).

Reviewer #1 (Remarks to the Author):

This manuscript introduced a pipeline to track the phenotypic changes of objects in 2D videos, and applied it on living-cell videos. Together with single cell sequencing data, this tool may study the phenotype-genotype association. I have the following comments:

1) It is hard to call any chosen summary features as comprehensive and unbiased phenotypic description of the video, as long as you do not use all the pixel information.

We apologise for not defining the terms “comprehensive” and “unbiased” clearly in the text. We use the terms “comprehensive” and “unbiased” to refer to how the image features are acquired and how the acquired data are analysed, respectively. Specifically, SAM features are “comprehensive” because they have been designed to be a generally applicable set of image features covering all spatial scales of global, local, and regional (akin to late and early layers of a neural network) and the different attributes of shape, appearance and motion. We note local distribution features are derived by using all the pixel information. The specific composition of features is now illustrated in Fig. 2 of the present manuscript NCOMMS-25-25126.

We use the term *unbiased* to emphasise that all features in the SAM phenome can be and are computed for all datasets in the same manner and are not ‘handcrafted’ to target a particular phenotype *a priori* (e.g. organoid branching-specific) nor require adaptation towards any imaging modality or imaged object (e.g. number of branches is a feature specific only to objects that branch). This is in contrast to data-driven learning such as convolutional neural network (CNN) features trained on a particular dataset, which is a “*biased”* approach in that it necessarily requires fine-tuning on the target dataset to guarantee relevance of generated features. In the present methods-focussed manuscript, we describe in detail the rationale used for constructing the SAM phenome (Present submission NCOMMS-25-25126: Results, Line 178 and Fig. 1, Suppl. Table 1).

We agree with this reviewer that a higher number of features may be more comprehensive, but is offset by the curse of dimensionality, necessitating more sophisticated preprocessing. In the present methods-focussed manuscript, we have now provided expanded and detailed description and explanation as to why 2185 SAM features is sufficiently comprehensive but minimally complex to constitute a SAM phenome (Fig. 1, Suppl. Fig. 1 of present manuscript NCOMMS-25-25126) as we also demonstrate with extensive empirical experiments (see Discussion). In addition we

compared side-by-side the ability of SAM phenome against CNN-features or CNN-LSTM-features to analyse cell morphodynamics and found that unexpectedly SAM-phenome outperforms both CNN-features and CNN-LSTM-features in classification and interpretation (Fig. 6, NCOMMS-25-25126). We have now explicitly clarified our usage of the terms "comprehensive" and "unbiased" when needed.

2) For example, your feature does not cover the acceleration of segmented objects even if you only work on 2D objects from single color 2D images

Reply:
As acceleration is the time derivative of velocity, we would expect a change in acceleration to manifest in the next time frame as a change in velocity, therefore, SPOT does implicitly capture the acceleration of segmented objects. Generally, we expect higher-order motion statistics to manifest as increasing temporal delays, requiring longer tracking to capture. One frame in a video can represent any temporal unit from 1 ms to 1 h to 1 day, depending on the video in question. Equally, the total number of frames can vary. Therefore, to ensure maximum applicability, we designed SPOT to only include motion measures that can be computed from 1 successive frame. We call this the instantaneous 'SAM phenome' and use this to map all temporal object instances into a 2D landscape using UMAP. The long-term dynamics are not lost and is modelled back through the notion of single object phenotype trajectories and phenotype cluster transitions, as we demonstrated in our analysis of single cell tracking (Fig. 5, Suppl. Fig. 5, NCOMMS-25-25126) and of cell division (Fig. 7, NCOMMS-25-25126).

3) line 169-170: how did you select the tuning parameters 200 and 3 here? Is it possible to automatically select by certain version of cross-validation?

Reply:
We want to store the segmented organoids in a more efficient manner than storing each binary pixel in individual cropped segmentation masks. However, individual boundaries have different numbers of points and would not allow stacking of multiple boundaries into a regular array, resulting in larger and irregular storage on disk. We therefore chose to sample the contour of each organoid with 200 equidistant points, as comparable numbers have been used in the literature to represent complex cell morphologies e.g. CAJAL (Govek et al., *Nat. Commun.*, 2021) which used 100 points to classify diverse neuron morphologies. To demonstrate 200 points still retains good shape approximation empirically, we assessed the symmetric Hausdorff distance of MPEG-7 shapes with increasing number of sampled equidistant contour points. We find rapidly diminishing returns in capturing the shape complexity above 100 equidistance points. This analysis is included as Suppl. Fig. 1 in the present manuscript NCOMMS-25-25126.

We partitioned the object area into 3 regions of equal distance from its boundary, as this has natural interpretation of the border, middle and central regions of an object. Unfortunately, in order for the number of total features to be identical across for all objects, the number of partitions also needs to be applicable and computable for all objects, irrespective of their area. As such, there is little scope for optimization. Whilst

further subdivision is possible, for small objects, these regions would result in smaller (and possibly zero) image area, yielding more intrinsically noisy or uninformative features. Thus we decided to maintain three regions. We also did not consider further division by angular rotation because this would require rotational alignment, which for concave shapes or shapes of high curvature may under- or over-sample the partitioned area in particular angles. Furthermore, we would require accurate rotational alignment to ensure the derived measurements are rotation-invariant – the same object but rotated is the same object and should have the same phenome. Our choice is consistent with other works such as CellProfiler (Sabatani et al., Genome Biology, 2006) and TISMorph (Prasad et al., *PLoS One*, 2019). We have included clarification of the above design choices in Fig. 1c of present manuscript NCOMMS-25-25126, which describes the development of our SAM features.

Reviewer #2

1) The manuscript is way too long and lacks focus. For me, these are at least two or three independent papers. (i) Development of the SAM tool and algorithms and testing it on the public databases. (ii) applying SAM to 2D cell culture (iii) applying SAM to 3D organoid culture. For readers, it is very difficult to extract the information that is relevant for them, and these three topics mostly have a different target audience.

2) I wonder how relevant the use of the public databases from youtube and MPEG-7 is. How relevant are these features in cell biology and using this to compare with CellProfiler seems strange.

3) Would a repository such as LIVECell not be more relevant to determine is SAM can accurately cluster distinct cellular morphologies rather than irrelevant images and videos (https://www.nature.com/articles/s41592-021-01249-6)?

Reply:
We thank the Reviewer for these constructive suggestions. Our motivation for choosing computer vision datasets was to use relatable everyday examples to demonstrate the need to design multiscale spatial features that capture shape, appearance, and motion simultaneously. We also wanted to develop a single feature set or 'phenome' that is applicable to videos of any 2D object. This enables like-for-like comparison between any analysed datasets across assays, conditions and labs.

However, following this Reviewer's advice to simplify the manuscript, during the revision, we carried out extensive work to address all three Reviewers' comments, leading us to generate two independent yet complementary companion manuscripts. The present, methods-focussed manuscript, focusses on the development and characterisation of

SAM-phenome and SPOT and their application to computer vision and 2D cell imaging data. In a companion manuscript, which we invite Reviewers to access via the Editor if necessary, entitled "*Identifying phenotype-genotype-function coupling in 3D organoid imaging using Shape, Appearance and Motion Phenotype Observation Tool (SPOT)*, we illustrate how SAM-SPOT can also be applied to analyse 3D structures such as organoids using bright field timelapse images from 2D projection images. Notably, in that paper we simulated organoid datasets with distinct predefined dynamical behaviours during the revision to comprehensively benchmark SPOT's capabilities and objectively address the Reviewers' concerns on the applicability of SPOT to analyse 2D projection videos of 3D organoids.

The present methods-focussed manuscript entitled "*Development of a universal imaging "phenome" using Shape, Appearance and Motion (SAM) features and the SAM Observation Tool (SPOT)"* contains the non-organoid information from our original submission NCOMMS-23-61673-T, with substantial expansion of the details in constructing our SAM phenome and the development of SPOT analysis framework. The present manuscript provides detailed validation of SAM-SPOT on computer vision and YouTube video datasets with ground truth. Additionally, following this reviewer's valuable suggestion, we assessed the applicability SAM-phenome and SPOT to large biological public datasets with known phenotype information, LIVECell for static images (Fig. 6, Suppl. Fig. 6, NCOMMS-25-25126) and cell division for live-cell videos (Fig. 7, Suppl. Fig. 7, NCOMMS-25-25126). We further generated data-leant convolutional neuron network (CNN)-features trained on 1 million fixed single cell images and CNN-LSTM (long short term memory)-features on >5000 single cell video frames and tested these features on >400,000 fixed single cell images and >7000 single cell video frames, respectively. The performance of SAM-Phenome was compared to data-leant CNN-features (Fig. 6, Suppl. Fig. 6, 7, NCOMMS-25-25126) or CNN-LSTM-features (Fig. 7, Suppl. Fig. 8, 9, NCOMMS-25-25126). SAM phenome outperformed both CNN-features or CNN-LSTM-features at training further phenotype classifiers, and providing more informative and interpretable explanations of results explicitly in terms of shape, appearance and motion. Our results underscore how modern deep learning methods cannot automatically learn relevant features and have many shortcomings including requiring significant expertise in architecture design and their training.

In summary, the present manuscript in addition to our companion organoids-focussed manuscript comprehensively demonstrate the power and utility of SAM-SPOT at genotype-phenotype coupling and high-content imaging screening.

4) My main concern is the images that are used for the organoids. SAM quantifies 2185 features, so it is very sensitive to imaging artifacts.

Reply:

Due to the limitation in space in our previous manuscript, we did not elucidate that SPOT's design is versatile to reconcile artefacts within the analysis. In our new MS2, this point is clarified in our detailed description of the construction of SAM phenome. Specifically, the 2185 SAM features is the entire SAM phenome and is the resultant

number of features after quantifying the parameters of shape, appearance and motion, at global, regional and local spatial scales. The high dimensionality may include redundancy across features, which builds robustness that guards against 'noise'.

We draw analogy in designing the SAM phenome to single cell sequencing and analysis vs traditional microarray/select biomarker assays. A single biomarker is interpretable but often is not always expressed nor necessarily specific to the desired cell type due to biological stochasticity and measurement noise. Using a panel is the gold standard to perform cell deconvolution and cell type calling and is much more robust. Employing the same philosophy, the SPOT workflow builds on the designed redundancy within SAM features to include many processing steps to further guard against artefacts and to maximize biological discovery. Systematic and multi-stage feature selection is employed on a per experimental basis and this is done by filtering to remove potentially unreliable features that exhibit either too high a statistical variation or that are zero-valued across all segmentations (and thus which contribute no information). Since motion (differential change over time) is one of the key parameters of SAM features, and a key advantage of live-cell imaging, a feature selection based on regression with time enables us to further identify and remove features associated with systematic errors, as imaging artefacts should not drive temporal variation in the dataset. Thus, by selecting and retaining those SAM features that exhibit temporal variation, SPOT maximizes discrimination between conditions when considering the population-averaged temporal changes or stacked bar-plots whilst minimises image artefacts. Therefore, in reality, whilst SPOT always computes 2185 SAM features initially, automated feature selection means only a fraction is adapted and used to analyse each experiment. If we instead under compute features, the information is irrecoverably lost in analysis. If we compute many more features, we suffer more from curse of dimensionality and require additional preprocessing. SPOT's preprocessing is analogous to the standard way in which only top-expressing transcripts are used to analyse single-cell experiments but the full transcriptome needs to be measured in the raw data. The advantage and the power of computing comprehensive and redundant SAM features from multiple spatial scales, the SAM phenome, versus only selected feature subsets (Suppl. Table 2, NCOMMS-25-25126) is the basis of why SAM phenome outperforms purely data learnt CNN-features both in classification and providing more informative phenotypical measurements (Fig. 6, NCOMMS-25-25126).

5) I'm wondering how much features are actually relevant. For example, for figure 3, studying the drug response in the organoids, the same conclusion can be drawn with just basic imaging features and do not require quantification of 2184 features. So for basic drug screening I think this is overkill.

We thank the Reviewer for this comment. The example given by the Reviewer concerns Fig. 3 and Suppl. Fig. 3a of our companion organoids-focussed manuscript NCOMMS-23-61673-C (included below for reference), where we tested SPOT's potential to analyze the spatiotemporal dynamics of non-cancerous patient-derived duodenal organoids grown in a 96-well plate as "normal" controls and imaged label-free. The entire SPOT

workflow was applied to measure organoid response to eight chemotherapy drugs at three different concentrations.

We agree that for a basic toxicity screen, the number of features is overabundant. However, a central premise in our manuscript is whether we can compute a universal set of SAM features in a universal manner that is equally applicable for analyzing simple (cell death) and complex scenarios (changes in organoid branching patterns). Whilst specifying a few targeted measurements such as biomarkers is simpler and more direct for a study, this is not robust and interpretation may further be confounded by conflicting results. In Suppl. Fig. 3a of our companion manuscript NCOMMS-23-61673-C (included for reference below), both Caspase and Cytotox are biomarkers of cell death and they do not fully agree with each other. We also found that we had to count the number of punctate spots instead of taking the mean intensity to get an interpretable quantification. For high-content and high-throughput applications, specifying more features then performing feature selection has proven to be far more discriminative for discovery. For example, agnostic sequencing of all available transcripts is essential for scRNAseq to discover new cell types and corresponding biomarkers compared to classical microarrays. We have now outlined more clearly the rationale for our 2185-feature approach (Fig. 1c of present manuscript NCOMMS-25-25126). We include below Fig. 3 and Suppl. Fig. 3a of our companion manuscript NCOMMS-23-61673-C, along with their legends, for reference.

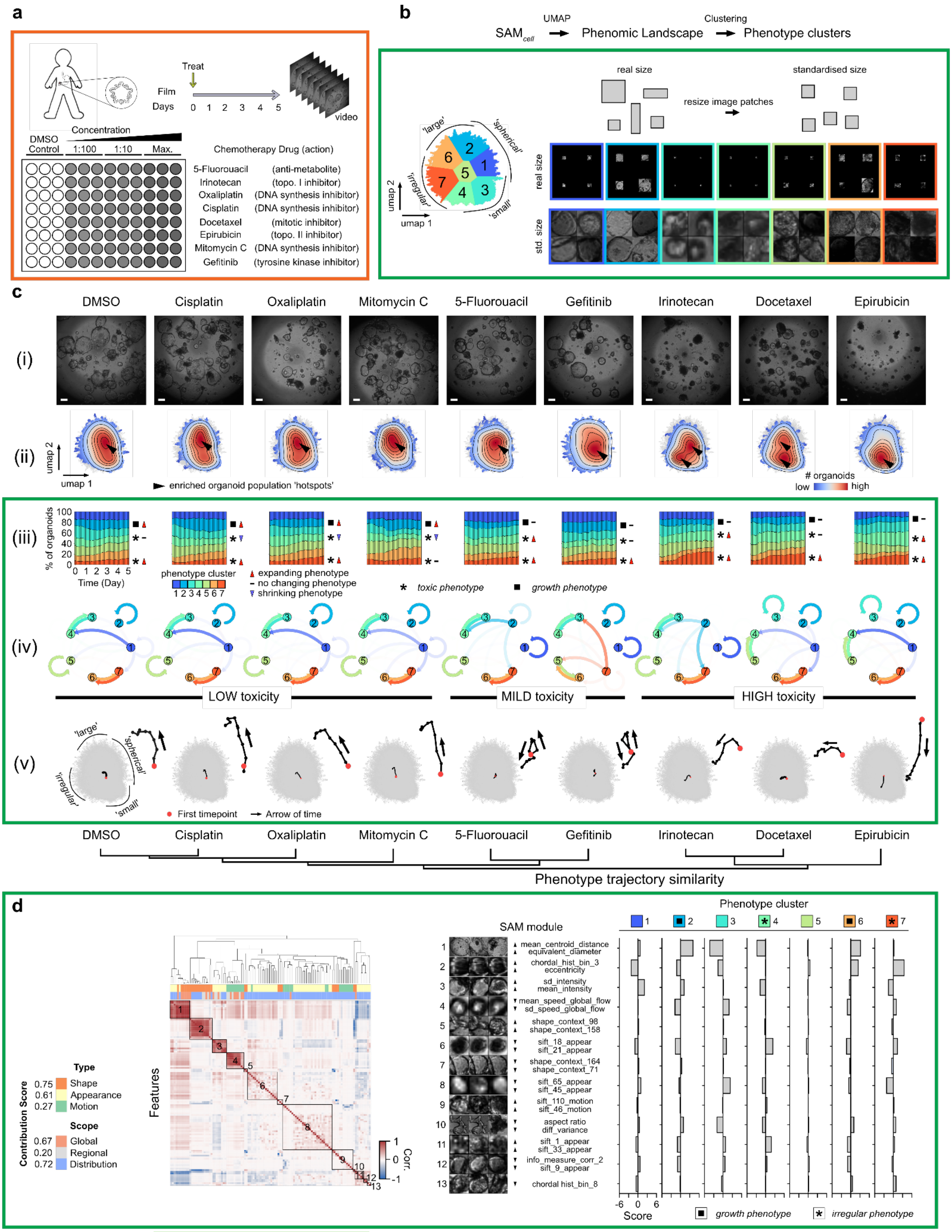

Figure 3 of NCOMMS-23-61673-C***. SPOT phenotype analysis of human patient-derived***

duodenum organoids drug response enables clustering of drugs by pharmacological action. a) *Patient-derived D2 duodenum organoids were grown from endoscopy biopsies, in a 96-well plate with the specified drug treatment and concentration, then filmed every hour for 5 days without labeling. The whole view of each well was filmed to produce one video. See methods for maximum drug concentrations. For each drug, all videos of all concentrations were pooled together for analysis.* ***b)*** *UMAP phenomic landscape using the filtered SAM phenomes from all videos across all drugs and concentrations, n=516,503 points. Each point is a segmented organoid instance from a single timepoint. Landscape is colored dark blue to dark orange and numbered in ascending order according to the mean shape eccentricity of each phenotype cluster, identified by k-means clustering and the elbow method (left). For each cluster, four representative images arranged as a 2x2 grid are shown, using their real size for comparison of scale across clusters (right, top row) and after cropping and resizing to a standardized (std.) size to show organoid appearance (right, bottom row). Illustration of real vs std. size (top right).* ***c)*** *Hierarchically clustered chemotherapy drugs according to SAM temporal phenotype trajectories using complete linkage (bottom). (i) final frame of exemplar videos for each indicated drug treatment at maximum drug concentration, scalebar: 250 µm; (ii) UMAP point density heatmaps, same UMAP coordinates as in (b); (iii) stacked barplots showing the temporal changes in phenotype cluster (as characterized in (c)) distribution for each drug, as computed from organoids at 3-hour time intervals; (iv) graph showing the Hidden Markov Model (HMM) inferred transition probability (Methods) of an organoid transitioning to another phenotype cluster in the next timepoint given its phenotype cluster label at the current timepoint. Arrows are colored by the source cluster. The more transparent the arrow, the smaller the probability of the transition. (v) UMAP SAM phenotype trajectories for a density threshold of mean+3 standard deviations (left) and zoom of the trajectory (right) for each drug. On each trajectory, each black point denotes 12-hour increments from the starting point indicated with a red point. A black arrow indicates the directionality of time.* ***d)*** *Contribution score of shape, appearance and motion, and global, regional and distributional features (left), automated hierarchical clustering to identify SAM feature modules (middle) and SAM module expression in each phenotype cluster (right) constructed as in Fig. 2f.*

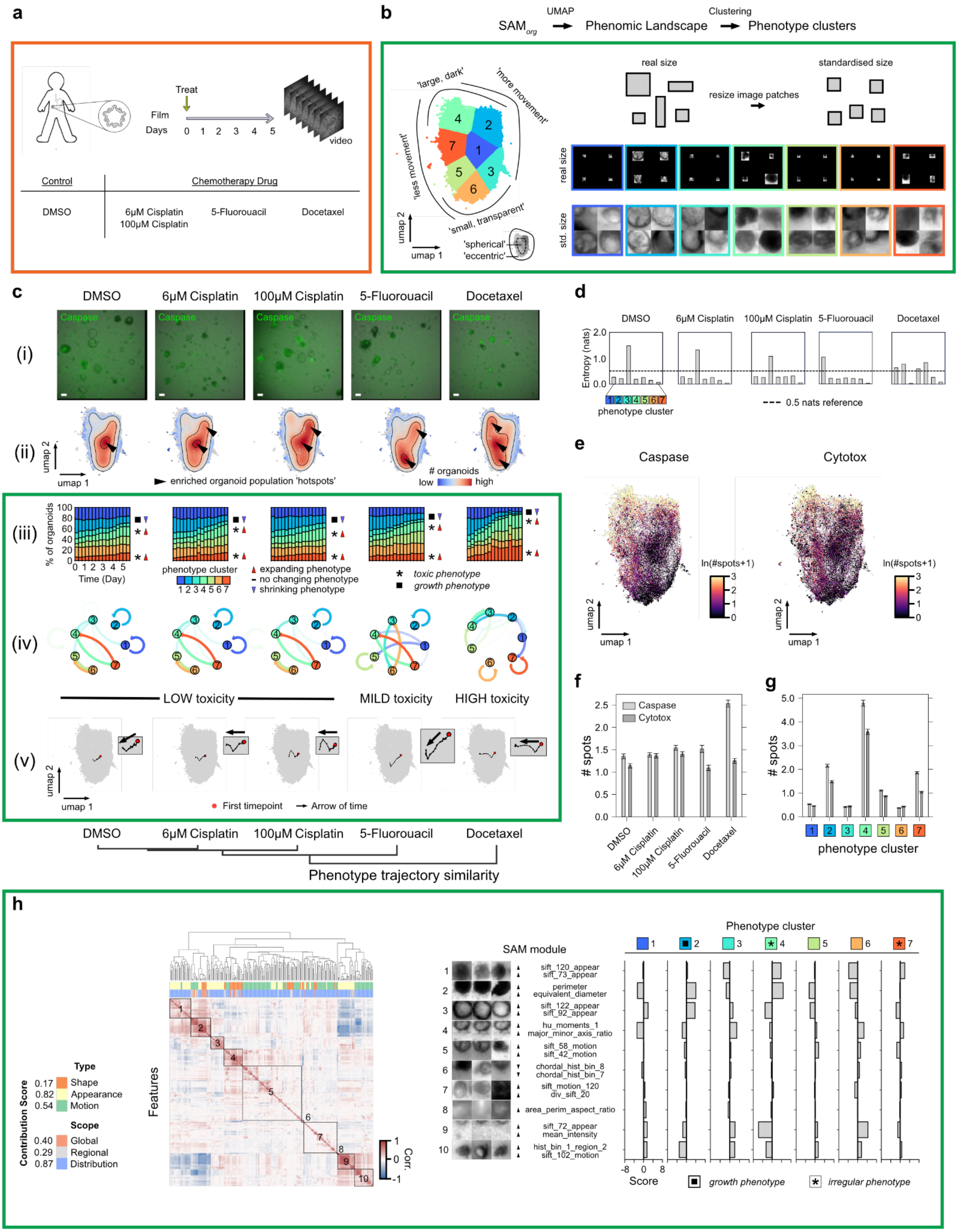
a
b
SAM$_{org}$
UMAP
Phenomic Landscape
Clustering
Phenotype clusters
Treat
Film
Days
0 1 2 3 4 5
video
Control
Chemotherapy Drug
DMSO
6µM Cisplatin
100µM Cisplatin
5-Fluorouacil
Docetaxel
'large, dark'
'more movement'
'less movement'
'small, transparent'
'spherical'
'eccentric'
umap 2
umap 1
real size
standardised size
resize image patches
std. size
c
DMSO
6µM Cisplatin
100µM Cisplatin
5-Fluorouacil
Docetaxel
(i)
Caspase
(ii)
enriched organoid population 'hotspots'
organoids
low
high
(iii)
% of organoids
Time (Day)
phenotype cluster
expanding phenotype
no changing phenotype
shrinking phenotype
toxic phenotype
growth phenotype
(iv)
LOW toxicity
MILD toxicity
HIGH toxicity
(v)
First timepoint
Arrow of time
Phenotype trajectory similarity
d
Entropy (nats)
phenotype cluster
0.5 nats reference
e
Caspase
Cytotox
ln(#spots+1)
f
spots
Caspase
Cytotox
g
phenotype cluster
h
Type
Shape
Appearance
Motion
Scope
Global
Regional
Distribution
Contribution Score
0.17
0.82
0.54
0.40
0.29
0.87
Features
Corr.
SAM module
sift_120_appear
sift_73_appear
perimeter
equivalent_diameter
sift_122_appear
sift_92_appear
hu_moments_1
major_minor_axis_ratio
sift_58_motion
sift_42_motion
chordal_hist_bin_8
chordal_hist_bin_7
sift_motion_120
div_sift_20
area_perim_aspect_ratio
sift_72_appear
mean_intensity
hist_bin_1_region_2
sift_102_motion
Phenotype cluster
Score
growth phenotype
irregular phenotype

***Supplementary Figure 3a of** NCOMMS-23-61673-C. **SPOT phenotype clusters capture different SAM characteristics of cell death after drug treatment. a)** Patient-derived D2 duodenum organoids labeled with Hoescht and additional Caspase 3/7 Green or Cytotox Green were grown from endoscopy biopsies, in a 96-well plate with the specified drug treatment and concentration, then filmed every 4 hours for 5 days. Drugs without concentration specified used the corresponding maximum concentration in Fig. 3. (Methods). **b)** UMAP phenomic landscape, phenotype clusters and representative cluster images as in Fig. 3 using the filtered SAM phenomes computed from only the brightfield channel from all videos across all drugs and concentrations, n=70,757 points. **c)** Hierarchically clustered chemotherapy drugs according to SAM temporal phenotype trajectories using average linkage (bottom). (i) final frame combined with Caspase 3/7 Green channel of exemplar videos for each indicated drug treatment and drug concentration, scalebar: 250 µm; (ii) UMAP point density heatmaps, same UMAP coordinates as in (b); (iii) stacked barplots showing the temporal changes in phenotype cluster (as characterized in (c)) distribution for each drug, as computed from organoids at 8-hour time intervals; (iv) graph showing the Hidden Markov Model (HMM) inferred transition probability (Methods) of an organoid transitioning to another phenotype cluster in the next timepoint given its phenotype cluster label at the current timepoint. Arrows are colored by the source cluster. The more transparent the arrow, the smaller the probability of the transition. (v) UMAP SAM phenotype trajectories for a density threshold of mean+3 standard deviations (left) and zoom of the trajectory (right) for each drug. On each trajectory, each black point denotes 8.5-hour increments from the origin red point. A black arrow indicates the directionality of time. **d)** Shannon entropy of the HMM transition probability for each phenotype cluster, in each drug condition. **e)** UMAP with points coloured by its number of counted punctate Caspase 3/7 (left) and Cytotox (right) spots. **f)** Mean±s.e.m number of punctate Caspase 3/7 and Cytotox spots of organoids in each drug condition. **g)** Mean±s.e.m number of punctate Caspase 3/7 and Cytotox spots of organoids in each phenotype cluster. **h)** Contribution score of shape, appearance and motion, and global, regional and distributional features (left), automated hierarchical clustering to identify SAM feature modules (middle) and SAM module expression in each phenotype cluster (right) constructed as in Fig. 2f.*

Reviewer #3 (Remarks to the Author):

1) The paper describes an extension to the toolbox of researchers that appears to outperform the established standard, namely CellProfiler. The authors argue that the introduction of motion in their pipeline, and a determination between global, local-regional and pattern-pixel levels are key differentiators for success. The authors support this statement well in text and figures, namely through the reporting of contribution scores for their clustering interpretation.

We thank the reviewer for their positive comments.

2) Overall, the paper is very well written, providing a new toolbox to researchers that has the potential to become of significant value to the field.

We thank the reviewer for their comments and for highlighting the value that our technology will bring to the field.

Appendix – Methods section of companion organoids-focussed manuscript NCOMMS-23-61673-C entitled *"Identifying phenotype-genotype-function coupling in 3D organoid imaging using Shape, Appearance and Motion Phenotype Observation Tool (SPOT)"* detailing the methods behind simulated organoids work referenced in the present point-by-point response to Reviewers' comments.

Simulating organoids with prescribed 3D shape, appearance and motion dynamics

We generated simulated organoid datasets with full control of the 3D phenotypic heterogeneity within and between treatment conditions. By design, this dataset has ground-truth for objectively assessing SPOT analysis performance in high-content organoid screens. Custom shape, appearance and motion parametrization of each organoid within a condition establishes that all ground truth phenotype states are distinct, while the uniqueness of each individual organoid is maintained by sampling its parameters from Gaussian distributions. For each condition, all parameter values are made available in the associated metadata files at DOI: 10.5281/zenodo.14265519 and in Suppl. Table 5 (please request access to Suppl. Table 4 to the Editor, if necessary).

To simulate shape evolution, we generated an initial 3D organoid shape as a surface mesh, and deformed the mesh in successive timepoints. To generate the initial shape we start with a spherical icosphere mesh; sample surface coordinates to locate organoid branches; and then creating the organoid branches by deforming the local icosphere mesh vertices around to each coordinate with the same effect as placing a 3D ellipsoid with major axis oriented perpendicular to the surface. Organoid growth is simulated by isotropic scaling of mesh vertices from the organoid centroid. Then, branch growth is simulated by displacing branch-associated vertices further from the organoid centroid, with the same effect as stretching along its major axis, a 3D ellipsoid with major axis oriented perpendicular to the surface. To simulate a gradual shape evolution, we create a 3D final organoid shape similarly retaining the branching position and number of branches. We then linearly interpolate organoid and branch growth-associated parameters between initial and final shapes for intermediate timepoints.

To simulate appearance evolution, voxel-based intensity patterns were generated and intersected with the inner volume of the evolving 3D organoid meshes at each timepoint. 2D projections of the composite shape and appearance 3D simulations were produced by sampling the 3D organoid at multiple 2D planes and generating a single 2D image in an analogous way to extended depth-of-focus algorithms. The synthetic organoid boundary sampled in each 2D plane was smoothed by fitting a Bezier curve, with mesh-plane intersections serving as control points.

To simulate motion we translated and rotated organoids. For translation, at each timepoint, we sample two random vectors with their magnitude and direction (represented by a unit

vector) independently sampled from Gaussian distributions, representing 'migration' and 'jitter', to displace the organoid centroid. The 'migration' vector represents longer-time persistent directional movement. At each timepoint, we determine with probability $\frac{1}{p_{migrate}}$, where $p_{migrate}$ can be interpreted as number of frames motion persists unidirectionally, whether to change its direction by sampling a new unit Gaussian vector. The 'jitter' vector represents stochastic movement between timepoints with no net directionality. A new direction unit vector is sampled at every timepoint. For rotation, at each timepoint we rotate the organoid by 3^0 about a rotation axis passing through the organoid centroid and represented by a unit Gaussian random vector. The rotation axis is modelled with longer-time directional persistence as with the migration vector. At each timepoint, we determine with probability, $\frac{1}{p_{rotation}}$ where $p_{rotation}$ can be interpreted as the number of frames rotation persists in the same direction, whether to change rotation axes by sampling a new unit Gaussian vector.

To simulate a gradual motion evolution we linearly interpolated magnitude parameters of initial and final states for intermediate timepoints. The same probability of migration and rotation changing direction between timepoints were used for all simulated conditions.

We used the described SAM simulation strategy to generate two datasets of organoids for validating SPOT analysis where each organoid is unique with a different shape, appearance and motion parameterization and categorized by a distinct SAM phenotype.

Simulated Dataset A for assessing the impact of imaging artefacts and processing errors

Dataset A comprises 7 conditions, 48 organoids per condition, and 30 frames per organoid. Each of the conditions share a common starting state with simulation parameter values sampled from Gaussian distributions (see above), before gradually evolving over the 30-frame time course into their uniquely-parameterised, and condition-specific end SAM state. However, 'appearance-high' and 'appearance-low' were simulated with a higher-density appearance pattern to exacerbate final state differences. We define one condition-dependent end state as the 'base organoid', with average parameter values of shape, appearance and motion. Using this base organoid parameterisation as template, and modifying the parameters influencing each of shape, appearance, and motion in isolation, we created the other 6 further 'high' or 'low' extremal SAM phenotypes. Simulated dataset A is made available with individual 3D organoid meshes and associated 2D projection videos at DOI: 10.5281/zenodo.14265519.

Perturbing Dataset A to mimic imaging artefacts and errors in segmentation and tracking

We perturbed Dataset A to investigate the impact of three primary categories of errors on SPOT analysis (Suppl. Fig. 2c): 1. imaging-based artefacts including: (i) blur, (ii) overexposure, (iii) underexposure, and (iv) foreign-object artefacts; 2. segmentation errors including: (v) overlap between organoids, (vi) partial segmentation of an organoid (cutout), and (vii) multiple organoids segmented as one (union); and 3. tracking error, namely (viii) track fragmentation as a result of incomplete tracking due to occlusion. These perturbations were simulated with across nine increasing severity levels as follows:

(i) Blurring

2D projection images of individual organoids were Gaussian blurred at increasing sigma (level 1: $\sigma = 2$, level 9: $\sigma = 14$) using the GaussianBlur function from the OpenCV library.

(ii) Overexposure

8-bit 2D projection images of individual organoids were overexposed by increasing the image brightness, I by a fixed amount, γ.

$$I_{overexposure} = I + \gamma$$

Any pixels with resulting brightness greater than 255 is clipped to 255, the maximum intensity of 8-bit images and represents camera saturation. The added amount increases linearly with severity level (level 1: $\gamma = 20$, level 9: $\gamma = 180$)

(iii) Underexposure

8-bit 2D projection images of individual organoids were underexposed by decreasing the image brightness, I by a fixed amount, γ.

$$I_{overexposure} = I - \gamma$$

Any pixels with resulting brightness lower than 0 is clipped to 0, the minimum intensity of 8-bit images. The decreased amount increases linearly with severity level (level 1:$\gamma = 10$, level 9: $\gamma = 90$).

(iv) Foreign-object artefacts

We simulated two types of common object artefacts that may be imaged but unlike organoids do not temporally evolve. The first artefact is small-sized pixel-scale objects, such as fine dust. We simulated these as ‘salt' noise in the 8-bit 2D projection images, where we replace the value of a randomly selected pixel with 255, the maximum brightness. We increase the fraction of image pixels to replace with noise using the random_noise function from scikit-

image linearly for increasing severity (level 1: amount = 1/6000, level 9: amount = 9/6000). The upper amount was qualitatively determined to represent high noise at a realistic density. The second artefact is larger-sized fibre-like debris such as hair or clothes fibres. We simulate these with Bezier curves and placed them in random spatial locations in the image. Bezier curve thickness and length were chosen to resemble clothes fibres at organoid-scale. Increasing severity was simulated by increasing the number of fibres proportionately (level 1: $n = 15$ *, level 9:* $n = 135$ *fibres). The simulated dust and fibre artefacts are randomly generated for an organoid and remains identical in every timepoint.*

(v) Overlapping organoids

We simulate the effect of correctly segmenting individual organoid boundaries but with internal appearance differences due to intensity interference from overlapping with a neighbour organoid. Intensities in the overlapped area were simulated by pairing organoids from the same condition and timepoint, aligning them horizontally with a degree of overlap, then summing pixel intensity values and clipping the result to be within the 8-bit range. Severity level corresponds to increased area of overlap; we fix a baseline level of overlap at 40 pixels, beyond which additional overlap is added as a percentage of the remaining non-overlapping area (level 1: 0%, level 9: 100%).

(f) Cutout

When organoids overlap, the segmentation may erroneously segment only the non-overlapped area, resulting in a smaller 'cutout' of the actual shape. We simulate this by pairing organoids from the same condition and timepoint, aligning them horizontally with a degree of overlap, then subtracting the overlapping area from each of the organoids. Severity level corresponds to increased area of overlap; we fix a baseline level of overlap at 40 pixels, beyond which additional overlap is added as a percentage of the remaining non-overlapping area (level 1: 0%, level 9: 100% overlap).

(g) Union

When organoids overlap, the segmentation may erroneously regard two organoids as one. We simulate this effect by pairing organoids from the same condition and timepoint, aligning them horizontally with a degree of overlap, and taking their union to be a single instance. Severity level corresponds to increased area of overlap; we fix a baseline level of overlap at 40 pixels, beyond which additional overlap is added as a percentage of the remaining non-overlapping area (level 1: 0%, level 9: 100% overlap).

(h) Fragmented tracks

We define the perfect baseline ground truth as 100% coverage whereby each individual organoid is 100% tracked for all 30 frames. To generate fragmented tracks, we fixed coverage at 50% i.e. a total of 15 frames is tracked per organoid, and at each increase severity level we increase the number of synthetic breaks by one. For example at level 1, the organoid has one tracklet, covering 50% of frames, but at level 2 has two tracklets, each covering ≈25% of frames, such that the total is still 50%, at level 3 three tracklets, etc.

Measuring the impact of perturbing Dataset A to SPOT analysis

Taking the unperturbed Dataset A as severity level 0, for each perturbation type (a)-(h) above, we applied SPOT, preprocessing all SAM phenomes together to derive a joint UMAP phenomic landscape pooling all organoids across levels 0-9. We then assessed the impact of each perturbation type on SPOT analysis by computing metrics of agreement and disagreement in the four primary SPOT outputs: (i) phenotype clustering agreement; (ii) stacked barplot disagreement, (iii) phenotype trajectory disagreement, and (iv) HMM transition matrix disagreement, between each severity level to the unperturbed dataset A (ground-truth).

(i) Phenotype clustering agreement

K-means with automatic cluster number selection using the elbow method was applied to partition the joint UMAP into phenotype clusters. Two cluster metrics: adjusted random index (ARI) and adjusted mutual information (AMI) were used to assess the agreement in the phenotype cluster assignment at each severity level compared to the unperturbed. ARI and AMI scores agreement on scale from 0 (perfect disagreement) to 1 (perfect agreement).

(ii) Stacked barplot disagreement

We model the stacked barplot as a 2D probability distribution, and measure the disagreement to ground truth using the Wasserstein optimal transport metric. Specifically we compute the sliced Wasserstein metric (max_sliced_wasserstein_distance implemented in the Python POT library) using uniform weights and the matrix representations of the ground truth and perturbation stacked barplot data as the samples, stabilising the estimate by setting n_projections = 50. This metric enables us to score disagreement based on intuitive ‘pattern similarity’ of the cluster frequency. Thus, the same cluster pattern but temporally lagged will have lower disagreement than a cluster pattern with expansion of one cluster and contraction of another cluster.

(iii) Phenotype trajectory disagreement

We measure whether the similarity between phenotype trajectories of each condition in dataset A given by their computed trajectory affinity matrix with the joint UMAP is preserved

at each severity level. The disagreement at severity level i, is the mean of the absolute elementwise difference between the trajectory affinity matrix of the unperturbed dataset and that at level i.

(iv) HMM transition matrix disagreement

We measure the mean of the absolute elementwise difference between the HMM cluster transition matrix for the unperturbed dataset and that at each severity level 1 to 9.

These metrics were assessed and plotted in Suppl. Fig 3c for all perturbation types. Tracking error represented by fragmented tracks used the same phenomic landscape constructed by the unperturbed dataset A. Phenotype cluster agreement was therefore not assessed. To compute the stacked barplot, trajectory and HMM transition disagreement metrics, the subset of organoid UMAP coordinates corresponding to the fragmentation were used at each level.

POINT-BY-POINT REPLY TO REVIEWERS:

Reviewer #1 (Remarks to the Author):

I don't agree with the split of the original paper to two.

Author's reply:

We recognise the reviewer's concerns regarding the division of this submission into two, separate companion papers. After careful consideration, taking into account the Editor's comments, we feel that retaining the two manuscripts helps to convey the content more clearly to the two different readerships we anticipate:

1) Methods-oriented readers who are primarily interested in features and comparison to deep learning methods, and

2) Organoid phenotypic-screening communities, interested in applying a high-content analysis tool for label-free imaging.

Reviewer #1 (Remarks on code availability):

I cannot run it successfully

Author's reply:

We retested the code and found

1. The code dependencies were not compatible with Python >= 3.11. It works for Python 3.9 and 3.10. We have updated the readme, and also encoded the constraints in the pyproject.toml setup file
2. We have created a step-by-step install video with annotations to help users get setup and running the example scripts. The video is available at this link, and we have highlighted this in the code repository at https://github.com/fyz11/SPOT.

Reviewer #2 (Remarks to the Author):

I would like to congratulate the authors on the substantial improvements made to their manuscript. Splitting the original version into two separate manuscripts has significantly improved the clarity and readability of the work, making the core concepts and contributions more accessible to the reader.

Author's reply:

We thank the reviewer for recognising the improved clarity of our two manuscripts.

The addition of more biologically relevant datasets, particularly the inclusion of the LIVECell database, greatly enhances the manuscript's applicability to real-world cell-based imaging studies. I found the inclusion of machine learning classifiers to predict cell lines and mitotic states particularly compelling. These results convincingly demonstrate that SAM-SPOT extracts phenotypically meaningful and discriminative features

that can be used for accurate classification.

Author's reply:

We thank the reviewer for their positive comments on our revised work. Building on this, we have now added further classifier benchmarks for LIVECell and the timelapse HeLa mitosis datasets to DINOv3, a state-of-the-art computer vision feature extractor, and O2-VAE, a state-of-the-art cell biology feature extractor. We show that SAM-SPOT features also outperform these state-of-the-art deep learning feature extractors on the same datasets.

I'm particularly curious about how SAM-SPOT would perform in the context of cells exposed to a diverse panel of compounds with known, distinct, or overlapping mechanisms of action. It would be interesting to explore whether SAM-SPOT can effectively stratify such compounds based on the dynamic phenotypic responses it captures. But that does not need to be part if the current manuscript.

Author's reply:

Thank you. We agree with the reviewer and will explore this in future work.

Reviewer #4 (Remarks to the Author):

I am confused as the authors seem to have two versions of the same article. One named "Development of a universal imaging "phenome" using Shape, Appearance and Motion (SAM) features and the SAM Observation Tool (SPOT)" and one this one that I am writing review for now. I have already wrote reviews for the "developement ..." version and I will paste it here as well. My only new comment for this current version "Identifying ..." is that this version seems to have more support from biological and simulated data but lacks the comparison to the state-of-the-art. Why did not the authors submit one version with all three in place (biological verification, simulated experiments, literature comparison)? That would have been best in my opinion.

Author's reply:

Thank you for the observation.

Our initial submission was a combined single manuscript. However, following reviewer 2's comments and during the review process, it was felt that the joint paper lacked focus and was too long for a general readership. Consequently, we split the manuscript into the current two manuscripts: one focussed on introducing the analytical framework, and benchmarking its performance on public datasets with emphasis on single cell imaging; and the second demonstrating the ability to adapt the framework to analyze 3D organoid dynamics through 2D projection videos, introducing a new organoid simulation dataset in the process.

We feel that the separation of these manuscripts adds greater clarity, and caters towards the two different general readerships that would be interested in our work: the tool developers and the organoid community.

The below is the same comments as I had for the "Development ..." version of the paper which are relavent

for the current version, i.e., "Identifying ..." too. I do not have the biological knowledge to verify any biological verification that the "Identifying ..." version claims, as I am a computer scientist.

1. What are the noteworthy results?

***[Answer from the reviewer to the Editor]** The authors claim that a universal set of 2185 image-derived features is developed to quantify an object's phenotype ("standard" image-based "phenome"); subsequently, they provide a tool for image "sequencing" analysis of phenomes. For temporal analysis, they compute the HMM phenotype cluster transition graph that shows the different state of each detected phenome cluster over time.*

2. How does it compare to the established literature?

***[Answer from the reviewer to the Editor]** I. In the introduction, the authors mention two previous feature descriptors, CellProfiler and TISmorph. But they only compare to Cellprofiler. The comparison is not enough. The authors should compare to TISmorph36 too.*

Author's reply:

We have added additional comparison to TISmorph using the official code (https://github.com/Elaheh-Alizadeh/Quantifiction-of-shape-and-structure) in the methods paper (NCOMMS-25-25126A) for the analysis of MPEG-7 shapes (new Suppl. Fig. 3b a of NCOMMS-25-25126A), Normalized Brodatz textures (new Suppl. Fig. 3b b of NCOMMS-25-25126A) and Brodatz textures MPEG-7 shapes (new Suppl. Fig. 3b c of NCOMMS-25-25126A). As can be seen, TISmorph is the worst performing. For Brodatz textures MPEG-7 shapes, we further assessed the supervised classification performance with respect to the size of the training split (Suppl. Table 3). We see SAM-SPOT is the best overall. However, if there is insufficient training data i.e. <5%, the feature set with lower dimensionality is more superior. Since generally, we use >50% training data, SAM-SPOT is the superior feature set.

II. The comparison is limited to two methods, i.e., merely done for CNN and CNN-LSTM based autoencoder. The authors should compare to more methods e.g. generative models and graph-based models. Two examples of such methods: A) Dynamic graph convolutional foldingnet in "3D single-cell shape analysis of cancer cells using geometric deep learning", B) "DeepSea is an efficient deep-learning model for single-cell segmentation and tracking in time-lapse microscopy". Then given their claim of rotation-invariance, they should compare to different VAE tackling rotation-invariance and not just a naive auto-encoder: C) "ME-VAE: Multi-Encoder Variational AutoEncoder for Controlling Multiple Transformational Features in Single Cell Image Analysis"

Author's reply:

We thank the reviewer for the suggestions of possible additional benchmarks. After further research, we decided to benchmark SAM/SPOT against two state of the art methods; DINOv3 and O2-VAE rather than the methods suggested by this reviewer for the following reasons:

a) Dynamic graph convolutional folding net (PMID: 40112779) was developed for i) shape-only features and ii) 3D point-cloud only inputs. Our work, SAM-SPOT is applicable currently only to 2D videos. In our organoid paper, we project 3D z-stacks to 2D videos for analysis.

b) DeepSea (PMID: 37426758) is only developed for segmenting and tracking organoids. SAM-SPOT doesn't claim novelty in the segmentation and tracking, which are based on training already published neural network architectures. These tasks are not the goal of SAM-SPOT which aims to be a pipeline for analysis after you have segmented and tracked organoids/cells. The segmenter and tracker we provide work well for the diverse datasets we have tested and is provided for user convenience to accomplish these two prerequisite tasks. Indeed, we have designed SAM-SPOT to be modular, and encourage users to utilise their own segmentations and tracking if they are better suited to their particular use case.

c) ME-VAE: Multi-Encoder Variational AutoEncoder (PMID: 35322205) – We agree that this framework would be an interesting benchmark. However, we found that the official code repository, https://github.com/GelatinFrogs/ME-VAE_Architecture does not provide code to create the data transformations for custom training and it would be unfair to benchmark ME-VAE with any custom data transformer we would have to write as replacement. Instead, we found an alternative, O2-VAE (PMID: 38310122) that in the same spirit, aims to learn equivariant features. Different to ME-VAE, O2-VAE uses an orientation-invariant convolution encoder, instead of relying on explicit data transformations (https://github.com/jmhb0/o2vae), allowing us to retrain the network.

The motivation of using CNN and CNN-LSTM was to create baseline methods where the same encoder architecture could be used for both fixed and temporal imaging.

We have now added additional classification benchmarking of LIVECell and HeLa cell division datasets to the following two state-of-art architectures, chosen to represent the two current major lines of research in feature engineering: i) self-supervision, ii) equivariant learning:

1) DINOv3 (https://github.com/facebookresearch/dinov3) – This family is state-of-the-art in self-supervised deep learning for general computer vision and one of the most popular benchmarked also for cell imaging (e.g. PMID: 39929956).
2) O2-VAE (https://github.com/jmhb0/o2vae) – This recent state-of-art cell feature learning VAE network uses prealignment and O(2)-equivariant steerable CNN layers to ensure equivariant feature learning.

We trained the DINO networks using as close to the same number of features as SAM-SPOT as we could not vary this, and trained O2-VAE with the same number of features as SAM-SPOT (in alignment with this reviewer's later comment on maintaining the same number of features). With DINOv3 we could not directly specify the number of features, therefore we used the version with 768 features and then performed L2 penalized linear support vector machine to select a number of top-performing features to match SAM-SPOT. Both networks are designed only for fixed imaging. Therefore, similar to CNN-LSTM, to adapt to the temporal dataset, we first computed the DINOv3 or O2-VAE features for individual video frames then trained a multi-layer perceptron (MLP) encoder with bidirectional LSTM incorporating the same 5-frame time window information as the CNN-LSTM. The temporal features were then the encoded features of the MLP encoder whose number was matched to the number of SAM features.

Our benchmarking results on supervised classification find SAM-SPOT features as the best performing (Suppl. Table 3,4). Moreover, we found the more state-of-the-art networks did not perform much better than CNN and CNN-LSTM features.

3. Does the work support the conclusions and claims, or is additional evidence needed?

[Answer from the reviewer to the Editor] *I. In suppl. Fig. 8 the number of features differ between SAM and CNN-LSTM. What happens if the number of features set to be the same? That way it may be more fair to compare. The same should be done for the table of results comparing the accuracy of CNN and CNN-LSTM (right now SAM has not the same number of features as the other two).*

Author's reply:

We reperformed the comparison, setting CNN and CNN-LSTM feature to match the number of SAM features in the LIVECell and HeLa cell division datasets. We find little difference in classification performance. We find the same behavior in SPOT analysis with the neural network features not fully capturing the phenotypic diversity. In sum, SAM still greatly outperformed the CNN and CNN-LSTM features.

II. In all illustrations of HMM cluster transition probability, it looks like a state machine rather than a probabilistic state machine (HMM. The author should annotate what each edge coloring represents as a probability.

Author's reply:

Yes, the cluster transition probability is a state machine. To avoid confusion, we have now removed the HMM analysis. The cluster transitions are estimated as a "state machine", based on the observed occurrences of the phenotype cluster in the next timepoint, given the phenotype cluster in the current timepoint:

$$\Pr(\text{cluster}(t+1) = j \mid \text{cluster}(t) = i) = \frac{(\#\text{cluster}(t+1) = j | \text{cluster}(t) = i)}{\Sigma_j (\#\text{cluster}(t+1) = j | \text{cluster}(t) = i)}$$

With this model, however, we found self-cluster transitions i.e. cluster *i* to cluster *i* to dominate the transition probability. Therefore, we now plot the transition graph for the relative inter-cluster transition probability (row-normalized transition matrix after removing the diagonal self-transition entries):

$$\Pr(\text{cluster}(t+1) = j \mid \text{cluster}(t) = i, j \neq i) \in [0,1] = \frac{(\#\text{cluster}(t+1) = j | \text{cluster}(t) = i, j \neq i)}{\Sigma_{j \neq i} (\#\text{cluster}(t+1) = j | \text{cluster}(t) = i, j \neq i)}$$

The arrow is drawn from the source cluster in the current timepoint to the target cluster in the next timepoint. To make this clear, the arrow is colored by the source cluster. The solidity (i.e. transparency) of the arrows' color reflects the magnitude of this probability (see figure below). This information is included in the figure legends. Specifically, to enhance contrast with the revised Markov model, we graph the probability raised to an exponent, γ, $(\Pr(\text{cluster}(t+1) = j \mid \text{cluster}(t) = i, j \neq i))^{\gamma}$ with a minimum value cutoff and a maximum value cutoff. If not stated in-text, $\gamma = 3$, min. cutoff=0, and max. cutoff=0.6. The arrow widths are all the same.

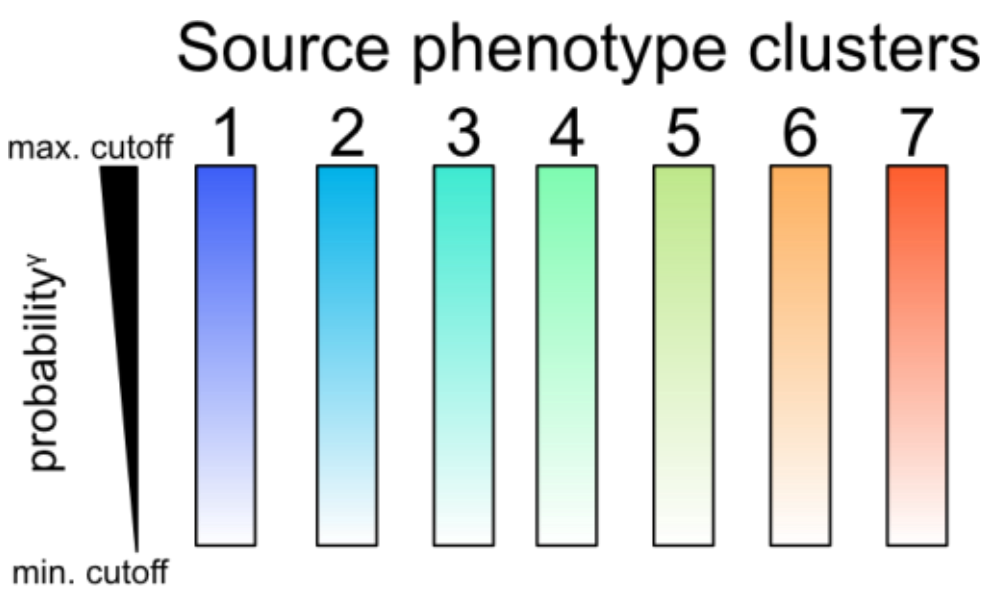

III. See points 2 and 5 and address them too.

Author's reply:

It seems to the authors that these two comments (2. how does it compare to established literature and 5. Is the methodology sound? Does the work meet the expected standards in your field?) were the questions from the editor to reviewer 4. Therefore, we felt it best to leave the reviewer 4 to address these comments. However, as reviewer 4 has made specific comments in relation to point 5, we have provided some specific replies to the comments.

From our point of view, we of course feel strongly that SAM/SPOT represents a sound, state of the art technology in the field and will transform our ability to quantitatively capture valuable information from live imaging.

4. Are there any flaws in the data analysis, interpretation and conclusions? Do these prohibit publication or require revision?

[Answer from the reviewer to the Editor] *I cannot see any.*

Author's reply:

We thank the reviewer for their consideration.

5. Is the methodology sound? Does the work meet the expected standards in your field?

[Answer from the reviewer to the Editor] *I. I do not understand their HMM. I thought first Kmeans is used to find 7-8 clusters and then for "each cluster" the temporal analysis is done (this is what the authors claim in line 1319 section). This makes sense to me. However, in their HMM transition (shape of state machine with color-coded arrows), I see that they show transition between those 7-8 clusters. Why this? and why not the state transition for each cluster separately? If the author answer this as "we want to see transition between different clusters" then my judgment is that their modeling is not sensible - they should NOT first do Kmeans to detect states of an HMM (the whole point of HMM is to find those states). Instead, they should have directly applied HMM and then Viterbi to find the most probable sequence, which would unfold the trajectory of states - the states would be detected by fitting the HMM and would not need to have K-means in advance.*

Author's reply:

We agree with the reviewer that having found our phenotype clusters our aim was to "see transition between different clusters". Specifically, we wanted to estimate the probability of an organoid being of phenotype cluster *i* transitioning to phenotype cluster *j* in the next timepoint. We did not want to explicitly link the hidden states of a HMM with the definition of phenotype clusters as this would restrict the application our pipeline to strictly longitudinal data. In our current design, phenotype clusters can be found for both fixed and live-cell imaging. To avoid confusion of the use of HMM in our data analysis, we have now replaced HMM with the explicit and simplified approach of direct computing the desired cluster transition probabilities directly as a "state machine".

Specifically, we use Kmeans to find automatically a discrete number of clusters, typically 7-8 clusters which partition the UMAP phenomic landscape. Each cluster then represents a prototypical set of SAM characteristics.

We then use the clusters to go back to the single-object tracking information to assign to each object instance in the track its phenotype cluster label. This gives a cluster label trajectory e.g. 1,1,1,1,1,2,2,2,2,2 where the object was tracked for 10 timepoints, initially being cluster 1 and then transitioning to cluster 2.

Within our cluster transition analysis, we aim to estimate the probability that an object instance is phenotype cluster *j* at time *t+1,* given that it was phenotype cluster *i* at time *t.*

We want to highlight that we regard i) finding the phenotype clusters, and ii) finding the cluster transition probability as two independent and separated tasks. The advantage of this is that task i) is still applicable when we have fixed imaging (non-temporal) data. We do not want i) to be linked to the hidden states of a HMM.

We apologise that the HMM has caused confusion in the interpretation of our methodology. As mentioned above, in our revision, we now compute the desired cluster transitions directly as a "state machine", from the observed occurrences of the phenotype cluster in the next timepoint, given the phenotype cluster in the current timepoint, pooling across all tracked objects in the analyzed dataset:

$$\Pr(\text{cluster}(t+1) = j \mid \text{cluster}(t) = i) = \frac{(\#\text{cluster}(t+1) = j|\text{cluster}(t) = i)}{\Sigma_j(\#\text{cluster}(t+1) = j|\text{cluster}(t) = i)}$$

For label smoothing, we provide the users the option of performing a sliding window majority filter. Each label trajectory will be scanned with a sliding window of size *w* and the output is the majority label in the window. Activating this option smooths sporadic, short-lived transitions such as the 1,2,1 transition in the sequence 1,1,1,1,1,2,1,2,2,2,2.

II. The authors should provide answer to this: why SAM? I am not sure if motion is sufficiently captured since it is "Motion as combined changes in the object's shape, appearance, and centroid between consecutive timepoints". That is, why not separately consider rotation and other potential spacial variations which can change the perception. See, for example, "ME-VAE: Multi-Encoder Variational AutoEncoder for Controlling Multiple Transformational Features in Single Cell Image Analysis".

Author's reply:

Computer vision refers to shape as boundary, edge, or contour features, appearance as texture features and motion as optical flow features. The purpose of SAM was to capture instantaneous phenotype and we consider SAM as three key conceptual components to an image. Each provides unique and complementary information as illustrated with the cartoon example of a bird in Fig. 1b of the methods paper (NCOMMS-25-25126A). This is not a new concept, and forms the basis of computer vision prior to deep learning. Computer vision refers to shape as boundary, edge, or contour features, appearance as texture features and motion as optical flow features.

The need for all three sets of features is evidenced by i) classification performance in YouTube Dataset; and ii) the variation contribution of shape, appearance and motion features, and the categorization of top features in each SAM module which changes for each analyzed dataset. If consecutive time motion is uninformative i.e. its inclusion doesn't contribution any information to the analysis, then using motion features should not contribute any improvement in classification performance, or dataset variance.

This is not however what we observe:

i) YouTube classification performance:
Suppl. Table 2 in NCOMMS-25-25126A Methods paper highlights how motion features is the best performing single set of features, whilst shape features are the worst. Moreover, the joint SAM outperforms all single feature sets.

ii) Variation contribution and top performing features in SAM module analysis:

- Fig. 5 in our Methods paper NCOMMS-25-25126A shows U373 cells which predominantly migrate and change shape. Correspondingly, we measure contribution scores of 0.60 Shape, 0.20 Appearance and 0.78 Motion. Moreover, most of the SAM modules (8 of 9) are dominated by motion-based features, those prefixed with 'div_sift_', 'curl_sift' and 'speed_reg'.
- Suppl. Fig. 5 in our Methods paper NCOMMS-25-25126A depicts the opposite extrema of HL60 cells dividing in-place with little to no migration. Consequently, we measure contribution scores of 0.59 Shape, 0.78 Appearance and 0.22 Motion. Likewise, few modules (3 of 16) are driven by motion-based features.

We would like to reiterate that the purpose of SAM was to capture the instantaneous phenotype. We agree that to "fully capture motion behavior" e.g. the full migratory sequence of a cell, we should compute features over a longer time interval. However, it is unclear how long this time interval should be. We would then be reverting back to the standard trajectory analysis framework. Conceptually, this would no longer be instantaneous information. For maximum generality, we therefore consider only time, *t* to time *t+1* drawing parallels to RNA velocity (PMID: 30089906). We note however this formulation does not specify the length of actual time elapsed between two timepoints. Therefore, to increase temporal coverage, one can perform autocorrelation analysis to set a minimal duration between timepoints, to have maximum temporal information (i.e. two near-identical video frames offer minimal additional information). In the analysis of mouse organoid branching in new Suppl. Fig. 3 b-d (Organoids paper, NCOMMS-23-61673C), we used every other frame, increasing the time duration between timepoints from 15 mins to 30 mins.

Our motion features is depicted in Fig 1c, 3rd column, Methods paper. We refer to "Motion as combined changes in the object's shape, appearance, and centroid between consecutive timepoints" because our motion features are derived from the computed optical flow ("the pattern of apparent motion of objects") between successive timepoints, and this calculation involves the image intensities (c.f. Farneback dense optical flow), producing a vector per pixel. Our motion features inherently consider rotation and other potential spatial variations of this vector field. Specifically, the same pattern rotated by X degrees, should produce similar features. Whereas ME-VAE uses averaging over explicit data transformation groups to obtain invariance, here in SAM-SPOT, as with O2-VAE, and SIFT, the invariance is built into the computation directly which uses i) magnitude of the vector fields, and ii) permutation-invariant operations like mean and histogram or iii) invariant features like SIFT (scale-invariant feature transform, *D.G.Lowe, "Object Recognition from Local Scale-Invariant Features" (1999)*).

Specifically, breaking it down:

- <u>Mean boundary and optical flow speed statistics</u> are invariant: using the magnitude and the mean which are permutation invariant.
- <u>Mean regional optical flow statistics and histograms</u> are invariant: bands are radial and therefore rotational-invariant, mean and histogram over speed are invariant operators (no orientational ambiguity).
- <u>Speed, Curl and Divergence SIFT features</u> are invariant: we use the scalar magnitude of these quantities as 'intensity images' and compute their SIFT features. SIFT features are well-known to be scale and rotation-invariant.

We note in revision, Fig. 1c (Methods paper, NCOMMS-25-25126A) did not explicitly specify 'curl magnitude' and 'divergence magnitude'. We have added additional clarifying texts to avoid any ambiguity.

Finally, a way to convince reviewers is to show an example of "rotation" and an example of "polar orientation" that is correctly detected using their method, and it is not confused and perceived as a change in shape.

Author's reply:

We thank the reviewer for suggesting the additional benchmarking, and the new results have resulted in improved ways of computing SAM features. All these are now included in our revised methods and discussion sections.

a) SAM/SPOT features are rotation and polar invariant:

As explained in our companion Methods paper NCOMMS-25-25126A, we construct SAM-SPOT features so that they are theoretically rotationally-invariant. In practice, there will be numerical deviations for example due to discretization, so that invariance is not exact. We expect SAM-SPOT features to be polar orientation invariant because inputs are centered before feature extraction. We don't expect mirror-invariance, and so if 180 degree rotations of an object is close to a mirrored version, this can break invariance numerically. Overall, we expect to be able to cluster all rotated instances of a unique object separate from rotations of a second unique object.

b) Benchmarking with MNIST handwriting dataset:

To test our claims empirically, we have performed additional benchmarks in the methods paper using the handwriting MNIST dataset, taking one image each for each of the 10 digits and generating rotationally perturbed, polar orientation perturbed (i.e. translating the digit) and left-right mirrored versions (Suppl. Fig. 1b, Methods paper NCOMMS-25-25126A). For these static datasets, we compute SA (shape-appearance) features and performed principal components analysis to visualize the clustering in 2D space. As can be seen, we correctly cluster all variations of the same digit of 0,1,2 as expected, all distinctly shaped, irrespective of perturbation. For the digit 2, we also see more variation and splitting of images rotated by +/-90 degrees vs +/-270 degrees, as expected. Surprisingly, we found polar displaced images to exhibit greater feature variation than rotated images. We are not currently sure what is driving this. Across all 10 digits, PCA analysis retains grouping of digits where +/- 90 degree vs +/-270 degree rotations maintain uniqueness, but splitting of point-clouds for those that are more ambiguous like 2 and 5, 6 and 9, 3 and 7. If exact numerical rotational invariances are required, or invariance to other transformations are required, at the expense of cost users can perform geometrical transformations of each input, and average their individual SAM features to obtain a mean SAM feature, similar to the thinking of ME-VAE. Doing this for each digit, with 90 degree rotations (i.e. 4 augmentations per image input), we almost completely abolish the numerical inaccuracies. The rotated, flipped and polar translated points of each digit now all cluster tightly together, and the PCA clearly identifies one point-cloud per digit. Using 45 degree in addition to 90 degree rotations (i.e. 8 augmentations per image input) further increases the invariance, and tightness of clustering. Verifying this is an effective strategy to improve the effectiveness of our SAM features.

III. The authors state that "Motion as combined changes in the object's shape, appearance, and centroid between consecutive timepoints". Mathematically, thinking SAM should capture mutually exclusive features of change, but due to their definition of motion, it implies that motion does not capture a distinct feature. How do the authors support the sufficiency of their definition of the motion?

Author's reply:

We apologize for the confusion in the general language used. Mathematically, we regard motion as the deformation field relating the image intensity at time t to time t+1, as computed by optical flow and the

translation displacement in centroids at time t to time t+1. Our motion features measure properties of the deformation field, namely its magnitude (speed) and to first-order, its decomposition into divergence and curl components (Helmholtz decomposition).

We don't enforce mutually exclusive features with shape and appearance, as the optical flow computation inevitably couples it to appearance and shape boundary. Meanwhile appearance should technically be measured independent of the shape, but this is non-trivial. Ideally, we could remap the texture into a fixed geometry like a circle, however any remapping of shape inevitably introduces distortion artefacts (also present in ME-VAE), and therefore distorted appearance. Moreover, these distortion artefacts are reflective of the original geometry. We are not currently aware of a technique which allow us to infinitely tile the texture without distortion. Finally, we could enforce mutual exclusivity of features after compiling of all phenomes e.g. through an orthogonal decomposition, and indeed our SAM module analysis which effectively groups mutually correlated features. However, we would then need some way to reassign modules as shape, appearance and motion which no longer may be 'pure' definitions. Currently, we should thus interpret the features as being derived from shape, appearance and motion considerations. Moving forwards, a next layer of analysis with the phenomes could be to derive SAM modes akin to orthogonal mode decomposition in shape analysis i.e. mutually exclusive joint shape-appearance-motion templates. We have added this to the discussion.

IV. How is it determined that rotation should always be ignored? Maybe a rotation is part of a meaningful motion? I am not sure about this but I put this as a question to be answered.

Author's reply:

We agree that rotation is a meaningful motion. Currently, our motion features do not explicitly compute the pure rotational motion as a feature. This decision was in part due to it not being clear how to include a rotation angle which is a 'directional' feature as a scalar feature to work with the rest of the SAM features. However, as described previously (see reply to 5.II), our motion features implicitly capture rotation and other potential spatial variations of the optical flow computed vector field. To demonstrate this we applied clockwise and anticlockwise 15 and 45 degree rotations to rotations of the same MNIST digits to simulate the next frame of each image. Suppl. Fig. 1c of our Methods paper NCOMMS-25-25126A shows that the motion field inferred by optical flow is dependent on the particular digit. We do not reconstruct a pure rotation, as optical flow is a non-rigid registration technique. Consequently, the PCA of the motion features primarily separates the digits, 0, 1, 2 and the magnitude of rotational motion is not well-differentiated. However, when we reperform the PCA with the motion features together with the shape and appearance features, we find separation of the digits, 0, 1, 2 and the emergence of a more consistent structure within each digit, where we find global directional alignment in PCA with increasing rotation magnitude i.e. a line joining the same image rotated +15 and +45 degree rotation, show the same direction as a line joining an image rotated -15 -to- 45 degree rotation. Thus, SPOT can distinguish the magnitude of rotational motion but not rotation orientation (clockwise (-) vs anticlockwise (+)). The substructure alignment is stronger when we further applied temporal order filtering in preprocessing the SAM features, as expected. To perform this filtering here, we regarded the rotated digits as part of a video with increasing time as rotation increased from 0-360 degrees. The substructure alignment was also stronger when we increase the invariance of shape-appearance features using the augmented SA features (4 rotations per image).

Future work will be needed to investigate how to reinstate the sign of the rotation and account for pure rotational and scaling motions i.e. we intend to introduce a more formal model decomposing motion into affine (translation, rotation, scale, shear) and non-rigid (optical flow after rigid-body registration) components.

V. The preprocessing contains removal of highly variant features "All dataset dependent preprocessing steps such as zero-valued and high variance features". I do not see why this is sensible. Conversely, I can see that high variance features are useful signals.

Author's reply:

We apologise for not making the point clear about zero-valued and high variance features. We have added the mathematical definition of a high variance feature and clarified the zero-valued features to Methods of the methods paper (NCOMMS-25-25126A).

We refer to a zero-valued feature as being zero across all object instances (i.e. all *N*). It therefore has zero variance, and zero information. We also test for potentially non-zero-valued features with zero variance across all object instance and therefore would also contribute zero information.

We agree with the reviewer that high-variance is indicative of signal. However, segmentation and tracking errors also contribute high variance e.g. an undersegmented cell will measure improbably low cell area, likewise a segmentation of two or more cells will measure significantly higher cell area. This will make this feature highly fluctuating such that we regard it as 'unreliable', and dominate the multidimensional analysis. To clarify, the variance criteria is applied to the standard deviation of the feature measured across all object instances i.e. a 'high variance' feature i is one where std(feat_i) >= mean(std(feat_i))_j + threshold_multiplier * std(std(feat_i))_j. This threshold measures variance across all features, and the threshold_multiplier is used to adjust the stringency of the cutoff as a multiplier of std. Typically, we consider this a normal variable and set threshold_multiplier to 2 or 3.

6. Is there enough detail provided in the methods for the work to be reproduced?

[Answer from the reviewer to the Editor] *Yes.*

Author's reply:

Thank you.
